# Intra-tumor genetic heterogeneity and alternative driver genetic alterations in breast cancers with heterogeneous *HER2* gene amplification

**DOI:** 10.1186/s13059-015-0657-6

**Published:** 2015-05-22

**Authors:** Charlotte KY Ng, Luciano G Martelotto, Arnaud Gauthier, Huei-Chi Wen, Salvatore Piscuoglio, Raymond S Lim, Catherine F Cowell, Paul M Wilkerson, Patty Wai, Daniel N Rodrigues, Laurent Arnould, Felipe C Geyer, Silvio E Bromberg, Magali Lacroix-Triki, Frederique Penault-Llorca, Sylvia Giard, Xavier Sastre-Garau, Rachael Natrajan, Larry Norton, Paul H Cottu, Britta Weigelt, Anne Vincent-Salomon, Jorge S Reis-Filho

**Affiliations:** Department of Pathology, Memorial Sloan Kettering Cancer Center, New York, NY 10065 USA; Department of Tumor Biology, Institut Curie, 75248 Paris, France; The Breakthrough Breast Cancer Research Centre, Institute of Cancer Research, London, SW3 6JB UK; Department of Pathology and CRB Ferdinand Cabanne, Centre Georges Francois Leclerc, 21000 Dijon, France; Departments of Anatomic Pathology and Oncology, Hospital Israelita Albert Einstein, São Paulo, 05652-900 Brazil; Department of Pathology, Institut Claudius Regaud, IUCT-Oncopole, 31059 Toulouse, France; Department of Pathology, Centre Jean Perrin, and University of Auvergne, 63000 Clermont Ferrand, France; Department of Pathology, Centre Oscar Lambret, 59000 Lille, France; Department of Medicine, Memorial Sloan Kettering Cancer Center, New York, NY 10065 USA; Department of Medical Oncology, Institut Curie, 75248 Paris, France; Affiliate Member, Human Oncology & Pathogenesis Program, Memorial Sloan Kettering Cancer Center, New York, NY 10065 USA; Affiliate Member, Computational Biology Center, Memorial Sloan Kettering Cancer Center, New York, NY 10065 USA

## Abstract

**Background:**

HER2 is overexpressed and amplified in approximately 15% of invasive breast cancers, and is the molecular target and predictive marker of response to anti-HER2 agents. In a subset of these cases, heterogeneous distribution of *HER2* gene amplification can be found, which creates clinically challenging scenarios. Currently, breast cancers with *HER2* amplification/overexpression in just over 10% of cancer cells are considered HER2-positive for clinical purposes; however, it is unclear as to whether the HER2-negative components of such tumors would be driven by distinct genetic alterations. Here we sought to characterize the pathologic and genetic features of the HER2-positive and HER2-negative components of breast cancers with heterogeneous *HER2* gene amplification and to define the repertoire of potential driver genetic alterations in the HER2-negative components of these cases.

**Results:**

We separately analyzed the HER2-negative and HER2-positive components of 12 HER2 heterogeneous breast cancers using gene copy number profiling and massively parallel sequencing, and identified potential driver genetic alterations restricted to the HER2-negative cells in each case. *In vitro* experiments provided functional evidence to suggest that *BRF2* and *DSN1* overexpression/amplification, and the *HER2* I767M mutation may be alterations that compensate for the lack of *HER2* amplification in the HER2-negative components of HER2 heterogeneous breast cancers.

**Conclusions:**

Our results indicate that even driver genetic alterations, such as *HER2* gene amplification, can be heterogeneously distributed within a cancer, and that the HER2-negative components are likely driven by genetic alterations not present in the HER2-positive components, including *BRF2* and *DSN1* amplification and *HER2* somatic mutations.

**Electronic supplementary material:**

The online version of this article (doi:10.1186/s13059-015-0657-6) contains supplementary material, which is available to authorized users.

## Background

Amplification and overexpression of the proto-oncogene *HER2* (*ERBB2*) are found in approximately 15 to 20% of all invasive breast cancers. HER2 positivity is defined either by immunohistochemistry (IHC), when >10% of cells show strong HER2 membrane staining (3+), or by fluorescence or chromogenic *in situ* hybridization (FISH/CISH), when the *HER2*:CEP17 ratio is ≥2 and/or *HER2* gene copy number is ≥6 [1]. HER2 is a *bona fide* driver gene in breast cancer [2-5], and *HER2* amplification is the predictive marker and molecular target of anti-HER2 agents such as trastuzumab, pertuzumab or lapatinib [6]. Recently, *HER2* somatic mutations have been identified in approximately 1.5% of all invasive breast cancers [7,8]. These mutations are preferentially found in a subset of HER2-negative breast cancers and have been shown to activate HER2 and its downstream signaling pathways and to constitute a potential mechanism of resistance to trastuzumab and lapatinib [7]. In fact, clinical trials testing irreversible HER2 inhibitors for the treatment of patients with breast cancers harboring *HER2* somatic mutations are currently ongoing (ClinicalTrials.gov: NCT01670877).

Massively parallel sequencing studies have revealed the complexity and heterogeneity of breast cancer. In particular, it has been demonstrated that the number of highly recurrently mutated genes, such as *TP53* and *PIK3CA*, is limited in breast cancer. On the other hand, there is a large collection of genes mutated in <3% of breast cancers [9,10], some of which are known driver genes or are targeted by mutations that have been associated with treatment resistance such as *HER2* [7] and *ESR1* [11-13]. In addition to the variation between tumors, intra-tumor genetic heterogeneity has also been documented in breast cancer [14,15], as illustrated by the identification of subclones, which harbor genetic alterations in addition to the founder genetic events present in all cells [9,16-18]. To circumvent the potential challenges posed by intra-tumor genetic heterogeneity, in particular for biomarker assessment and therapeutic decision-making, it has been suggested to focus on founder driver genetic events present in all cells of a given tumor (that is, the so-called ‘truncal drivers’) [19].

Whilst the majority of HER2-positive breast cancers show homogeneous patterns of *HER2* amplification and HER2 protein overexpression, intra-tumor heterogeneity in the form of two distinct or intermixed clones of breast cancer cells exhibiting different patterns of *HER2* gene amplification and overexpression can be observed [15,20]. The incidence of this phenomenon ranges from 1 to 40% depending on the methodology and cutoffs used [20-24]. We previously performed a re-review of a consecutive series of >600 HER2-positive breast cancers and 5% of these cases showed heterogeneous HER2 overexpression and gene amplification using clinical definitions of HER2-positivity [20]. It should be noted that when a breast cancer displays >10% of tumor cells harboring HER2 overexpression and gene amplification, it is diagnosed as HER2-positive and the patient is treated accordingly without a precise understanding of the clinical significance and the biological implications of this heterogeneity and of having a large proportion of the tumor composed of cells lacking HER2 overexpression/gene amplification. Furthermore, it is assumed that *HER2* amplification is the driver genetic alteration in these cancers; however, it is currently unknown whether the HER2-negative components of HER2 heterogeneous breast cancers harbor alternative genetic alterations. To address this question, we sought i) to define the clinico-pathologic characteristics of HER2 heterogeneous breast cancers, ii) to determine the somatic gene copy number alterations in the HER2-positive and HER2-negative components of HER2 heterogeneous breast cancers, iii) to define the repertoire of somatic mutations in the HER2-positive and HER2-negative components of HER2 heterogeneous breast cancers, and iv) to identify potential driver genetic alterations of breast cancer based on the analysis of the HER2-negative components of HER2 heterogeneous breast cancers. Here we show that HER2 heterogeneous breast cancers are estrogen receptor (ER)-positive and predominantly *TP53* mutant. In addition, we identified and functionally validated alternative driver genetic alterations restricted to the HER2-negative component of HER2 heterogeneous breast cancers, including *BRF2* and *DSN1* amplification, and a *HER2* somatic mutation.

## Results

### Clinico-pathologic characteristics of HER2 heterogeneous breast cancers

**Figure 1:**
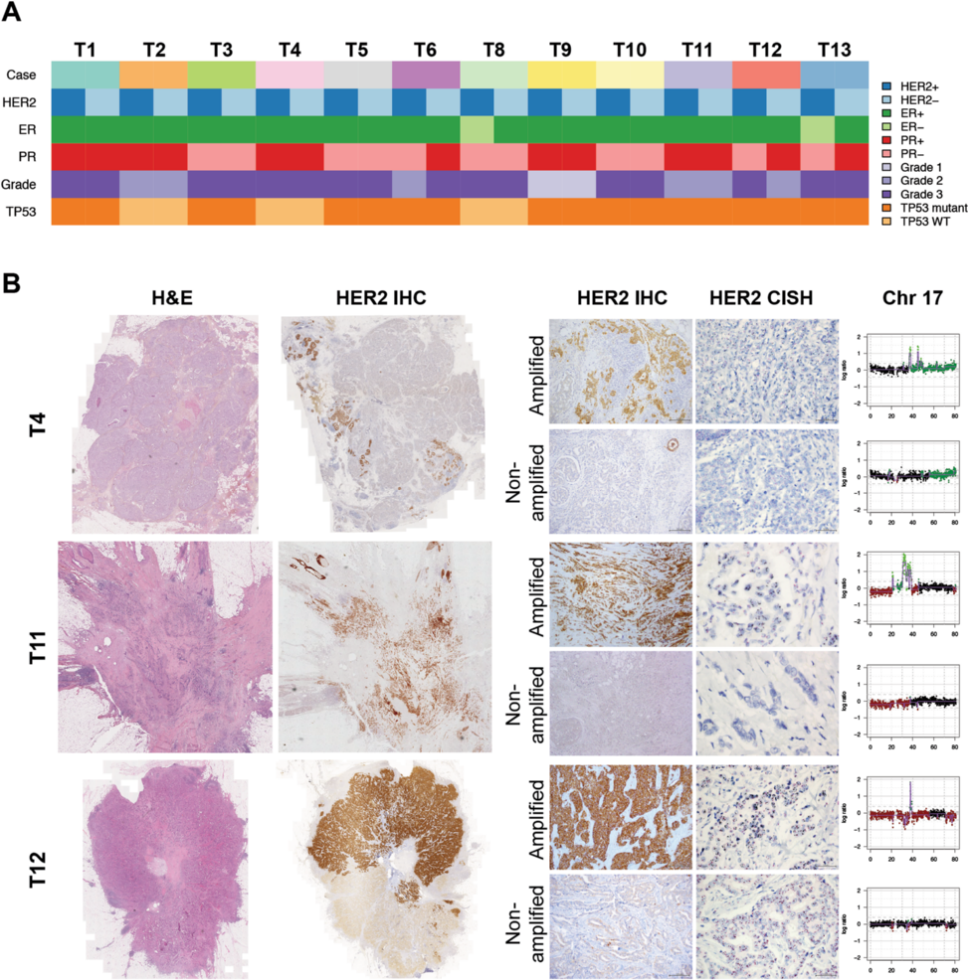
HER2 heterogeneous breast cancers are ER-positive and preferentially *TP53* mutant. **(A)** Clinico-pathologic characteristics of HER2 heterogeneous breast cancers included in this study. ER, estrogen receptor; PR progesterone receptor; WT, wild-type. **(B)** Micrographs of representative hematoxylin and eosin (H&E) stained sections, HER2 immunohistochemistry (IHC) and *HER2* chromogenic *in situ* hybridization (CISH) of selected HER2 heterogeneous breast cancers included in this study (scale bar IHC, 200 μm; scale bar CISH, 50 μm). Chromosome 17 plots of microdissected HER2-positive and HER2-negative components of each case confirming the presence and absence of *HER2* gene amplification (17q12), respectively. In the chromosome plots, the circular binary segmentation (cbs)-smoothed Log_2_ ratios for each bacterial artificial chromosome mapping to chromosome 17 were plotted on the y-axis and their genomic positions were plotted on the x-axis. Gains, amplifications and losses are highlighted in dark green, bright green and red, respectively. Please see Additional file 2 for the remaining cases.

We identified 41 HER2-positive breast carcinomas with heterogeneous HER2 overexpression and *HER2* gene amplification, of which 13 cases were amenable to microdissection. For 12 HER2 heterogeneous breast cancers the HER2-positive and HER2-negative components were separated without cross-contamination as confirmed by array-based comparative genomic hybridization (aCGH; Figure 1; Additional files 1 and 2). Histologic and immunohistochemical analysis revealed that all HER2 heterogeneous breast cancers subjected to microdissection were ER-positive (as defined by the current cutoff of >1% of ER-positive cells [25] in the whole tumor), and eight cases were also progesterone receptor (PR)-positive (Figure 1A; Additional file 1). The frequency of ER expression in these HER2 heterogeneous cases was significantly higher than that found in the HER2-positive breast cancers included in The Cancer Genome Atlas (TCGA; 56/79, 71%) dataset [10], the Molecular Taxonomy of Breast Cancer International Consortium (METABRIC) discovery (40/72, 56%) and validation (24/61, 39%) datasets [26], and the 1 year adjuvant trastuzumab versus observation for HER2-positive breast cancer (HERA) trial [27] cohort (1,536/3,383, 45%) (Fisher’s exact test, two-tailed, *P* = 0.0326, *P* = 0.0026, *P* < 0.0001, *P* < 0.0001, respectively). To define whether this observation related to the ER status of HER2 heterogeneous breast cancers would be generalizable, we investigated the ER status of the HER2 heterogeneous breast cancers that were retrieved for this study but not amenable to microdissection. The diagnostic ER immunohistochemical slides of 26 of the remaining 29 cases were retrieved, and re-analysis of their ER status revealed that 24 of 26 cases (92%) were ER-positive. The majority of the HER2 heterogeneous breast cancers in this study were of high histologic grade (9/12 grade 3, 75%, Additional file 1). Sanger sequencing revealed that all but three cases (75%) were *TP53* mutant (Figure 1A; Additional file 1), a frequency similar to that found in HER2-positive breast cancers from the TCGA dataset. When assessing the HER2-positive and HER2-negative components separately, we noted that in 10 out of 12 of the HER2 heterogeneous breast cancers the ER status and histologic grade were identical between the two components of a given case, as was the *TP53* status in all cases (Figure 1A; Additional file 1). Taken together, the HER2 heterogeneous breast cancers amenable to microdissection and included in this study were ER-positive and preferentially *TP53* mutant.

### Copy number alterations in HER2-positive and HER2-negative components of HER2 heterogeneous breast cancers

**Figure 2:**
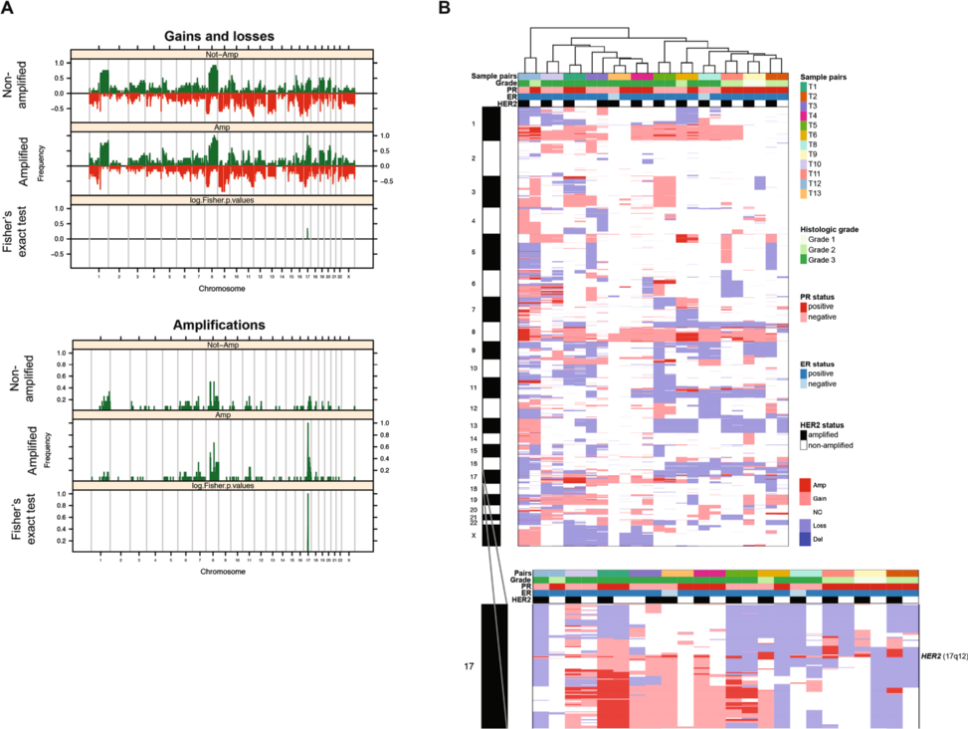
Gene copy number alterations in HER2-positive and HER2-negative components of HER2 heterogeneous breast cancers**. (A)** Frequency plots of copy number gains and losses (top) and of amplifications (bottom) in *HER2* non-amplified and *HER2*-amplified components of 12 HER2 heterogeneous breast cancers. The proportion of cases in which each bacterial artificial chromosome (BAC) clone is gained/amplified (green) or lost (red) is plotted (y-axis) for each BAC clone according to its genomic position (x-axis). Inverse Log_10_ values of the Fisher’s exact test *P*-values are plotted according to genomic location (x-axis) at the bottom of each graph. The only statistically significant difference identified between the genomic profiles of *HER2* non-amplified and *HER2*-amplified components of 12 HER2 heterogeneous breast cancers was *HER2* itself. **(B)** Hierarchical clustering of the genomic profiles of HER2-positive and HER2-negative components of 12 HER2 heterogeneous breast cancers. Hierarchical cluster analysis was performed with categorical states (that is, gains, losses, and amplifications) using Euclidean distance metric and the Wards algorithm. Amp, amplification; Del, deletion; NC, normal copy number.

To determine if there would be a common alternative gene copy number alteration (CNA) present in the HER2-negative components, which would account for the lack of *HER2* gene amplification, the microdissected HER2-positive and HER2-negative components of each case were subjected to DNA extraction and aCGH analysis (Figure 1B). When the copy number gains, losses and amplifications of the HER2-positive components were compared with those of the HER2-negative components from all 12 HER2 heterogeneous breast cancers, we observed that the patterns of CNAs were highly similar (Figure 2A). In fact, Fisher’s exact test revealed that the only recurrent CNA present at significantly different frequencies between the HER2-positive and HER2-negative components of the HER2 heterogeneous breast cancers studied here was the *HER2* amplicon itself (that is, 17q12). Furthermore, hierarchical cluster analysis of the categorical gene copy number states revealed that the HER2-positive and HER2-negative components of a given HER2 heterogeneous breast cancer clustered together, rather than all HER2-positive and all HER2-negative components clustering together (Figure 2B). These data suggest that the HER2-positive and HER2-negative components of each case are clonally related and that there is no highly recurrent alternative CNA in the HER2-negative components of HER2 heterogeneous breast cancers that compensates for the lack of *HER2* gene amplification.

**Figure 3:**
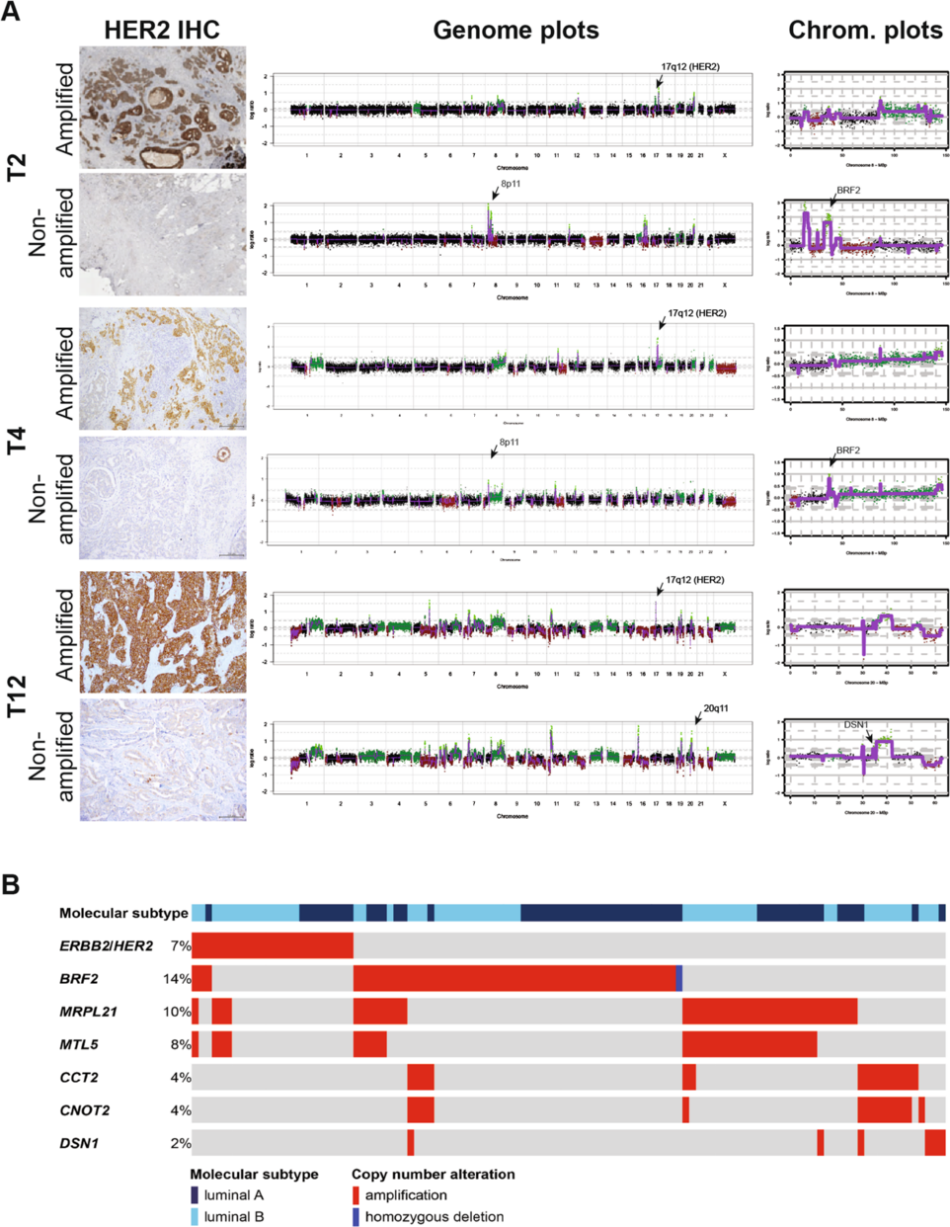
Identification of *BRF2* and *DSN1* amplification in HER2-negative components of HER2 heterogeneous breast cancers. **(A)** Gene copy number analysis of HER2-positive and HER2-negative components of HER2 heterogeneous breast cancers confirmed the presence and absence of *HER2* amplification (genome plots, middle), respectively, and identified the presence of an 8p11-p12 amplification, including *BRF2* and other breast cancer genes, in cases T2 and T4, and of a 20q11 amplification, encompassing *DSN1*, in case T12 restricted to the HER2-negative component of each case (chromosome plots, right). In the genome plots and chromosome plots, the genomic position is plotted along the x-axis and circular binary segmentation (cbs)-smoothed Log_2_ ratio on the y-axis; amplifications are shown in bright green, gains in dark green, losses in dark red and normal copy number in black. **(B)** Selected amplified genes identified to be restricted to the HER2-negative components of HER2 heterogeneous breast cancers were assessed in luminal breast cancers from the TCGA dataset (for complete list of genes and data source, see Additional file 6).

We next assessed the patterns of gene CNAs in a pairwise manner by comparing the HER2-negative and HER2-positive components of each case. Although the components of each HER2 heterogenous breast cancer were more similar to each other than to the components of any of the other cases (Figure 2B), we observed differences in their pattern of CNAs in addition to the *HER2* amplification. These differences were restricted to a few genomic regions in some tumors (for example, case T1), whereas in others, the two components were substantially different (for example, case T12) (Figure 2B; Additional file 3). This analysis further revealed specific amplifications, comprising 1,535 individual genes, restricted to the HER2-negative components of HER2 heterogeneous breast cancers (Figures 2B and 3A; Additional file 4). Some of these map to regions recurrently amplified in breast cancer, including the 1q24, 8p11-p12, 8q24, 11q13 and 20q13 amplicons, which have been shown to contain known driver genes [26,28-30]. To identify possible driver events in the HER2-negative components of HER2 heterogeneous breast cancers, of which >90% were ER-positive and of luminal A-like or luminal B-like subtype according to the St Gallen International Expert Consensus 2013 [31] (Additional file 1), the copy number status and gene expression of the 1,535 genes found to be amplified only in the HER2-negative components of the cases analyzed here were assessed in the luminal A and luminal B breast cancers of the TCGA dataset [10]. This list of genes contains numerous likely breast cancer drivers, including *MDM4* [32], *ZNF703* [33], *RAB11FIP1* [28,29], *MYC*, *FAM83A* [34], *PIK3CA*, *PROSC* [28], *PPAPDC1B* [35], *LSM1* [28,36], *BAG4* [36], *EEF1A2* [37], *CAMK1D* [38], *PHGDH* [39], *FGFR1* [40], *DDHD2* [28] and *WHSC1L1* [28,41] (Additional file 5). To prioritize the validation of novel potential driver genes based on the analysis of the HER2-negative components of HER2 heterogeneous breast cancers, we searched for genes i) whose expression is copy number regulated and are overexpressed when amplified, in a way akin to *HER2* itself [2,3], and ii) that are recurrently amplified in the dataset of HER2 heterogeneous cases and/or preferentially amplified in HER2-negative tumors in the TCGA luminal breast cancer dataset.

This analysis revealed that of the 1,535 amplified genes restricted to the HER2-negative components of our 12 HER2 heterogeneous breast cancers, 59 genes were statistically significantly copy number regulated in the luminal breast cancers of the TCGA dataset (Additional files 5 and 6), some of which were either recurrently amplified in the HER2-negative components of HER2 heterogeneous cases (for example, *BRF2* in cases T2 and T4) or mutually exclusively amplified with *HER2* in the TCGA luminal breast cancer dataset (for example, *DSN1* in case T12) (Figure 3A,B; Additional file 6).

**Figure 4:**
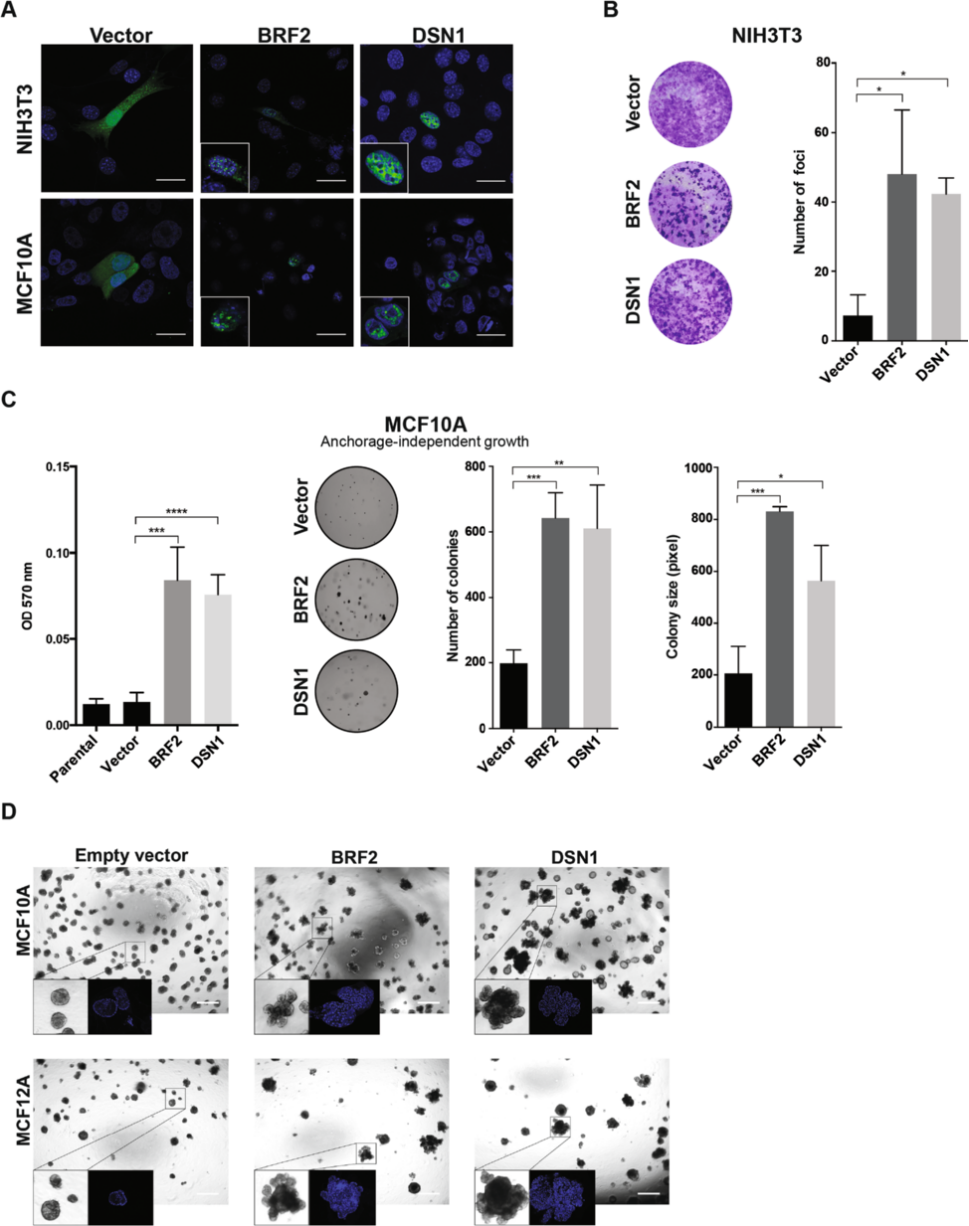
*BRF2* and *DSN1* amplifications are potential driver genetic alterations in HER2-negative breast cancer cells. **(A)** Nuclear subcellular localization of BRF2 and DSN1 in NIH3T3 (top) and MCF10A (bottom) cells expressing BRF2-ZsGreen and DSN1-ZsGreen (scale bar, 25 μm). **(B)** Foci formation assay of NIH3T3 cells expressing vector control, BRF2 or DSN1 protein. Cells were fixed and stained with crystal violet 21 days after plating, and the foci were quantified (see Materials and methods). *P < 0.05, unpaired t-test. Error bars represent standard deviation of mean. **(C)** Anchorage-independent growth of MCF10A cells expressing vector control, BRF2 or DSN1 protein. Quantification was performed using an MTT assay (left) or by defining the number and size of colonies (right). *P < 0.05, **P < 0.01, ***P < 0.001, ****P < 0.0001, unpaired t-test. Error bars represent standard deviation of mean. **(D)** Impact of empty vector, BRF2 and DSN1 expression on growth and glandular architecture of MCF10A (top) and MCF12A (bottom) cells grown in three-dimensional basement membrane cultures (scale bar, 500 μm).

From this list of genes, we focused on *BRF2* and *DSN1*, given the lack of direct functional evidence to support a potential oncogenic role of the amplification of these genes in breast cancer. Hence, we sought to define whether *BRF2* and *DSN1* would have oncogenic properties in *in vitro* models of breast cancer. *BRF2* (8p11.23) maps to the 8p11-p12 amplicon and is reported to be recurrently amplified in 10 to 15% of breast cancers [28]. This gene encodes a subunit of the RNA polymerase III transcription initiation factor and has been identified as a potential oncogene in lung squamous cell carcinomas [42]. *DSN1* maps to 20q11.23, and encodes a kinetochore protein of the minichromosome instability-12 centromere complex [43]. This gene is amplified only in 1.7% of all breast cancers, and its amplification is mutually exclusive with *HER2* amplification in the TCGA luminal breast cancer dataset (Figure 3B) [10]. The *DSN1* amplicon is distinct from the 20q13 amplicon, and is not encompassed by its smallest region of amplification [30]. Forced expression of BRF2 and DSN1 in NIH3T3 and non-malignant MCF10A breast epithelial cells resulted in their nuclear localization as expected (Figure 4A; Additional file 7), and in significant transformation of NIH3T3 and MCF10A cells as measured by a foci formation assay (Figure 4B) and anchorage-independent growth in soft agar (Figure 4C), respectively. In addition, forced expression of BRF2 and DSN1 in non-malignant breast epithelial cells MCF10A and MCF12A affected the growth and glandular architecture of these cells when grown in three-dimensional culture systems. Whilst empty vector-transfected MCF10A and MCF12A cells formed spheroid acinar-like structures, BRF2 and DSN1 overexpression led to larger, multiacinar structures with filled lumens (Figure 4D), in line with phenotypes previously observed when oncoproteins are expressed in this model system [44,45].

Taken together, the HER2-negative components of HER2 heterogeneous breast cancers are not underpinned by a single highly recurrent amplification. Our findings demonstrate, however, that some of the genes amplified only in the HER2-negative components of HER2 heterogeneous breast cancers, such as genes previously described as oncogenic, including *MDM4* [32], *FGFR1* [40,46], *ZNF703* [33], *MYC*, *FAM83A* [34], *RAB11FIP1* [28,29], and *PIK3CA* (Table 1), as well as *BRF2* and *DSN1*, which were shown to have oncogenic properties here, may constitute potential drivers and compensate for the lack of *HER2* amplification.

**Table 1:** **Potential driver genetic alterations in both HER2-negative and HER2-positive components of HER2 heterogeneous breast cancers, and in the HER2-negative components only**

**Sample ID**	**Potential driver mutations present in both HER2-negative and HER2-positive components**	**Potential driver mutations restricted to the HER2-negative component**	**Potential drivers within regions whose amplification was restricted to the HER2-negative component**
T1	*TP53* (P152L)		*FAM83A*, *MDM4*
T2	NP	NP	*BRF2*, *FGFR1*, *ZNF703*, *RAB11FIP1*, *LSM1*, *DDHD2*, *WHSC1L1*, *PPAPDC1B*, *EEF1A2*, *ERLIN2*, *BAG4*
T3	*TP53* (E258D)	*ATRX* (splice site dinucleotide substitution)	*YWHAZ*, *MYC*, *FAM83A*
T4	*ARID1A* (R1446*)		*BRF2*, *ZNF703*, *RAB11FIP1*, *ERLIN2*
T5	*TP53* (E286D)	NP	*IKBKB*, *CAMK1D*
T6	*TP53* (R273H), *PIK3CA* (H1047R)	*HER2* (I767M), *ETV5* (E60K)	*PHGDH*
T8	*PIK3CA* (H1047R), *CBFB* (splice site)	*BRAF* (P403S), *XRCC1* (S236F)	
T9	*TP53* (R282G), *PIK3CA* (H1047R), *MAP2K4* (R110G), *MED12* (R2015M)		*LMX1B*
T10	*TP53* (S94fs)	NP	*CBX3*, *RAD21*
T11	*TP53* (G187_E192delLAPPQ)	*NRP1* (R767H)	*MYC*, *RAD21*
T12	*TP53* (T195N), *KIT* (A755T)	*FANCD2* (L1394F)	*DSN1*
T13	*TP53* (S240I)	NP	*PIK3CA*

NP, massively parallel sequencing not performed due to lack of DNA of sufficient quantity and/or quality.

### Somatic mutations in HER2-negative components of HER2 heterogeneous breast cancers

**Figure 5:**
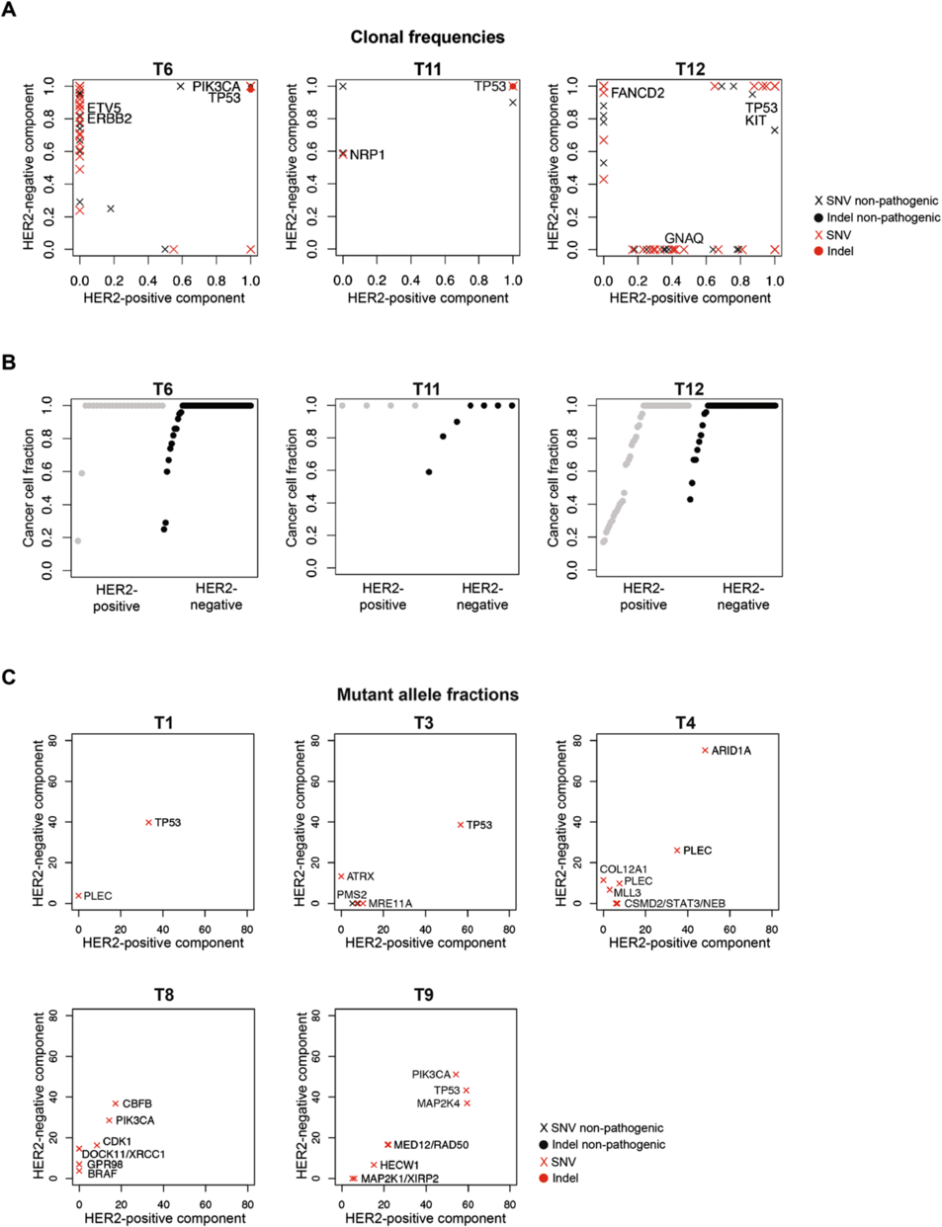
Sequencing analysis of HER2-positive and HER2-negative components of HER2 heterogeneous breast cancers identified founder genetic events and intra-tumor mutational heterogeneity. **(A)** Clonal frequencies of mutations identified in HER2-positive and HER2-negative components of HER2 heterogeneous breast cancers T6, T11 and T12, which were subjected to whole exome sequencing and orthogonal validation by amplicon sequencing (Ion Torrent) or targeted capture massively parallel sequencing (Illumina). Clonal mutation frequencies were estimated from the mutant allelic fractions adjusted according to tumor cellularity, tumor ploidy and local copy number states using ABSOLUTE [47]. Indel, insertion and deletion; SNV, single nucleotide variant. **(B)** Diagram illustrating the cancer cell fraction, as defined by ABSOLUTE, of mutations identified in cases T6, T11 and T12. Note the presence of subclonal mutations in the HER2-negative components of all cases, and in the HER2-positive components of cases T6 and T12. **(C)** Allelic fractions of mutations identified in HER2-positive and HER2-negative components of HER2 heterogeneous breast cancers obtained through targeted massively parallel sequencing analysis using a panel of 273 genes comprising genes frequently mutated in breast cancer and DNA repair-related genes. Indel, insertion and deletion; SNV, single nucleotide variant.

To determine if the constellations of mutations would be distinct between the HER2-positive and HER2-negative components of HER2 heterogeneous breast cancers, and to identify potential driver mutations restricted to the HER2-negative components, we subjected the HER2-positive and HER2-negative components of three cases (that is, T6, T11 and T12), for which sufficient DNA from frozen tumor and matched normal tissues was available, to whole exome sequencing. Selected somatic mutations identified were validated by high-depth amplicon sequencing (Ion Torrent, 4000×) or targeted massively parallel sequencing (Figure 5A; Additional files 8, 9 and 10). Analysis of the clonal frequencies using ABSOLUTE [47] revealed that known founder genetic events such as somatic mutations in *TP53* and/or *PIK3CA* were shared and inferred to be present in all cells of both the HER2-positive and HER2-negative components of these cases (Table 1; Figure 5A). This analysis also revealed the presence of subclonal mutations in the HER2-negative components of all cases, and in the HER2-positive component of cases T6 and T12 (Figure 5A,B). We next performed targeted massively parallel sequencing, using a panel of 273 genes frequently mutated in breast cancer and DNA repair-related genes [48], of the HER2-negative and HER2-positive components of 5 HER2 heterogeneous breast cancers (that is, T1, T3, T4, T8, and T9; Figure 5C). Consistent with the observations made by whole exome sequencing analysis, in all cases, the HER2-negative and HER2-positive components harbored somatic mutations in common, including *TP53* somatic mutations in three cases (Figure 5C). Interestingly, in the two *TP53* wild-type cases subjected to targeted sequencing, we identified an *ARID1A* mutation (that is, T4) and *PIK3CA* and *CBFB* mutations (that is, T8), which were common to the two components (Figure 5C). Taken together, in all cases analyzed, the HER2-negative and HER2-positive components shared identical somatic mutations, indicating their clonal relatedness.

**Figure 6:**
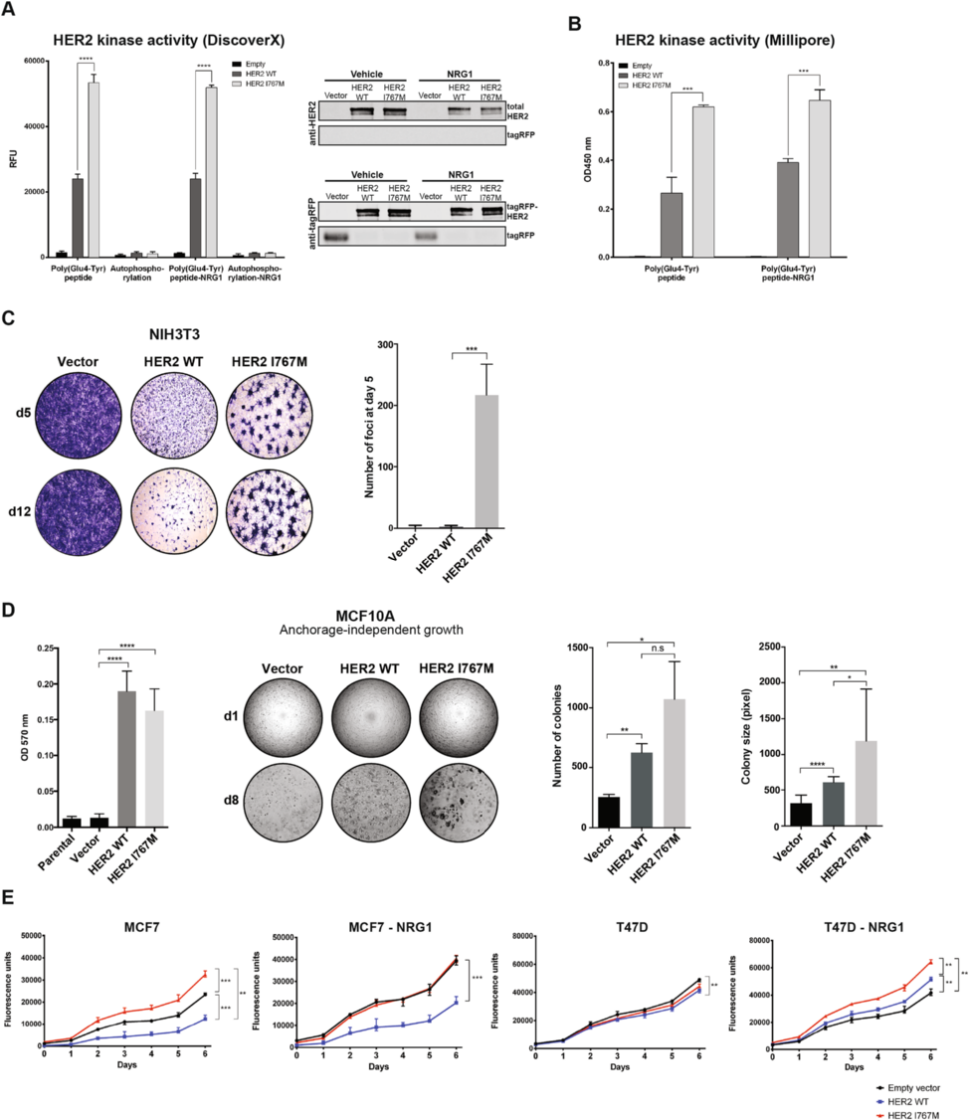
Identification of a *HER2* mutation as a potential driver genetic alteration in the HER2-negative component of a HER2 heterogeneous breast cancer. **(A)** Cell-free *in vitro* kinase assay determining the tyrosine kinase activity of the Poly(Glu4-Tyr) substrate and the autophosphorylation activity of wild-type (WT) HER2 (dark gray) and I767M mutant HER2 (light gray) in the presence and absence of neuregulin-1 (NRG1). Tyrosine kinase activity was assessed using the ADP Hunter HS Assay (DiscoveRx, left). Western blot analysis of representative elutes post-kinase assay of HER2-tagRFP, HER2(I767M)-tagRFP and tagRFP control proteins. The amounts of HER2 wild-type and I767M mutant HER2 enzymes used in the DiscoveRx kinase assay were similar as confirmed using antibodies against total HER2 (top panel) and tagRFP (bottom panel). ****P < 0.0001, Holm-Šídák-correction, multiple t-test. Error bars represent standard deviation of mean. **(B)** The Tyrosine Kinase Assay Kit (Millipore) confirmed the significantly higher transphosphorylation of the tyrosine kinase substrate Poly(Glu4-Tyr) by I767M mutant HER2 (light gray) compared with wild-type HER2 (dark gray). ***P < 0.001, Holm-Šídák-correction, multiple t-test. Error bars represent standard deviation of mean. **(C)** Foci formation assay of NIH3T3 cells stably expressing empty vector, wild-type HER2 or I767M mutant HER2 protein. Cells were fixed and stained with crystal violet 5 and 12 days after plating. Quantification was performed at day 5. Note that at day 12, the wild-type HER2 resulted in increased foci formation. ***P < 0.001, unpaired t-test. Error bars represent standard deviation of mean. **(D)** Anchorage-independent growth of MCF10A cells stably expressing empty vector, wild-type HER2 or I767M mutant HER2 protein. Quantification was performed using an MTT assay (left) or by defining the number and size of colonies (right). *P < 0.05, **P < 0.01, ****P < 0.0001, unpaired t-test. N.s., not significant. Error bars represent standard deviation of mean. **(E)** Effect of stable expression of wild-type HER2 (blue) and I767M mutant HER2 (red) on survival and growth of ER-positive MCF7 (*PIK3CA* mutant) and T47D (*PIK3CA* and *TP53* mutant) cells in growth media with or without neuregulin-1 (NRG1, 10 ng/ml). **P < 0.01, ***P < 0.001, Holm-Šídák-correction, multiple t-test. Error bars represent standard deviation of mean.

When focusing on the mutations restricted to the HER2-negative components, we identified a *HER2* I767M somatic mutation in one of the three cases subjected to whole exome sequencing (T6; Figure 5A; Additional file 11). *HER2* somatic mutations have been shown not to result in HER2 overexpression using the current immunohistochemical assays [7]. The *HER2* I767M kinase domain mutation has previously been reported to increase the levels of HER2 phosphorylation modestly in MCF10A cells [7], but it has not been further evaluated for its potential as an activating driver event. Here we demonstrate using two independent cell-free kinase assays that the HER2 I767M mutation displayed significantly increased transphosphorylation of the tyrosine kinase substrate Poly(Glu4-Tyr) compared with wild-type HER2 (Figure 6A,B). We next investigated whether the HER2 I767M mutation would result in transformation of NIH3T3 cells. Stable forced expression of the HER2 I767M mutation resulted in significantly increased foci formation compared with empty vector and wild-type HER2 (Figure 6C). To define the impact of the HER2 I767M mutation on anchorage-independent growth, we generated MCF10A cells stably expressing the empty vector, wild-type HER2 and the HER2 I767M mutation. Soft agar assays revealed that both wild-type and I767M mutant HER2 led to an increase in the number of colonies compared with empty vector; however, the HER2 I767M mutation resulted in significantly larger colonies than those caused by wild-type HER2 (Figure 6D). Similar results were obtained with NIH3T3 cells stably expressing the empty vector, wild-type HER2 and the HER2 I767M mutation (Additional file 12). To define whether the HER2 I767M mutation would have an impact similar to that of HER2 tyrosine kinase mutations previously shown to be strongly activating (V777L) or not activating (Y835F) by Bose *et al*. [7], we transiently forced the expression of empty vector, wild-type HER2, HER2 I767M, HER2 V777L and HER2 Y835F in NIH3T3, MCF10A and MCF12A cells. These transiently transfected cells were subsequently subjected to a soft agar assay which demonstrated that the HER2 I767M mutation resulted in anchorage-independent growth in soft agar that was higher than that caused by wild-type HER2 and the non-activating HER2 mutation (Y835F), but not statistically different from that caused by the strongly activating mutation (V777L) (Additional file 13).

**Figure 7:**
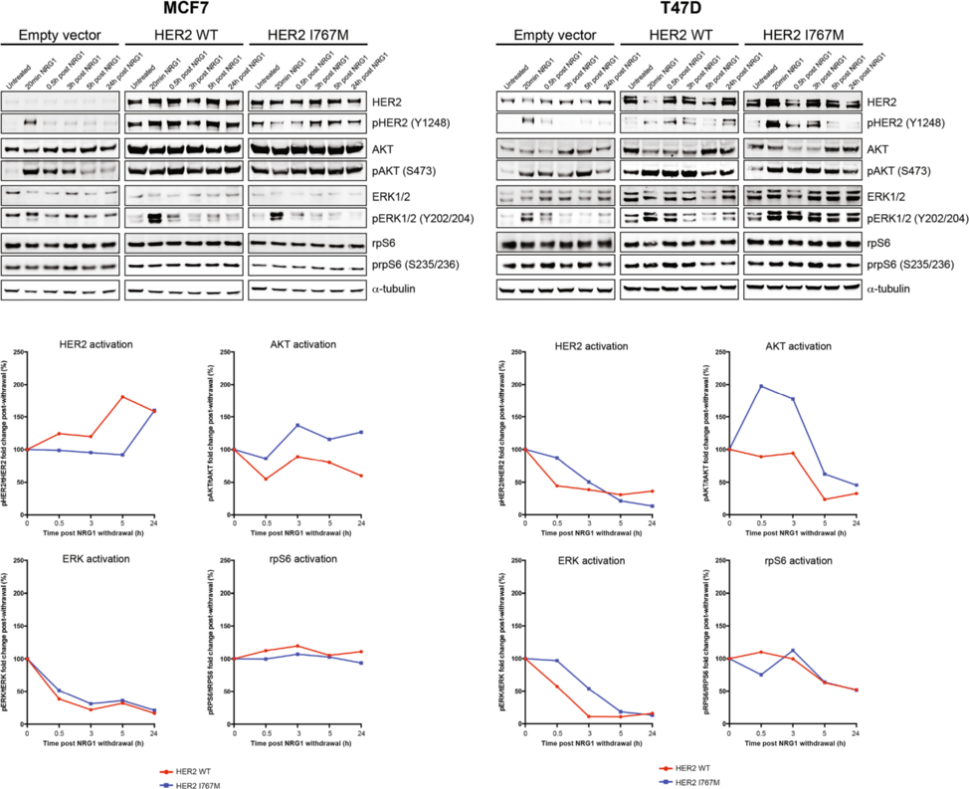
Signaling pathway activation of ER-positive MCF7 and T47D cell lines expressing wild-type (WT) or I767M mutant HER2. Whole-cell lysates of MCF7 and T47D, stably expressing empty vector control, wild-type HER2 or I767M mutant HER2 were analyzed by western blotting for total and phosphorylated levels of HER2, AKT, ERK1/2 and rpS6 on the same membrane, detected by near infrared two-color detection and quantified (LI-COR; Odyssey). Phospho-/total protein ratios are shown below. NRG1, neuregulin-1.

Given that all HER2 heterogeneous breast cancers subjected to sequencing here were ER-positive, and all but one case was *TP53* and/or *PIK3CA*-mutant, we assessed the effect of forced expression of the HER2 I767M mutation on the growth and HER2 downstream signaling in ER-positive breast cancer cell lines harboring *TP53* and/or *PIK3CA* mutations (that is, MCF7, *PIK3CA*-mutant; T47D, *TP53* and *PIK3CA*-mutant). Upon forced expression, both wild-type and mutant HER2 protein were found to be expressed in the membranous subcellular fraction of stably transfected breast cancer cell lines (Additional file 14). In MCF7 cells, expression of HER2 I767M led to a significant advantage in cell growth compared with empty vector when cultured in standard growth media (Figure 6E). By contrast, forced expression of HER2 I767M in T47D cells resulted in a significant growth advantage in comparison to T47D cells expressing wild-type HER2 or empty vector control only in the presence of neuregulin-1, an activator of HER receptors [49] (Figure 6E). To determine whether these differences would stem from distinct levels of endogenous neuregulin-1 in MCF7 and T47D cells, we assessed neuregulin-1 protein and mRNA expression levels in these cells by western blotting and quantitative RT-PCR, respectively; no differences in the levels of neuregulin-1 protein expression in these cells and their conditioned media and of *NRG1* mRNA in samples extracted from MCF7 and T47D cells were detected (Additional file 15). To determine the effect of forced wild-type HER2 and HER2 I767M expression on downstream effector pathways in MCF7 and T47D cells, we performed a time-course experiment where the activation of HER2, AKT, ERK1/2 and ribosomal protein S6 (rpS6) was determined at baseline, after 20 minutes of neuregulin-1 treatment, and 30 minutes, 3 hours, 5 hours and 24 hours post-neuregulin-1 withdrawal using quantitative infrared fluorescent western blotting (LI-COR; Figure 7). At baseline, cells expressing wild-type HER2 and HER2 I767M were found to display similar levels of activation of the AKT and MAPK pathways (Figure 7). Although neuregulin-1 stimulation resulted in similarly increased levels of activation of AKT and ERK1/2 in cells expressing wild-type HER2 and the HER2 I767M mutation, we observed that the activation of AKT was sustained for longer in cells expressing the HER2 I767M mutation than in cells expressing wild-type HER2 (Figure 7).

**Figure 8:**
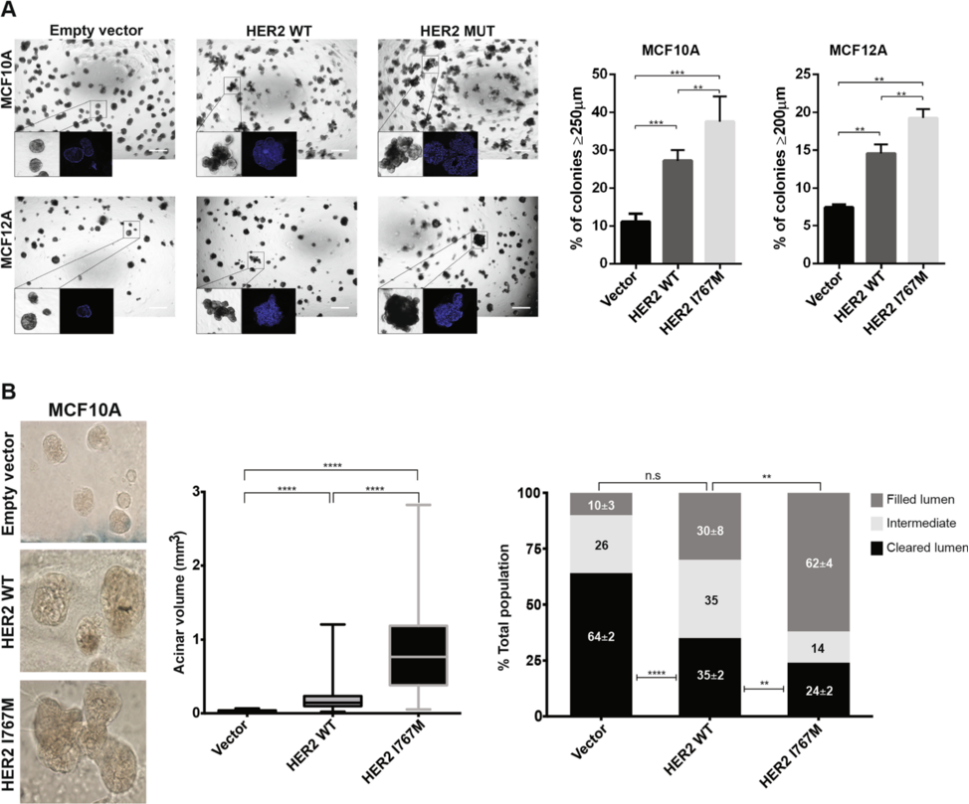
Impact of the HER2 I767M mutation on glandular architecture of non-malignant breast epithelial cells. **(A)** Impact of transient expression of empty vector, wild-type HER2 (WT) and I767M mutant HER2 on growth and glandular architecture of MCF10A (top) and MCF12A (bottom) cells grown in three-dimensional basement membrane cultures (scale bar, 500 μm). The percentage of acinar structures ≥250 μm (MCF10A) and ≥200 μm (MCF12A) were quantified. **P < 0.01, ***P < 0.001, unpaired t-test. Error bars represent standard deviation of mean. **(B)** Impact of stable expression of empty vector, wild-type HER2 and I767M mutant HER2 on acinar size and lumina filling of MCF10A cells. Representative micrographs are shown (original magnification 40×). MCF10A cells expressing I767M mutant HER2 formed significantly larger acinar structures (left), which significantly more frequently displayed filled lumina (right) compared with MCF10A cells stably expressing empty vector or wild-type HER2. **P < 0.01, ****P < 0.0001, unpaired t-test. N.s., not significant. Error bars represent standard deviation of mean.

To assess the effect of the HER2 I767M mutation on acinar structures, non-malignant breast epithelial cells expressing wild-type HER2 and HER2 I767M were grown in basement membrane cultures. In line with previous reports [44], wild-type HER2 expression in MCF10A cells elicited a multiacinar phenotype compared with the spherical structures of MCF10A empty vector cells (Figure 8A). Expression of HER2 I767M in MCF10A cells led to a significantly higher number of large multiacinar structures with increased branching and filled lumens than the expression of wild-type HER2 (Figure 8A). Furthermore, while forced expression of wild-type HER2 in MCF12A cells led to irregular structures compared with empty vector MCF12A spheroids, HER2 I767M MCF12A structures were significantly more frequently larger (Figure 8A). In MCF12A cells, these structures displayed luminal filling and irregular contours, and some showed infiltrating edges (Additional file 16). The phenotype induced by forced expression of HER2 I767M is consistent with that observed when other oncoproteins are expressed in this model system [44,45]. To validate these observations, MCF10A cells stably expressing empty vector, wild-type HER2 and the HER2 I767M mutation were grown in the same three-dimensional culture system. This analysis confirmed that stable forced expression of the HER2 I767M mutation resulted in significantly larger acini, whose lumina were significantly more frequently filled than those observed in cells expressing the empty vector or wild-type HER2 (Figure 8B). These observations suggest that case T6 is an example of a convergent phenotype, where HER2 is activated by two different mechanisms in a breast cancer; whilst the HER2-positive component is driven by *HER2* amplification, the HER2-negative component is likely driven by a HER2 tyrosine kinase mutation.

Taken together, the analysis of all somatic CNAs and mutations in HER2 heterogeneous breast cancers not only revealed the founder genetic events (that is, somatic genetic alterations likely present in all cells) of these tumors, including *TP53* and *PIK3CA* mutations, but also led to the identification of at least one potential alternative driver genetic alteration restricted to the HER2-negative component in the breast cancers analyzed (Table 1). In addition to the genetic alterations discussed above, that is, *BRF2* amplification, *DSN1* amplification and *HER2* I767M somatic mutation, we identified an *ATRX* splice site dinucleotide substitution (case T3), a *BRAF* P403S potentially pathogenic mutation (case T8), and a *FANCD2* L1394F potentially pathogenic mutation (case T12), and amplification of the candidate oncogene *FAM83A* [34] (cases T1 and T3), of *MYC* (cases T3 and T11), and of *PIK3CA* (case T13) amongst others (Table 1), which were present exclusively in the HER2-negative components of these HER2 heterogeneous breast cancers.

## Discussion

Our study demonstrates that intra-tumor genetic heterogeneity is not restricted to passenger genes but that in breast cancer also *bona fide* driver genetic alterations such as *HER2* gene amplification can be heterogeneously distributed within a given tumor. Here, we have identified potential alternative driver genetic alterations that are present only in the HER2-negative and are absent in the HER2-positive components of HER2 heterogeneous breast cancers. These potential alternative driver alterations were found to affect known cancer genes (Table 1), including amplification of *PIK3CA* or *MYC*, and likely driver genes mapping to the 8p11-p12 amplicon, including *ZNF703* [33], *RAB11FIP1* [28,29], *LSM1* [28], *PPAPDC1B* [35], *WHSC1L1* [28,41] and *FGFR1* [40,46]. Here we also provide functional evidence to suggest that the copy number regulated genes *BRF2*, a TFIIB-like factor mapping to the 8p11-p12 amplicon [28,42], and *DSN1*, a kinetochore protein mapping to 20q11 [43], as well as the *HER2* I767M mutation may confer a neoplastic advantage to HER2-negative breast epithelial cells.

The HER2-positive breast cancers with heterogeneous patterns of HER2 overexpression and gene amplification studied here were all ER-positive, and most were of high histologic grade (75%) and harbored somatic *TP53* mutations (75%). We noted that this phenotype resembles that of breast cancers arising in *TP53* germline mutation carriers (that is, Li-Fraumeni syndrome), which have been shown to be preferentially ER-positive, HER2-positive and of high histologic grade [50]. The implications of this similarity in the histopathologic profile between breast tumors arising in *TP53* germline mutation carriers and the HER2 heterogeneous breast cancers studied warrants further investigation. Interestingly, in the cases harboring *TP53* somatic mutations analyzed here, these mutations were found to be likely founder genetic events, potentially preceding the amplification of *HER2*, in a way akin to the *TP53* germline mutations preceding *HER2* amplification in breast cancers from Li-Fraumeni patients.

*HER2* amplification and overexpression have been suggested to be an early event in breast tumorigenesis [51]. It is not clear whether in the HER2 heterogeneous breast cancers studied here, *HER2* amplification was an early event and subsequently lost in the HER2-negative components, or whether *HER2* amplification was acquired in the HER2-positive components at a relatively late stage of tumorigenesis. It could be posited that the presence of *bona fide* driver genetic alterations shared by the HER2-positive and HER2-negative components in all cases subjected to sequencing (Table 1) would be suggestive of the *HER2* amplification being a relatively late event in the development of these tumors. For example, a possible explanation for the findings observed in case T6 is that the *TP53* and *PIK3CA* mutations were the truncal drivers, whereas the *HER2* gene amplification and the *HER2* I767M mutation would constitute two distinct branch drivers. Alternatively, loss of *HER2* amplification in one of the components could be the result of the acquisition of another driver genetic alteration that is in epistatic interaction [52] with *HER2* amplification. This scenario, albeit possible, would likely require the *HER2* amplicon to be syntenic in tumors with heterogeneous *HER2* amplification. Whole genome sequencing analysis and fiber fluorescence *in situ* hybridization, which can be performed in fresh/frozen samples, could be employed to define whether the *HER2* amplicon is syntenic or distributed in multiple chromosomal locations in HER2 heterogeneous cancers.

It has been reported previously that patients whose tumors display intra-tumor genetic heterogeneity of *HER2* gene amplification have a shorter progression-free survival than patients with homogeneous *HER2* gene amplification [22]. Furthermore, we have described previously that lymph node or distant metastases from patients with primary HER2 heterogeneous breast cancers treated with anti-HER2 agents and chemotherapy may be HER2-positive or HER2-negative, suggestive of clonal selection [20]. Together with the findings in this study that alternative driver genetic alterations may be present in the HER2-negative components of HER2 heterogeneous breast cancers, our data may provide an explanation for the presence of discordant HER2 status between primary tumors and metastases when assessed on biopsy material, which has been found in up to 14% of cases [53]. Importantly, analysis of the distant relapses of cases T4 and T8 after trastuzumab and chemotherapy treatment revealed that whilst in the former the contralateral axillary relapse was *HER2* amplified and displayed a pattern of gene CNAs similar to that of the HER2-positive component of the primary tumor, the cutaneous chest wall metastasis of T8 following trastuzumab and chemotherapy treatment was *HER2* non-amplified (Additional files 17 and 18). In the era of precision medicine, it may be of importance to acknowledge the existence of HER2 heterogeneity in tumors and to take this information into account when biopsies of HER2-positive breast cancers are subjected to genomic analyses, as the repertoire of somatic genetic alterations may differ between the HER2-positive and HER2-negative components.

Recent studies have suggested that mutations affecting driver genes and driver genetic alterations are homogeneously distributed within some cancer types (for example, *EGFR*, *KRAS*, and *TP53* mutations in non-small cell lung cancers) [54,55]. On the other hand, in triple-negative (that is, ER-negative, PR-negative and HER2-negative) breast cancers, even mutations affecting *bona fide* driver genes, such as *TP53*, have been shown to be heterogeneously distributed in a subset of cases [9]. Here, we provide direct evidence to demonstrate that, like triple-negative breast cancers [9], a subset of HER2-positive cancers are mosaics at diagnosis, and the subpopulations of cancer cells in these tumors may differ on the basis of the presence of *HER2* gene amplification, their perceived main driver, in addition to the presence of subclonal mutations affecting other genes.

Our study has several limitations. Nine of the patients with HER2 heterogeneous breast cancer included in this study received adjuvant trastuzumab, of whom two had relapses to date (Additional files 1, 17 and 18). Given the limited follow-up and number of relapses, the impact of the intra-tumor HER2 heterogeneity on response to anti-HER2 therapy and outcome could not be assessed. Furthermore, the number of HER2 heterogeneous breast cancers included in this study is small, due to the relative rarity of HER2 heterogeneous cases where each component could be adequately microdissected for downstream genomics analyses. The statistical power to identify recurrent driver genetic alterations in the HER2-negative components that were present in <30% of cases was therefore limited. However, in all cases potential alternative driver events were identified, suggesting that in the absence of *HER2* amplification, there are several distinct genetic alterations that may drive the HER2-negative components of HER2 heterogeneous cases. Although we have validated functionally three somatic genetic alterations found in the HER2-negative components of the HER2 heterogeneous cases analyzed, several additional genes identified as amplified or somatically mutated in the HER2-negative components were documented (Table 1). Further studies investigating their potential role as drivers of HER2-negative cancers are warranted. Finally, the mechanistic basis of the HER2 genetic heterogeneity documented in this study remains to be defined; it should be noted, however, that this study provides direct evidence to demonstrate that a known driver and clinically actionable somatic genetic alteration (that is, *HER2* gene amplification) can be heterogeneously distributed within breast cancers classified as HER2-positive by clinical definitions.

## Conclusions

A subset of HER2-positive breast cancers show heterogeneous *HER2* amplification and harbor distinct driver genetic alterations in the different components. Detailed genomic analyses of these components coupled with *in vitro* experiments resulted in the identification of *BRF2* and *DSN1* amplification and *HER2* I767M somatic mutation as potential novel breast cancer driver genetic events. Given that in HER2 heterogeneous breast cancers, *HER2* gene amplification and protein overexpression, their perceived driver and therapeutic target, may be present only in a subset of cancer cells, our findings have important implications for the delivery of targeted therapies. Harnessing the information stemming from the genetic heterogeneity affecting genes that are *bona fide* drivers of breast cancer and the chronology of genetic alterations in cancer is germane to the realization of the potentials of precision medicine.

## Materials and methods

### Sample selection

Breast cancers diagnosed as HER2-positive but showing HER2 heterogeneous overexpression were selected and retrieved from the authors’ institutions and re-reviewed by three pathologists (AG, AV-S and JSR-F). In brief, over 250 HER2-positive breast cancers diagnosed at Institut Curie, Paris, France between 2005 and 2008 and treated with conservative surgery as a first step of treatment were reviewed at Institut Curie and cases showing a heterogeneous overexpression pattern for HER2 defined as >10% but <100% of cells displaying HER2 overexpression in the form of strong, complete membrane staining were identified. Furthermore, cases with similar staining patterns were obtained from four French Comprehensive Cancer Centers (Centre Georges François Leclerc, Dijon; Institut Claudius Regaud, Toulouse; Centre Jean Perrin, Clermont-Ferrand; Centre Oscar Lambret, Lille), and from Hospital Israelita Albert Einstein, São Paulo, Brazil, and submitted for further review at Institut Curie, Paris, France. Analysis of human samples was performed in accordance with the French Bioethics Law 2011-814, the French National Institute of Cancer (INCa) Ethics Charter and after approval by the Institut Curie Review Board and Ethics committee (Comité de Pilotage du Groupe Sein; project 'Repertoire des alterations genetiques somatiques dans les adenocarcinomes mammaires avec heterogeneite du statut HER2', final version approved on 18 June 2013). Written consent was obtained from patients whose samples were subjected to massively parallel sequencing. Cases were anonymized prior to genomic profiling and massively parallel sequencing analyses. This study is compliant with the Declaration of Helsinki.

### Immunohistochemistry and chromogenic *in situ* hybridization

Formalin-fixed, paraffin-embedded sections of the selected HER2 heterogeneous breast cancers were cut at 3 μm, and immunohistochemistry performed for ER, PR and Ki67 using the antibodies and antigen retrieval methods described in Duprez *et al*. [56]. For confirmation, tumor sections were stained for HER2 using the HercepTest (Dako, Glostrup, Denmark) [57]. Scoring was performed by two pathologists (AV-S and JSR-F) according to the American Society of Clinical Oncology (ASCO)/College of American Pathologists (CAP) guidelines [1,25]. Only cases with distinct areas of HER2 3+ positivity with adjacent areas of HER2 1+ positivity or absent HER2 overexpression were included (n = 41). Cases with admixed levels of HER2 overexpression, including HER2 2+ (that is, equivocal), were excluded. In addition to the local FISH analyses performed, *HER2* gene amplification was confirmed by CISH, which allowed for the assessment of *HER2* gene copy number status and its distribution in the neoplastic tissues. CISH was performed using the ZytoDot 2C SPEC HER2/CEN 17 Probe Kit (Zytovision GmbH, Bremerhaven, Germany) or the HER2 CISH pharmaDX kit (Dako) according to the manufacturers’ instructions. *HER2* gene amplification was defined according to ASCO/CAP guidelines [1] and assessed by three pathologists (AG, AV-S and JSR-F). Tumors were graded according to the Nottingham grading system [58] by three pathologists (AG, AV-S and JSR-F).

### Microdissection and DNA extraction

Of the cases reviewed and selected, 13 breast cancers with heterogeneous HER2 protein overexpression and *HER2* gene amplification by central IHC and CISH were amenable to microdissection, as HER2-positive and HER2-negative areas were sufficiently discrete. The HER2-positive and HER2-negative components were microdissected either on a PixCell II laser capture microdissector (Arcturus, Life Technologies, Paisley, UK) into separate tubes from 8 μm-thick representative tissue sections stained with HercepTest for guidance, or using a sterile needle under a stereomicroscope in selected cases as previously described [59]. DNA was extracted from the microdissected HER2-positive and HER2-negative components using DNeasy Blood and Tissue kit (Qiagen, Crawley, UK), and quantity was assessed using a PicoGreen assay (Life Technologies, Paisley, UK).

### Microarray-based comparative genomic hybridization

DNA obtained from the microdissected HER2-positive and HER2-negative components of 13 HER2 heterogeneous breast cancers was subjected separately to aCGH, using a 32K bacterial artificial chromosome (BAC) array platform with 50 kb resolution [56,57]. This platform has been shown to be as robust as, and to have comparable resolution with, high-density oligonucleotide arrays [60-62] and to perform well with DNA extracted from formalin-fixed paraffin-embedded tissue samples. DNA labeling and hybridization, image acquisition and data analysis were performed as previously described [56,57] (Additional file 19). The aCGH analysis script and code are available in Additional file 20.

### Whole exome sequencing and targeted sequencing

Microdissected frozen samples of the HER2-positive component, the HER2-negative component and the matched normal tissue from three cases (T6, T11 and T12) were subjected to whole exome sequencing (Agilent SureSelect, Santa Clara, CA, USA) on an Illumina Genome Analyzer IIx or Illumina HiSeq2000 platform to a median coverage of 81× (Additional files 8 and 19). Candidate somatic variants with mutant allele frequencies >15% identified by whole exome sequencing in at least one component were subjected either to deep re-sequencing on an Ion Torrent platform (Life Technologies), or to targeted capture massively parallel sequencing on an Illumina HiSeq2000 (Additional file 19). Clonal mutation frequencies were inferred using ABSOLUTE [47]. In addition, five of the twelve cases subjected to aCGH profiling had sufficient DNA from tumor and normal tissues to be subjected to custom 273 gene paired-end massively parallel targeted sequencing on an Illumina HiSeq2000 essentially as previously described [48]. Details of the coverage and depth obtained in each component of these cases are described in Additional file 8. The strategy for the classification of mutations according to their pathogenicity is outlined in Additional file 19.

### Sanger sequencing

Sanger sequencing of exons 1 to 11 of *TP53* was performed in the HER2-positive and HER2-negative components of all HER2 heterogeneous breast cancers as previously described [48] (for primer sequences, see Additional file 21). A perfect agreement between the results of *TP53* Sanger sequencing and *TP53* mutation status as defined by massively parallel sequencing was observed for the cases analyzed.

### Cell lines

MCF10A, MCF12A, MCF7, T47D, BT474, HEK293T and NIH3T3 cells were purchased from the American Type Culture Collection (ATCC), authenticated by short tandem repeat profiling as previously described [63], and tested for mycoplasma infection using a PCR-based test (ATCC). Culture conditions are described in Additional file 19.

### Vector construction, mutagenesis, transformation and plasmid preparation

The human *ERBB2* (NM_004448) cDNA ORF clone pCMV6-ERBB2::Myc-DDK was purchased from Origene (RC212583, Rockville, MD, USA), and the I767M mutation introduced using the GeneArt Site Directed Mutagenesis Kit (Life Technologies) following the manufacturer’s recommendations (pCMV6-ERBB2(I767M)::Myc-DDK). *ERBB2* (*HER2*) wild-type and mutant (I767M) open reading frames were cloned into the pCMV6-TagRFP vector to generate pCMV6-ERBB2::TagRFP and pCMV6-ERBB2(I767M)::TagRFP plasmids, respectively, and into the pLenti-EF1a-GFP-2A-Puro vector (LV067, ABM, Richmond, BC, Canada), to generate the pLenti-ERBB2 and pLenti-ERBB2(I767M) lentiviral plasmids, as previously described [45] (Additional file 19). *BRF2* and *DSN1* open reading frames were amplified from total RNA derived from a healthy donor using SuperScript III First Strand Synthesis System and Platinum Taq polymerase High Fidelity (Life Technologies), and cloned into the pCMV6-ZsGreen vector to generate pCMV6-BRF2::ZsGreen and pCMV6-DSN1::ZsGreen plasmids, respectively, and into the pLenti-EF1a-GFP-2A-Puro vector (LV067, ABM), generating the pLenti-BRF2 and pLenti-DSN1 lentiviral plasmids, respectively. Sanger sequencing was used to confirm the reading frames of the wild-type *ERBB2*, the I767M mutant *ERBB2*, and wild-type *BRF2* and *DSN1*. Primer sequences are available in Additional file 21.

### Transfections of mammalian cells and analysis of transgene expression

Transfections of empty vector, wild-type *ERBB2*, *BRF2* and *DSN1*, and I767M mutant *ERBB2* were performed essentially as previously described [45] (Additional file 19). The expression of transgenes in stable clones for *DSN1* and *BRF2* was evaluated at the mRNA level by qualitative and quantitative RT-PCR (Additional files 7 and 19), given that antibodies producing satisfactory western blot results were not available. The expression of wild-type and I767M mutant HER2 proteins was confirmed by western blot (see below). The expression of transgenes from pCMV-ZsGreen/TagRFP-derived plasmids was visually evaluated 48 hours after transfection using a Nikon Eclipse Ti fluorescence microscope.

### Confocal microscopy for BRF2 and DSN1 subcellular localization

Cells expressing BRF2-ZsGreen and DSN1-ZsGreen, TagRFP and ZsGreen (control) proteins grown on coverslips were fixed for 15 minutes in 10% buffered formalin, washed with 1× phosphate buffered saline (PBS), counterstained with 300 nM 4',6-diamidino-2-phenylindole (DAPI; Life Technologies) for 2 minutes, and mounted using ProLong Gold Antifade Reagent (LifeTechnologies). After 24 hours, fluorescence images were acquired using a Leica TCS SP5-II Upright microscope.

### Growth curves

T47D and MCF7 cells stably expressing HER2 wild-type, HER2(I767M) and vector control (T47D, 1,000 cells/well and MCF7, 500 cells/well) were seeded in the corresponding normal growth medium in 96-well plates in triplicate as previously described [45] (Additional file 19). Growth curves were plotted and analyzed (multiple t-tests, corrected for multiple comparisons using the Holm-Šídák method, alpha: 0.05) using GraphPad Prism v_6.0c (GraphPad Software, Inc., La Jolla, CA, USA).

### Protein fractionation

Cytoplasmic and membrane/organellular enriched protein fractions from MCF7 and T47D cells expressing HER2 wild-type and HER2(I767M) proteins and vector control were prepared using a Cell Fractionation Kit (Cell Signaling Technologies, Danvers, MA, USA) following the manufacturer’s protocol. To determine the efficiency and purity of the cell fractionation, the separated subcellular fractions were assayed by western blotting using antibodies against MEK1/2 (cytoplasm) and AIF (membrane/organellular) (Cell Fractionation Antibody Sampler Kit, Cell Signaling Technologies), and against HER2 as previously described [45].

### Western blotting

Standard western blotting was conducted as previously described [63]. Antibodies and dilutions are described in Additional file 19. Quantification of conjugated secondary antibodies and analysis were performed using the Image Studio Software from LI-COR (LI-COR Biosciences, Lincoln, NE, USA).

### Foci formation

NIH3T3 cells expressing HER2 wild-type, HER2(I767M), BRF2 and DSN1 protein, and empty vector control cells were seeded at 5 × 10^5^ cells density in six-well culture dishes in full media without penicillin/ streptomycin for up to 21 days, then fixed with methanol and stained with 0.5% (w/v) crystal violet. Photomicrographs were taken using a Nikon DS5000 digital camera at day 21 for empty vector, BRF2 and DSN1, and using the EVOS XL Imaging System (Life Technologies) at days 5 and 12 for HER2 wild-type, HER2(I767M) and empty vector. All experiments were performed in triplicate. Foci were counted at the end of the assays using Image J and analyzed using GraphPad Prism v_6.0c (unpaired Student’s *t*-test, two-tailed).

### Anchorage-independent growth

Anchorage-independent transformation assays were performed using the Cell Biolabs CytoSelect 96-well Cell Transformation Assay (colorimetric, Cell Biolabs, San Diego, CA, USA) following the manufacturer’s instructions. Briefly, MCF10A cells stably expressing HER2 wild-type, HER2(I767M), BRF2 and DSN1 proteins, as well as vector control cells, were incubated in a proprietary semisolid agar media for 8 days before being solubilized, transferred and detected by the provided MTT Solution (570 nm) using the Victor X4 Multimode Plate Reader (PerkinElmer, Waltham, MA, USA). Assays were performed in quadruplicate reactions. For colony counting, soft-agar cultures were set up in triplicate as described above for i) MCF10A cells (stable for HER2 wild-type, HER2(I767M), BRF2, DSN1 and empty vector; transient for HER2 wild-type, HER2(I767M), HER2(Y835F), HER2(V777L) and empty vector), ii) NIH3T3 cells (stable for HER2 wild-type, HER2(I767M) and empty vector; transient for HER2 wild-type, HER2(I767M), HER2(Y835F), HER2(V777L) and empty vector), and iii) MCF12A cells (transient for HER2 wild-type, HER2(I767M), HER2(Y835F), HER2(V777L) and empty vector). Colony number and size were documented at day 9 using the phase contrast EVOS XL Imaging System (Life Technologies). Colonies were counted in Image J and colony size was determined in MetaMorph Image Analysis software (Molecular Devices, Sunnyvale, CA, USA). Analyses were carried out using GraphPad Prism v_6.0c.

### ERBB2-TagRFP and ERBB2(I767M)-TagRFP immunoprecipitation and tyrosine kinase assay

Forty-eight hours post-transfection with pCMV6-*ERBB2*::*TagRFP*, pCMV6-*ERBB2*(*I767M*)::*TagRFP* and the vector control, HEK293T cells were treated with 10 ng/ml of human neuregulin-1 (hNRG-1, Cell Signaling Technologies) for HER2 pre-activation or vehicle (20 mM citrate, pH 3.0) for 15 minutes. TagRFP antibody (Evrogen, Farmingdale, NY, USA) was crosslinked to magnetic beads using the PierceTM Crosslink Magnetic IP/Co-IP Kit (Thermo Scientific, Somerset, NJ, USA) following the manufacturers’ recommendations. On the day of preparation, 600 μl of protein lysates at 1 mg/ml were pre-cleared using 75 μl of washed protein A/G magnetic bead slurry and incubated for 1 hour at 4°C. Triplicates of 200 μl (1 mg/ml) of the pre-cleared lysates were then incubated with the equivalent of 5 μg of TagRFP antibody conjugated to magnetic beads under gentle rocking overnight at 4°C. The magnetic beads were then pelleted by placing the tubes in a magnetic separation rack. The magnetic bead pellets were washed five times with 1 ml of ice-cold M-PER Mammalian Protein Extraction Reagent supplemented with Halt Protease and Phosphatase inhibitors cocktail (Thermo Scientific). All steps were carried out at 4°C. Tyrosine kinase activity was evaluated using the ADP Hunter HS Assay (DiscoveRx, Fremont, CA, USA) and the Tyrosine Kinase Assay Kit (colorimetric detection, Millipore, Billerica, MA, USA) essentially as previously described [45] (Additional file 19).

### Three-dimensional matrigel cultures

MCF10A and MCF12A cells expressing HER2 wild-type, HER2(I767M), BRF2 and DSN1 proteins, as well as vector control cells, were seeded on top of growth factor-reduced reconstituted basement membrane (Matrigel, BD Biosciences, San Jose, CA, USA) and analyzed essentially as previously described [45] (Additional file 19).

### Data availability

aCGH data have been deposited into the NCBI Gene Expression Omnibus under the accession GSE67908. The R code for analysis of the aCGH data is deposited on GitHub [64]. Whole exome data have been deposited into the NCBI Sequence Read Archive under the accession SRP049005.

## Additional files

Additional file 1:
**Clinico-pathologic characteristics of HER2 heterogeneous breast cancers included in this study, and genomic analyses performed.**
Additional file 2:
**Representative micrographs of HER2 heterogeneous breast cancers included in this study.** Micrographs of representative hematoxylin and eosin (H&E) stained sections and HER2 immunohistochemistry (IHC) of 12 HER2 heterogeneous breast cancers included in this study (T1-T13). Microdissected HER2-positive and HER2-negative components of each case were subjected to gene copy number profiling, and chromosome 17 plots are shown to confirm the presence (arrow) and absence of *HER2* gene amplification (17q12), respectively. In the chromosome plots, the circular binary segmentation (cbs)-smoothed Log_2_ ratios for each bacterial artificial chromosome mapping to chromosome 17 were plotted on the y-axis and their genomic positions were plotted on the x-axis. Gains, amplifications and losses are highlighted in dark green, bright green and red, respectively. Additional file 3:
**Differences in the patterns of gene copy number alterations between the HER2-positive and HER2-negative components of HER2 heterogeneous breast cancers.** Subtraction of gene copy number alterations identified in the *HER2* non-amplified component from those in the *HER2* amplified component of a given HER2 heterogeneous breast cancer. Differences between the genomic profiles were found to be negligible (for example, T1, top), moderate (for example, T10, middle) or substantial (for example, T12, bottom). In these genome plots, the scaled and median centered circular binary segmentation (cbs)-smoothed Log_2_ ratios for each bacterial artificial chromosome (BAC) obtained in the analysis of the HER2-negative component was subtracted from the respective BAC from the HER2-positive component and plotted on the y-axis according to its genomic position on the x-axis. Gains are highlighted in green, and losses are depicted in red. Additional file 4:
**List of amplifications present in HER2-negative but absent in matched HER2-positive components of HER2 heterogeneous breast cancers.**
Additional file 5:
**Amplified genes restricted to HER2-negative components of HER2 heterogeneous breast cancers, whose expression is copy number-regulated in luminal breast cancers of the TCGA dataset.**
Additional file 6:
**Amplified genes restricted to HER2-negative components of HER2 heterogeneous breast cancers, and their copy number profiles in luminal breast cancers from The Cancer Genome Atlas (TCGA) dataset.** **(A)** Re-analysis of gene copy number profiles of luminal breast cancers from the TCGA dataset using amplified genes identified to be restricted to HER2-negative components of HER2 heterogeneous breast cancers. Gene copy number information was retrieved from the cBioPortal website [65]. **(B)** *HER2*, *BRF2* and *DSN1* are copy number regulated genes. Correlation between mRNA expression (y-axis) and copy number states (x-axis) as determined by GISTIC, retrieved from the cBioPortal website [65]. Additional file 7:
**HER2, BRF2 and DSN1 mRNA expression levels.** **(A,B)** Given the lack of commercial anti-BRF2 and anti-DSN1 antibodies that produced reliable western blot results, the mRNA expression levels of forced expression of BRF2 and DSN1 in MCF10A and NIH3T3 cells were assessed by semiquantitative RT-PCR (A) and by quantitative real-time RT-PCR (B). Wild-type HER2 (WT) and I767M mutant HER2 (MUT), as well as GAPDH were included as controls. Additional file 8:
**Sequencing statistics of the HER2 heterogeneous breast cancer subjected to whole exome sequencing and to targeted capture massively parallel sequencing using a platform containing baits targeting all exons of 273 genes.**
Additional file 9:
**Mutations identified by whole exome sequencing analysis and validated by amplicon sequencing on an Ion Torrent Personal Genome Machine or by targeted capture massively parallel sequencing on an Illumina HiSeq2000.**
Additional file 10:
**Mutations validated in the HER2-positive and HER2-negative components of case T12 using targeted capture massively parallel sequencing.** Allelic fractions of mutations identified in HER2-positive and HER2-negative components of the HER2 heterogeneous breast cancer T12 subjected to targeted capture massively parallel sequencing using a panel of 273 genes comprising genes frequently mutated in breast cancer and DNA repair-related genes. Indel, insertion and deletion; SNV, single nucleotide variant. Additional file 11:
**Somatic mutations restricted to HER2-negative components of HER2 heterogeneous breast cancers identified by massively parallel sequencing.**
Additional file 12:
**Anchorage-independent growth of NIH3T3 cells stably expressing empty vector, wild-type and I767M mutant HER2.** Anchorage-independent growth of NIH3T3 cells stably expressing empty vector, wild-type HER2 or I767M mutant HER2 protein. The number and size of colonies was quantified (right). **P* < 0.05, ***P* < 0.01, unpaired t-test. Error bars represent standard deviation of mean. N.s., not significant. Additional file 13:
**Anchorage-independent growth of NIH3T3, MCF10A and MCF12A cells transiently expressing empty vector, HER2 wild-type, I767M mutant HER2, and other previously validated HER2 mutations.** **(A-C)** Anchorage-independent growth of NIH3T3 (A), MCF10A (B) and MCF12A (C) cells transiently expressing empty vector, wild-type HER2, HER2 I767M, HER2 V777L and HER2 Y835F in NIH3T3. The V777L and the Y835F HER2 mutations have been previously shown [7] to be strongly activating or not activating, respectively. The number and size of colonies was quantified (right). **P* < 0.05, ***P* < 0.01, ****P* < 0.001, *****P* < 0.0001, unpaired t-test. Error bars represent standard deviation of mean. N.s., not significant. Additional file 14:
**Cellular fractionation of MCF7 and T47D cells expressing wild-type and I767M mutant HER2.** Cellular fractionation and western blot analysis illustrating the subcellular distribution of forced expression of wild-type HER2 and I767M mutant HER2 in MCF7 and T47D, and the *HER2* amplified BT474 control cells (untransfected). HER2 expression was assessed by western blotting. The efficiency and purity of cellular fractionation was evaluated using expression of AIF (membrane/organellular localization) and MEK1/2 (cytoplasmic localization). Cyto, cytoplasm; Mem, membrane; WCL, whole cell lysate; WT, wild-type. Additional file 15:
**Assessment of neuregulin-1 expression in T47D and MCF7 cells.** **(A)** Neuregulin-1 expression was assessed in protein lysates and in conditioned media from MCF7 and T47D cells stably expressing empty vector, HER2 wild-type and I767M mutant HER2 using quantitative infrared fluorescent western blotting (LI-COR). **(B)** Quantitative RT-PCR analysis of *NRG1* mRNA expression in MCF7 and T47D cells. Additional file 16:
**Representative micrographs obtained from MCF12A cells transiently expressing I767M mutant HER2.** Note that some acinar structures display infiltrating borders. Original magnification, 40×. Additional file 17:
**Contralateral axillary relapse of case T4 after adjuvant trastuzumab and chemotherapy treatment.** **(A,B)** Representative micrographs of the hematoxylin and eosin stained sections of the distant relapse of case T4. Note the areas of necrosis and hemorrhage in the bottom left corner of (B). **(C,D)** Representative micrographs of HER2 immunohistochemical assessment of the distant relapse of case T4 demonstrate complete, weak-to-moderate membranous staining in >10% of the cells. **(E)** Chromogenic *in situ* hybridization confirmed the presence of *HER2* amplification (*HER2* probe, green; chromosome 17 centromere probe, red). **(F)** Chromosome 17 plot demonstrating amplification of the *HER2* locus. In the chromosome plot, the circular binary segmentation (cbs)-smoothed Log_2_ ratios for each bacterial artificial chromosome mapping to chromosome 17 are plotted on the y-axis and their genomic positions are plotted on the x-axis. Gains, amplifications and losses are highlighted in dark green, bright green and red, respectively. **(G)** Genome plot of the distant contra-lateral axillary relapse of case T4. In the genome plot, the cbs-smoothed Log_2_ ratios for each bacterial artificial chromosome are plotted on the y-axis and their genomic positions are plotted on the x-axis. Gains, amplifications and losses are highlighted in dark green, bright green and red, respectively. Note the similarities between the genome plot of the relapse and the HER2-positive component of the T4 primary tumor illustrated in Figure 3A. Original magnification: 40× (A,C); 100× (B,D); 200× (E); 400× (E, inset). Additional file 18:
**Cutaneous chest wall distant relapse of case T8 after adjuvant trastuzumab and chemotherapy treatment.** **(A,B)** Representative micrographs of the hematoxylin and eosin stained sections of the distant relapse of case T8. **(C,D)** Representative micrographs of HER2 immunohistochemical assessment of the distant relapse of case T8 demonstrate incomplete, weak-to-moderate membranous staining in >10% of the cells. **(E)** Chromogenic *in situ* hybridization confirmed the absence or presence of *HER2* amplification (*HER2* probe, black; chromosome 17 centromere probe, red). Original magnification: 40× (A,C); 100× (B,D); 200× (E). Additional file 19:
**Supplementary methods.**
Additional file 20:
**R script and code.**
Additional file 21:
**Sanger sequencing primers used in this study.**

## Abbreviations

aCGH: array-based comparative genomic hybridization
BAC: bacterial artificial chromosome
CISH: chromogenic *in situ* hybridization
CNA: copy number alteration
ER: estrogen receptor
FISH: fluorescence *in situ* hybridization
IHC: immunohistochemistry
PR: progesterone receptor
SNV: single nucleotide variant
TCGA: The Cancer Genome Atlas
