# Supplementary material for: Intra-tumor genetic heterogeneity and alternative driver genetic alterations in breast cancers with heterogeneous HER2 gene amplification
Source: Genome Biol. 2015 May 22;16(1):107. doi: 10.1186/s13059-015-0657-6 (PMC4440518; doi:10.1186/s13059-015-0657-6)
Supplement: Additional file 1: — Clinico-pathologic characteristics of HER2 heterogeneous breast cancers included in this study, and genomic analyses performed. [file 13059_2015_657_MOESM1_ESM.pdf]

Additional file 1. Clinico-pathologic characteristics of HER2 heterogeneous breast cancers included in this study, and genomic analyses performed.

	Overall tumor				HER2-positive component						HER2-negative component																		Genomic analysis performed		
Case ID	ER	PR	HER2	Grade	ER	PR	Grade	Ki67	St Gallen 2013 Subtype*	*TP53* status by Sanger sequencing	ER	PR	Grade	Ki67	St Gallen 2013 Subtype*	*TP53* status by Sanger sequencing	Age at diagnosis (years)	Tumor size (mm)	Lymph node status	Surgery	Radiation	Chemotherapy	Endocrine therapy	Trastuzumab	Follow-up (months)	Relapse	Death	aCGH	Exome Seq	Targeted Seq
T1	Positive	Positive	Positive	3	Positive (90%)	Positive (90%)	3	>14%	Luminal B-like	mutant (P152L)	Positive (90%)	Positive (90%)	3	>14%	Luminal B-like	mutant (P152L)	53	13	Negative (0/15)	Mastectomy	Yes	Taxotere, Avastin	Anastrozole	Yes	31	No	no	x		x
T2	Positive	Positive	Positive	2	Positive (80%)	Positive (70%)	2	≤14%	Luminal B-like	wild-type (P72R - SNP)	Positive (80%)	Positive (70%)	2	≤14%	Luminal A-like	wild-type (P72R - SNP)	44	10	Negative (0/7)	Breast conservation	Yes	Carboplatin, Taxotere, Avastin	Tamoxifen	Yes	13	No	no	x		
T3	Positive	Negative	Positive	3	Positive (1%)	Negative	3	>14%	Luminal B-like	mutant (E258D)	Positive (1%)	Negative	3	>14%	Luminal B-like	mutant (E258D)	44	27	Positive (6/10)	Breast conservation	Yes	FEC (neo-adjuvant), 5-FU + Vinorelbine	Tamoxifen	No	10	8 months	10 months	x		x
T4	Positive	Positive	Positive	3	Positive (60%)	Positive (5%)	3	>14%	Luminal B-like	wild-type	Positive (60%)	Positive (70%)	3	>14%	Luminal B-like	wild-type	42	50	Positive (3/10)	Mastectomy	Yes	FEC + Taxotere	Anastrozole	Yes (1 year)	102	5 years (contralateral axillary relapse, ER+ HER2+ by FISH)	No	x		x
T5	Positive	Negative	Positive	3	Positive (95%)	Negative	3	>14%	Luminal B-like	mutant (E286D)	Positive (95%)	Negative	3	>14%	Luminal B-like	mutant (E286D)	65	16	Negative (0/4)	Breast conservation	NA	NA	NA	NA	2	NA, lost to follow-up	NA, lost to follow-up	x		
T6	Positive	Positive	Positive	3	Positive (30%)	Negative	2	>14%	Luminal B-like	mutant (R273H)	Positive (80%)	Positive (20%)	3	>14%	Luminal B-like	mutant (R273H)	58	26	Positive (3/11)	Mastectomy	Yes	FEC + Taxol	Anastrozole	No	75	No	No	x	x	
T8	Positive	Negative	Positive	3	Negative	Negative	3	>14%	HER2 positive	wild-type	Positive (5%)	Negative	3	>14%	Luminal B-like	wild-type	61	20	Positive (2/14)	Mastectomy	Yes	FEC + Taxotere	No	Yes (1 year)	55	4 years (cutaneus relapse on chest wall, ER-, PR-, HER2-)	No	x		x
T9	Positive	Positive	Positive	1	Positive (100%)	Positive (10%)	1	≤14%	Luminal B-like	mutant (R282G)	Positive (100%)	Positive (10%)	1	≤14%	Luminal B-like	mutant (R282G)	69	30	Negative (NA)	Breast conservation	Yes	FEC + Taxotere	Letrozole, Exemestane, Tamoxifen	Yes (1 year)	65	No	No	x		x
T10	Positive	Negative	Positive	3	Positive (90%)	Negative	3	>14%	Luminal B-like	mutant (S94fs)	Positive (100%)	Negative	3	>14%	Luminal B-like	mutant (S94fs)	60	23	Positive (2/15)	Breast conservation	Yes	FEC + Taxotere	Anastrozole, Tamoxifen	Yes (1 year)	49	No	No	x		
T11	Positive	Positive	Positive	2	Positive (70%)	Positive (90%)	2	≤14%	Luminal B-like	mutant (G187_E192delLAPPQ)	Positive (70%)	Positive (100%)	2	≤14%	Luminal A-like	mutant (G187_E192delLAPPQ)	50	25	Negative (0/14)	Mastectomy	No	FEC + Taxotere	Tamoxifen, Letrozole	Yes (1 year)	44	No	No	x	x	
T12	Positive	Positive	Positive	3	Positive (90%)	Negative	3	>14%	Luminal B-like	mutant (T195N)	Positive (90%)	Positive (10%)	2	>14%	Luminal B-like	mutant (T195N)	76	15	Negative (0/15)	Mastectomy	No	Taxol	Letrozole	Yes (1 year)	56	No	No	x	x	x
T13	Positive	Positive	Positive	3	Negative	Negative	3	>14%	HER2 positive	mutant (S240I)	Positive (90%)	Positive (80%)	3	>14%	Luminal B-like	mutant (S240I)	50	16	Negative (0/21)	Mastectomy	No	AC	Exemestane	Yes (1 year)	42	No	No	x		

AC, cyclophosphamide, doxorubicin; ER, estrogen receptor; FEC, 5-fluorouracil, epirubicin, cyclophosphamide; NA, not available; PR, progesterone receptor.

*Goldhirsch et al, Ann Oncol 2013
