# Supplementary figures and images for: Intra-tumor genetic heterogeneity and alternative driver genetic alterations in breast cancers with heterogeneous HER2 gene amplification
Source: Genome Biol. 2015 May 22;16(1):107. doi: 10.1186/s13059-015-0657-6 (PMC4440518; doi:10.1186/s13059-015-0657-6)

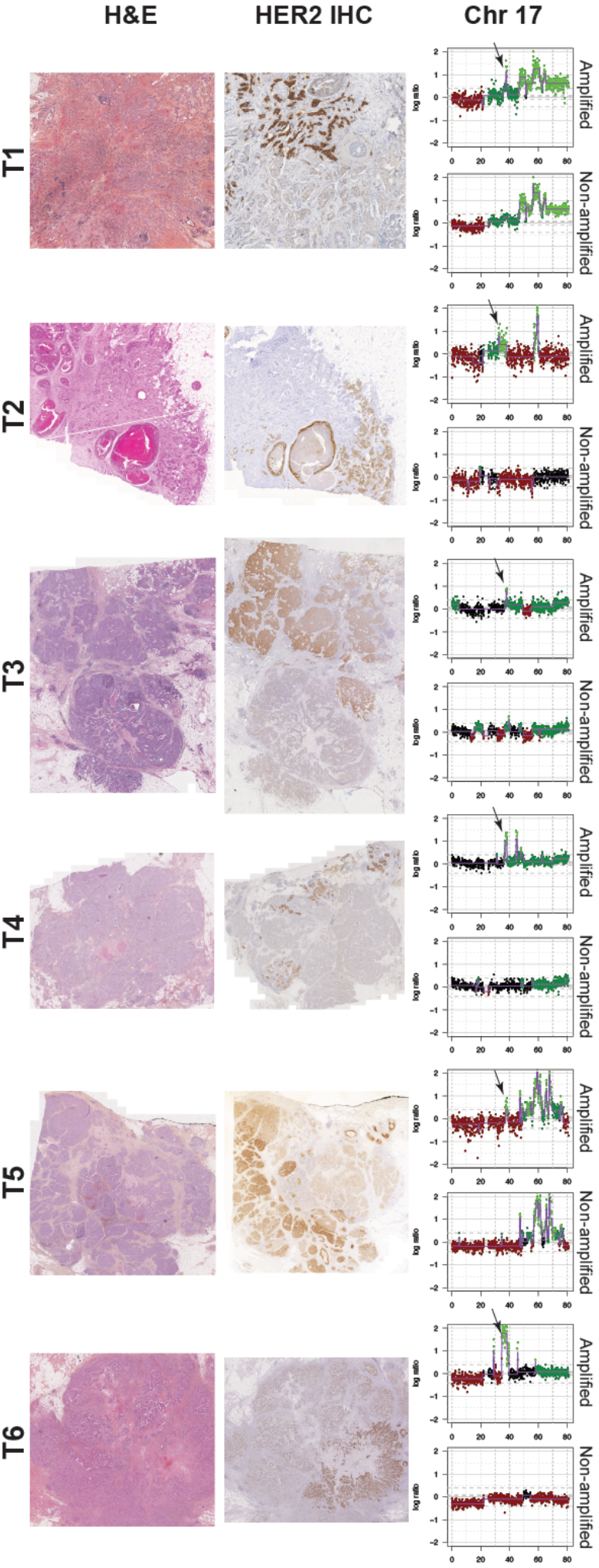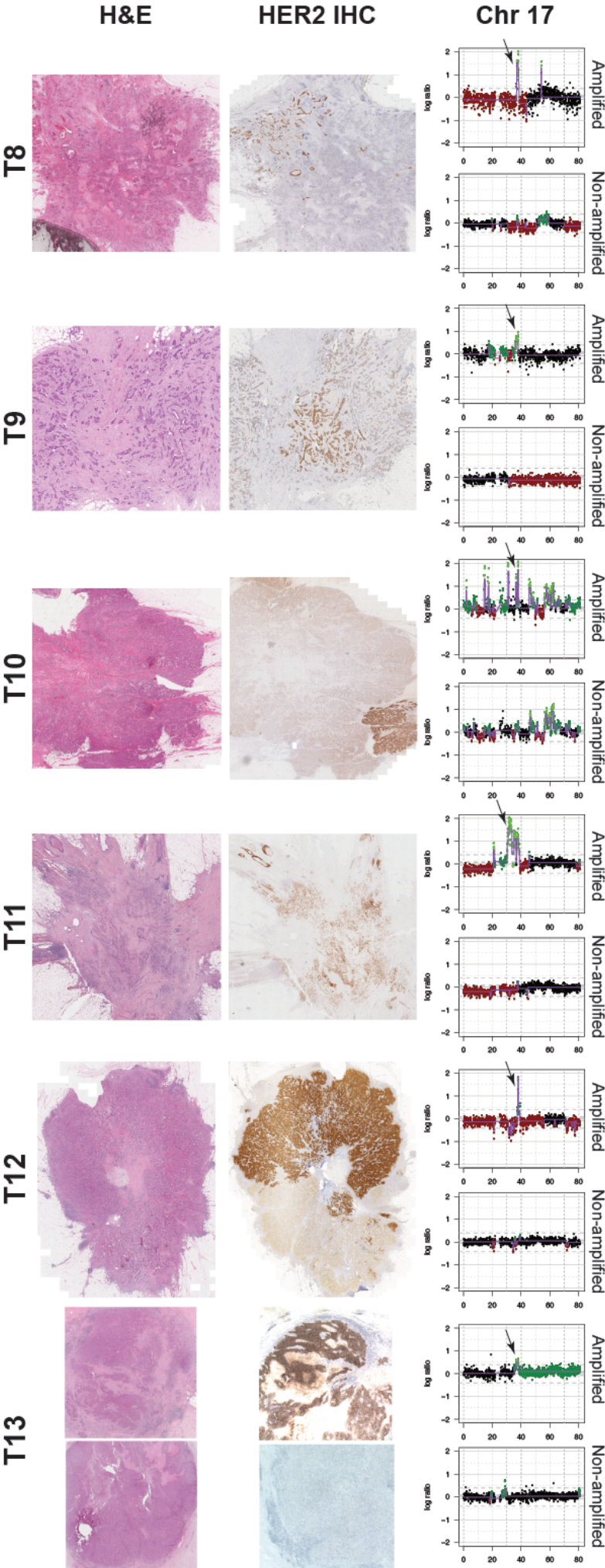

Supplement: Additional file 2: — Representative micrographs of HER2 heterogeneous breast cancers included in this study. Micrographs of representative hematoxylin and eosin (H&E) stained sections and HER2 immunohistochemistry (IHC) of 12 HER2 heterogeneous breast cancers included in this study (T1-T13). Microdissected HER2-positive and HER2-negative components of each case were subjected to gene copy number profiling, and chromosome 17 plots are shown to confirm the presence (arrow) and absence of HER2 gene amplification (17q12), respectively. In the chromosome plots, the circular binary segmentation (cbs)-smoothed Log2 ratios for each bacterial artificial chromosome mapping to chromosome 17 were plotted on the y-axis and their genomic positions were plotted on the x-axis. Gains, amplifications and losses are highlighted in dark green, bright green and red, respectively. [file 13059_2015_657_MOESM2_ESM.pdf]

# Additional file 3

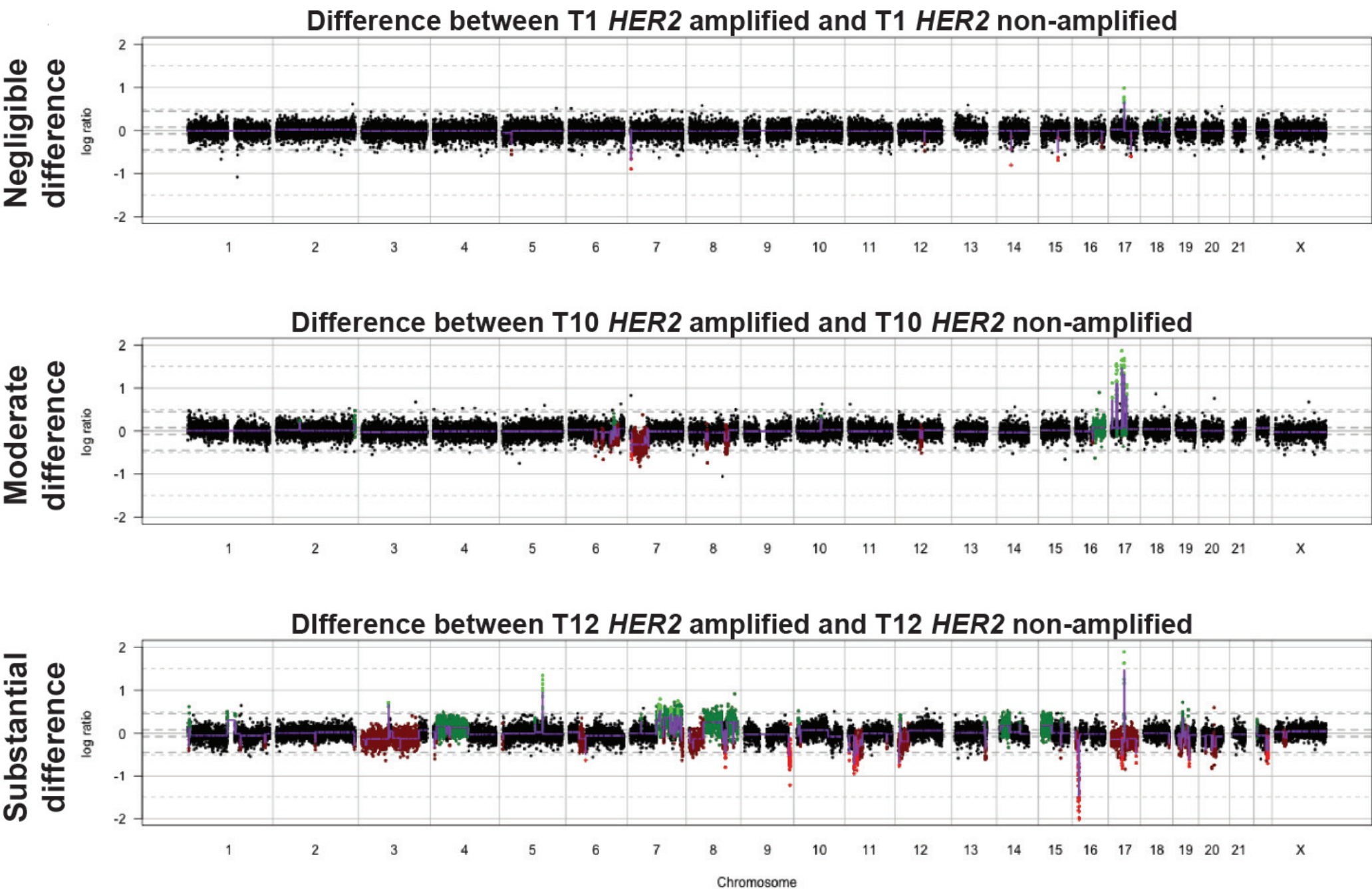

Supplement: Additional file 3: — Differences in the patterns of gene copy number alterations between the HER2-positive and HER2-negative components of HER2 heterogeneous breast cancers. Subtraction of gene copy number alterations identified in the HER2 non-amplified component from those in the HER2 amplified component of a given HER2 heterogeneous breast cancer. Differences between the genomic profiles were found to be negligible (for example, T1, top), moderate (for example, T10, middle) or substantial (for example, T12, bottom). In these genome plots, the scaled and median centered circular binary segmentation (cbs)-smoothed Log2 ratios for each bacterial artificial chromosome (BAC) obtained in the analysis of the HER2-negative component was subtracted from the respective BAC from the HER2-positive component and plotted on the y-axis according to its genomic position on the x-axis. Gains are highlighted in green, and losses are depicted in red. [file 13059_2015_657_MOESM3_ESM.pdf]

Additional file 6

A

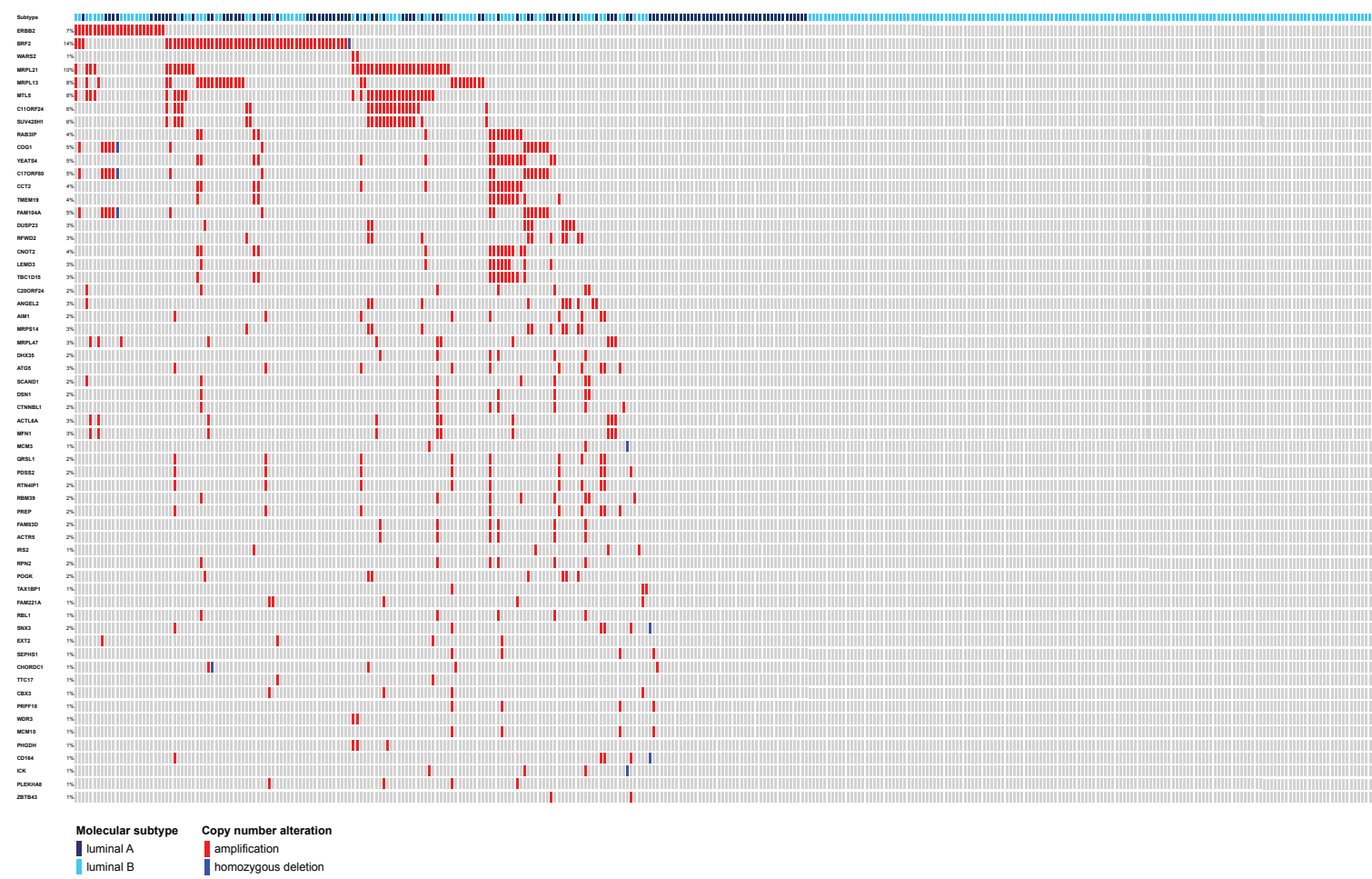

B

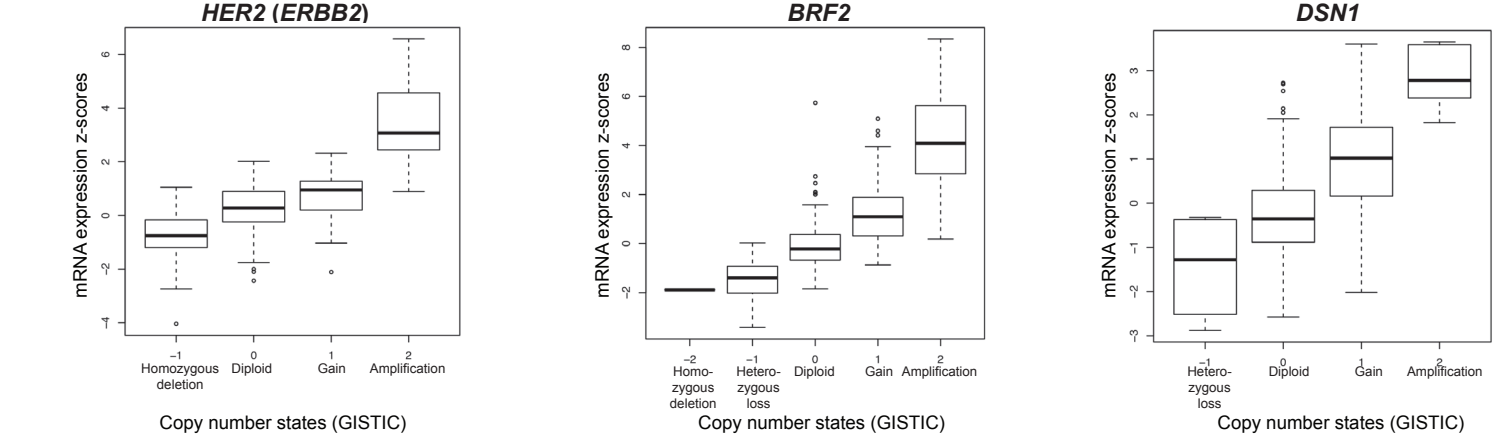

Supplement: Additional file 6: — Amplified genes restricted to HER2-negative components of HER2 heterogeneous breast cancers, and their copy number profiles in luminal breast cancers from The Cancer Genome Atlas (TCGA) dataset. (A) Re-analysis of gene copy number profiles of luminal breast cancers from the TCGA dataset using amplified genes identified to be restricted to HER2-negative components of HER2 heterogeneous breast cancers. Gene copy number information was retrieved from the cBioPortal website [65]. (B) HER2, BRF2 and DSN1 are copy number regulated genes. Correlation between mRNA expression (y-axis) and copy number states (x-axis) as determined by GISTIC, retrieved from the cBioPortal website [65]. [file 13059_2015_657_MOESM6_ESM.pdf]

Additional file 7  
A

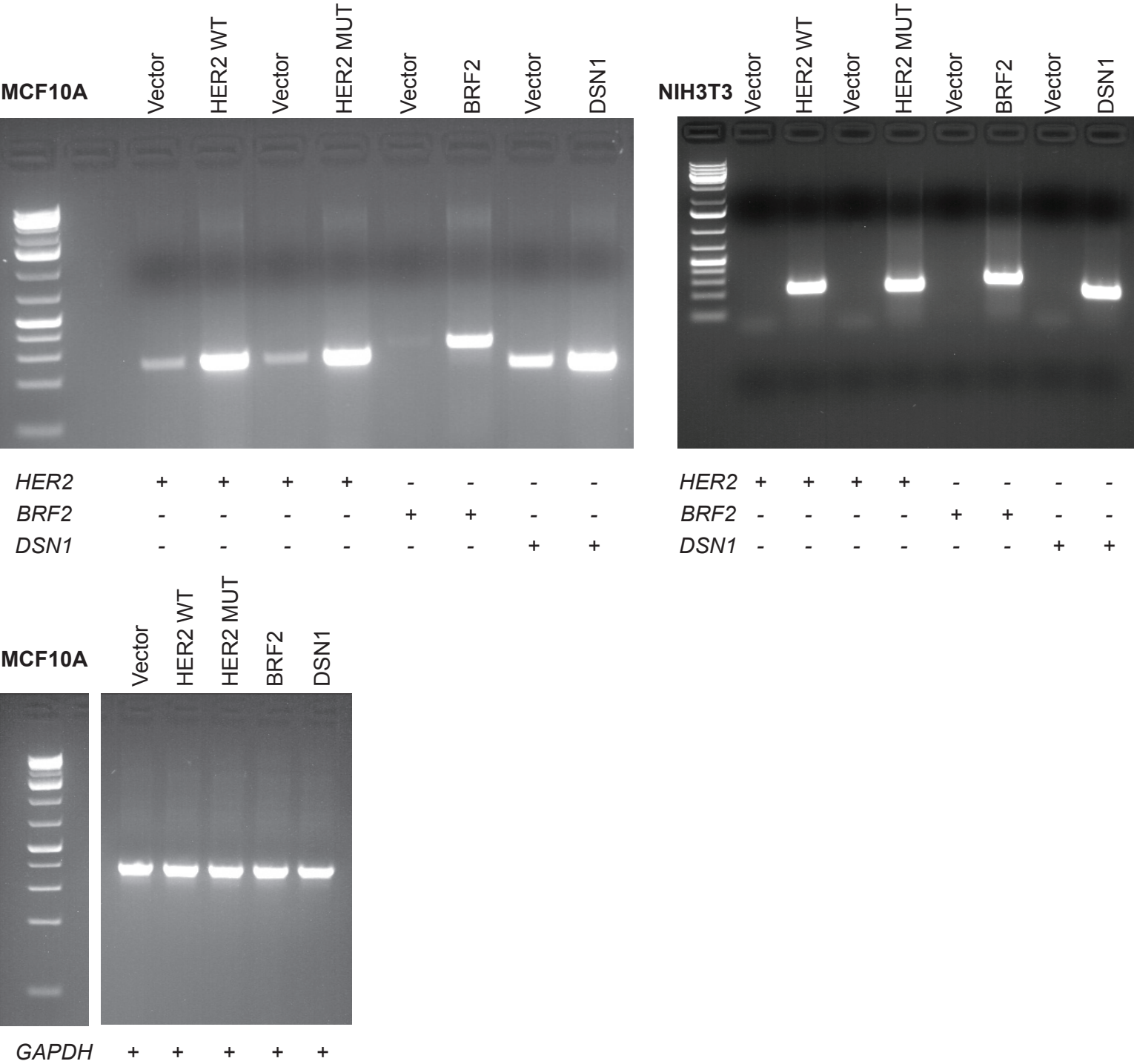

B

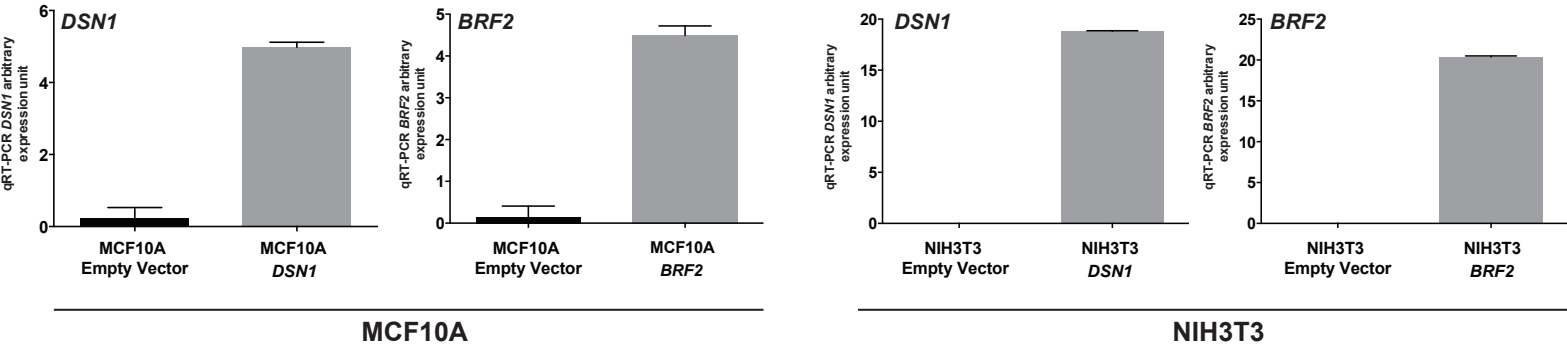

Supplement: Additional file 7: — HER2, BRF2 and DSN1 mRNA expression levels. (A,B) Given the lack of commercial anti-BRF2 and anti-DSN1 antibodies that produced reliable western blot results, the mRNA expression levels of forced expression of BRF2 and DSN1 in MCF10A and NIH3T3 cells were assessed by semiquantitative RT-PCR (A) and by quantitative real-time RT-PCR (B). Wild-type HER2 (WT) and I767M mutant HER2 (MUT), as well as GAPDH were included as controls. [file 13059_2015_657_MOESM7_ESM.pdf]

## Additional file 12

### NIH3T3 (stable)

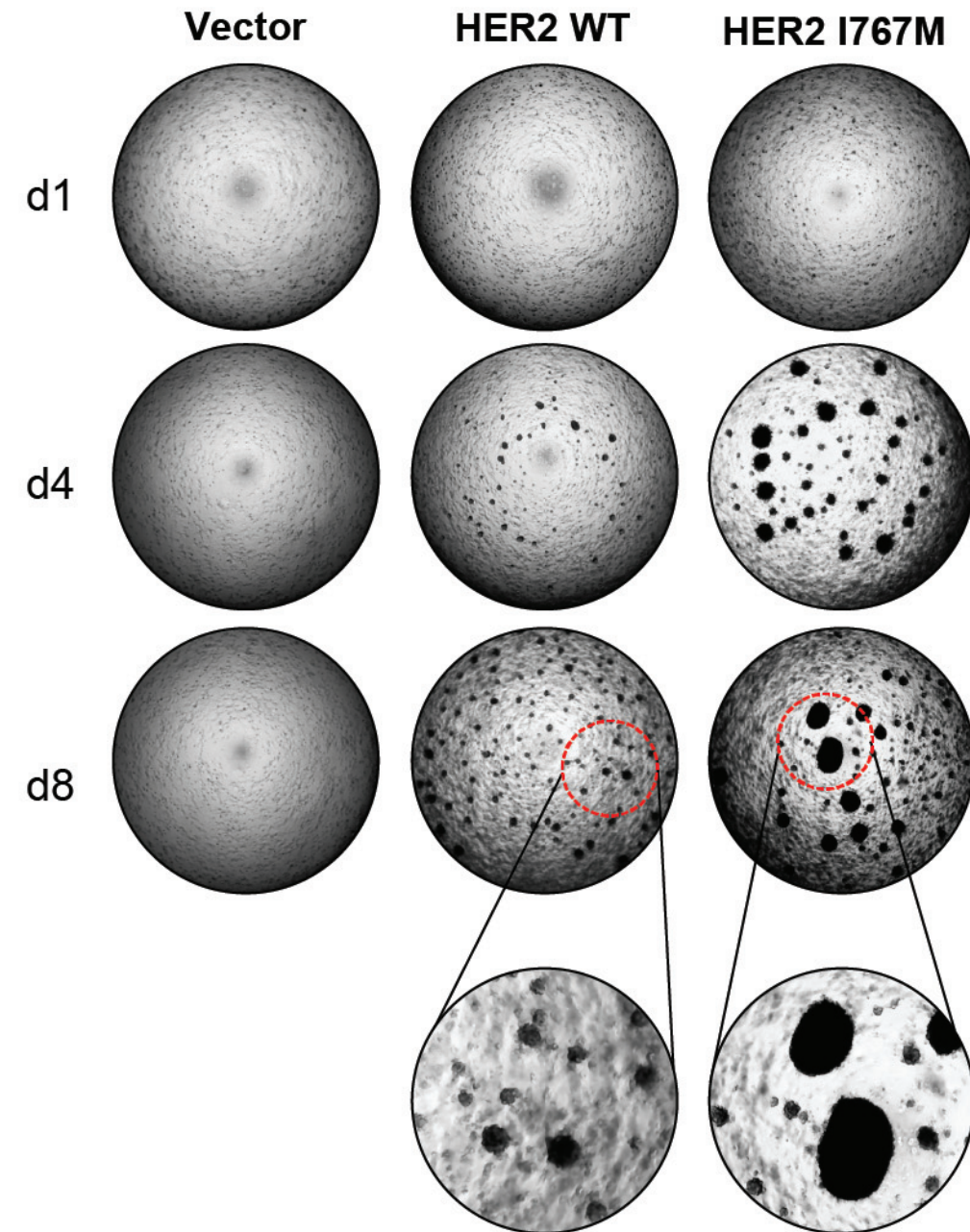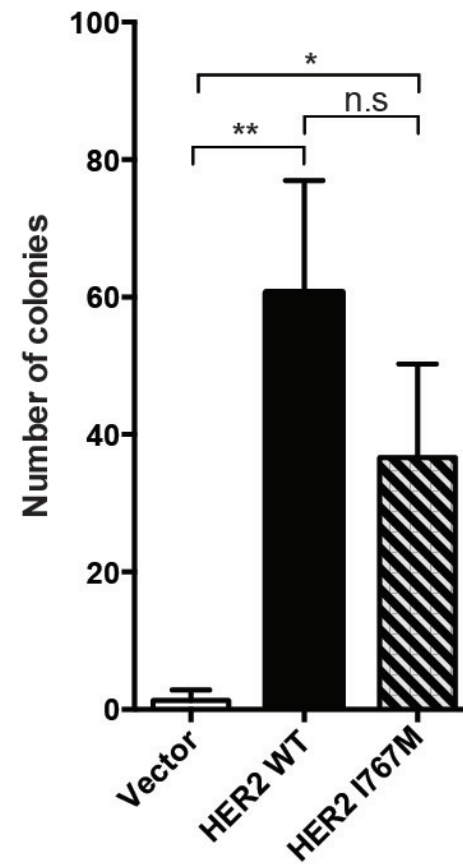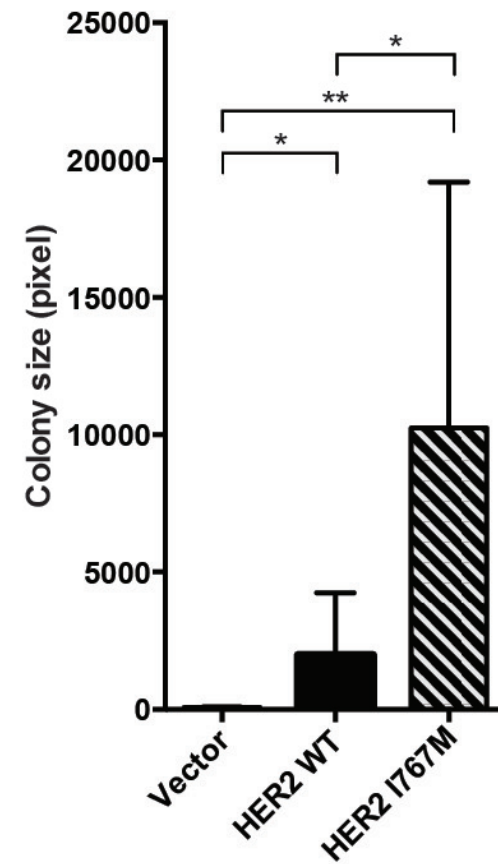

Supplement: Additional file 12: — Anchorage-independent growth of NIH3T3 cells stably expressing empty vector, wild-type and I767M mutant HER2. Anchorage-independent growth of NIH3T3 cells stably expressing empty vector, wild-type HER2 or I767M mutant HER2 protein. The number and size of colonies was quantified (right). *P < 0.05, **P < 0.01, unpaired t-test. Error bars represent standard deviation of mean. N.s., not significant. [file 13059_2015_657_MOESM12_ESM.pdf]

# Additional file 13

A

## NIH3T3 (transient)

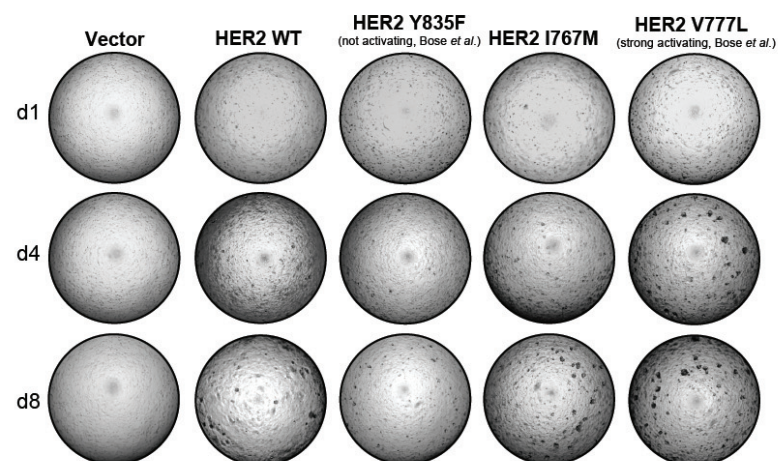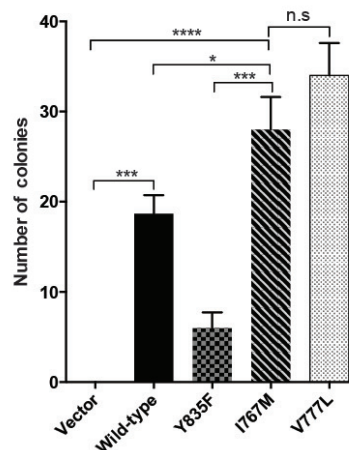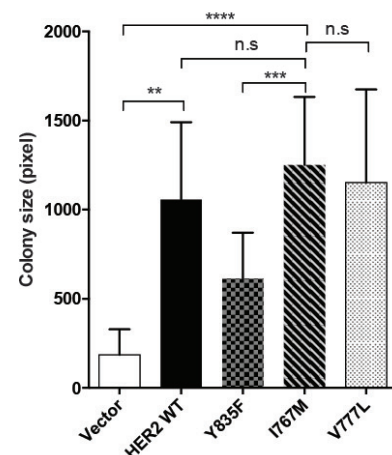

B

## MCF10A (transient)

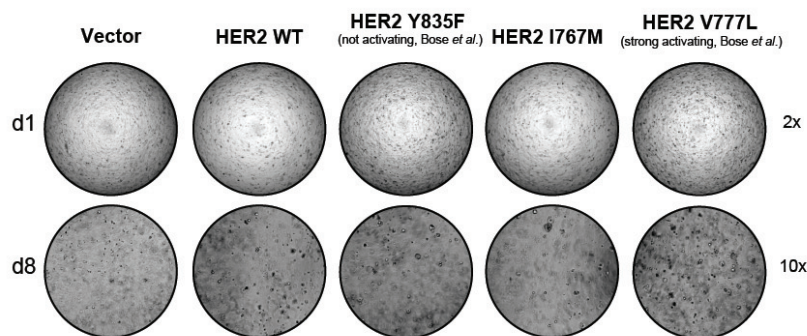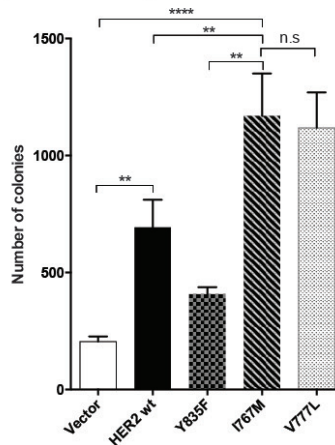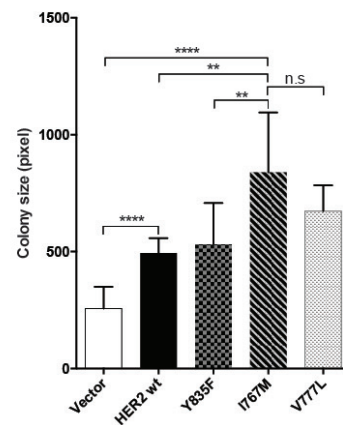

C

## MCF12A (transient)

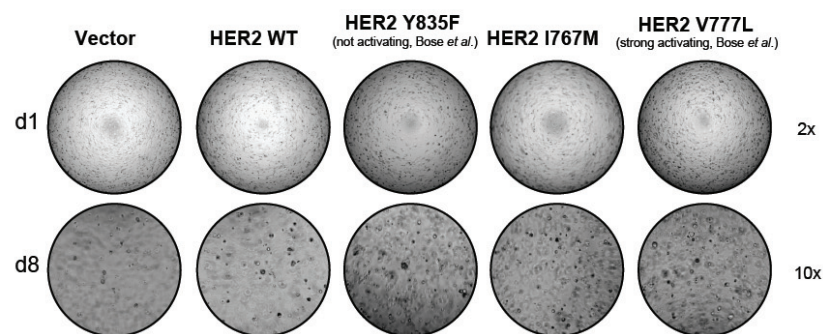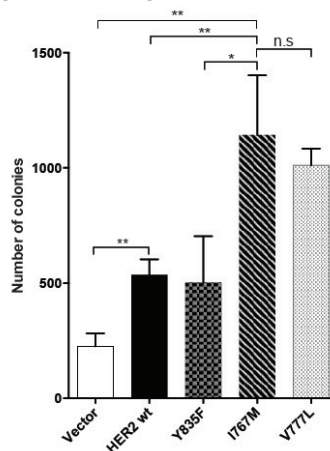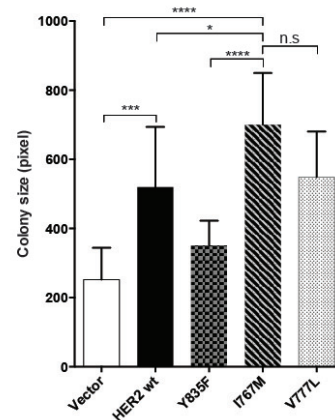

Supplement: Additional file 13: — Anchorage-independent growth of NIH3T3, MCF10A and MCF12A cells transiently expressing empty vector, HER2 wild-type, I767M mutant HER2, and other previously validated HER2 mutations. (A-C) Anchorage-independent growth of NIH3T3 (A), MCF10A (B) and MCF12A (C) cells transiently expressing empty vector, wild-type HER2, HER2 I767M, HER2 V777L and HER2 Y835F in NIH3T3. The V777L and the Y835F HER2 mutations have been previously shown [7] to be strongly activating or not activating, respectively. The number and size of colonies was quantified (right). *P < 0.05, **P < 0.01, ***P < 0.001, ****P < 0.0001, unpaired t-test. Error bars represent standard deviation of mean. N.s., not significant. [file 13059_2015_657_MOESM13_ESM.pdf]

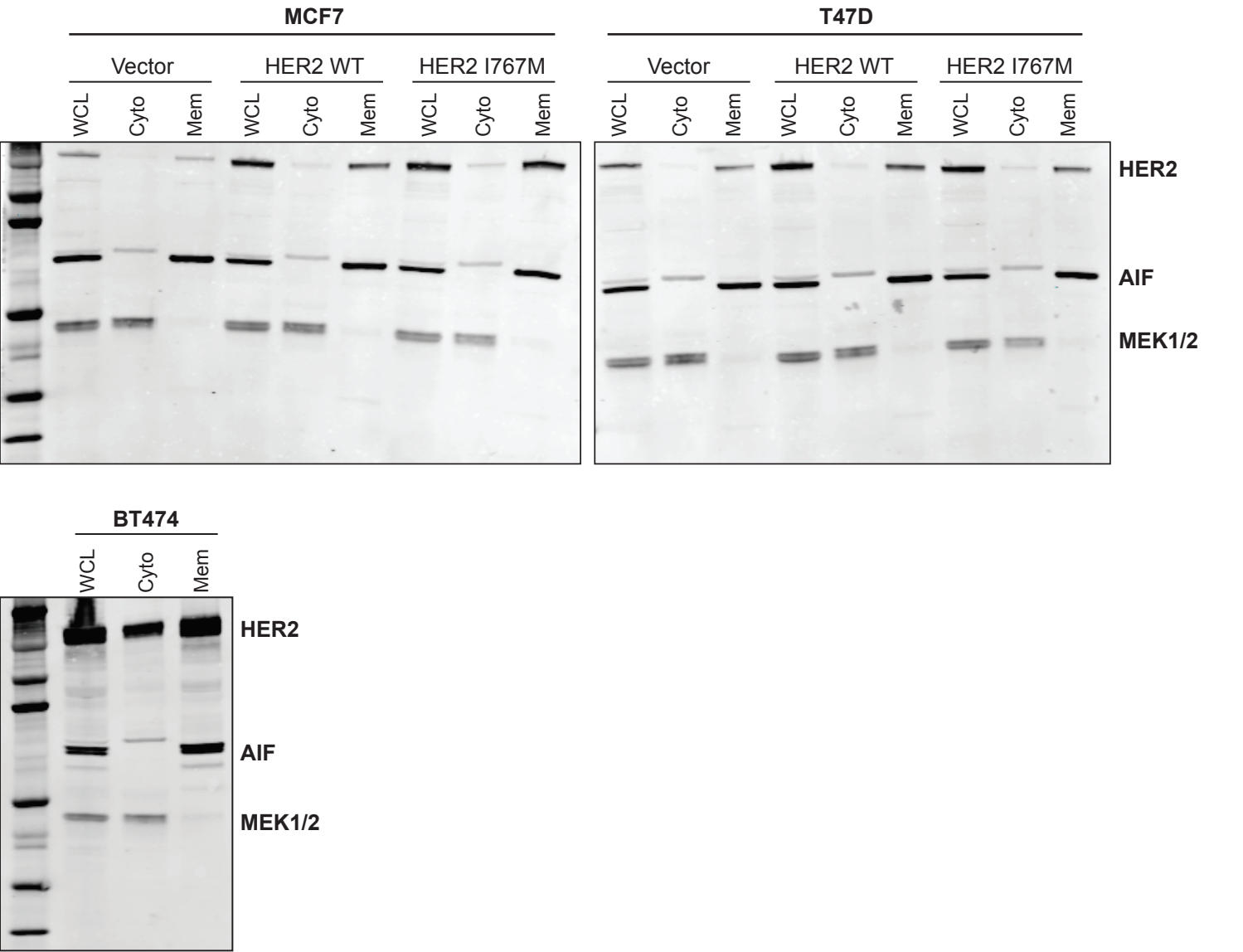

Supplement: Additional file 14: — Cellular fractionation of MCF7 and T47D cells expressing wild-type and I767M mutant HER2. Cellular fractionation and western blot analysis illustrating the subcellular distribution of forced expression of wild-type HER2 and I767M mutant HER2 in MCF7 and T47D, and the HER2 amplified BT474 control cells (untransfected). HER2 expression was assessed by western blotting. The efficiency and purity of cellular fractionation was evaluated using expression of AIF (membrane/organellular localization) and MEK1/2 (cytoplasmic localization). Cyto, cytoplasm; Mem, membrane; WCL, whole cell lysate; WT, wild-type. [file 13059_2015_657_MOESM14_ESM.pdf]

A

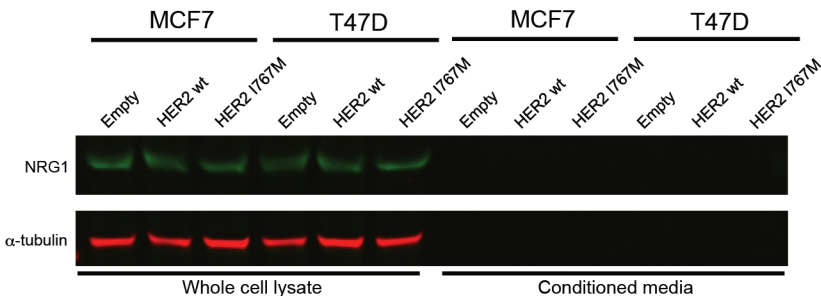

B

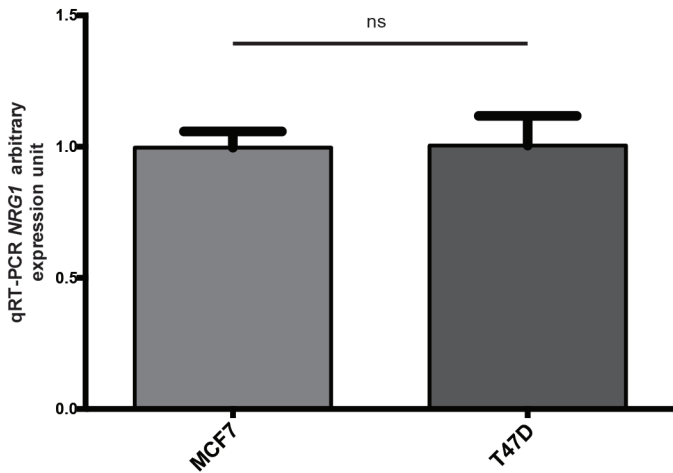

Supplement: Additional file 15: — Assessment of neuregulin-1 expression in T47D and MCF7 cells. (A) Neuregulin-1 expression was assessed in protein lysates and in conditioned media from MCF7 and T47D cells stably expressing empty vector, HER2 wild-type and I767M mutant HER2 using quantitative infrared fluorescent western blotting (LI-COR). (B) Quantitative RT-PCR analysis of NRG1 mRNA expression in MCF7 and T47D cells. [file 13059_2015_657_MOESM15_ESM.pdf]

# Additional file 16

MCF12A (HER2 I767M)

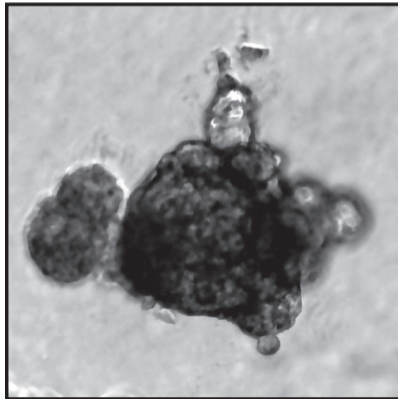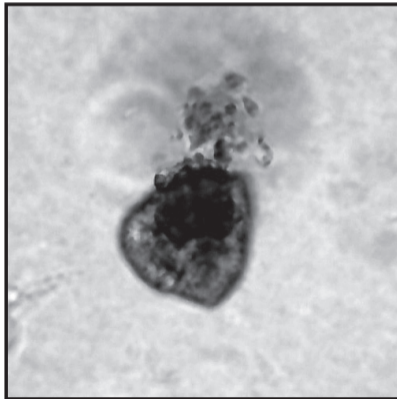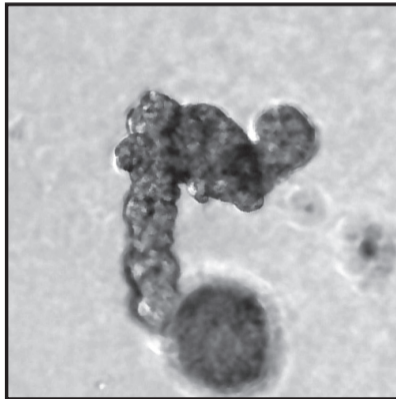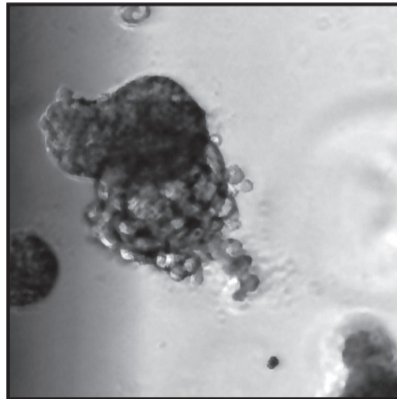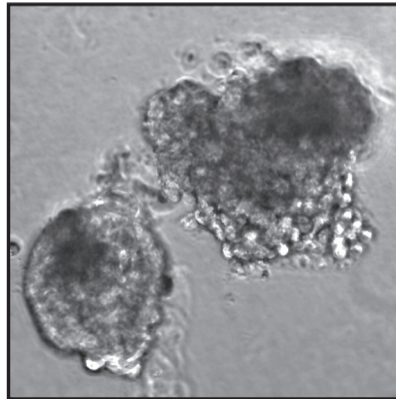

Supplement: Additional file 16: — Representative micrographs obtained from MCF12A cells transiently expressing I767M mutant HER2. Note that some acinar structures display infiltrating borders. Original magnification, 40×. [file 13059_2015_657_MOESM16_ESM.pdf]

# Additional file 17

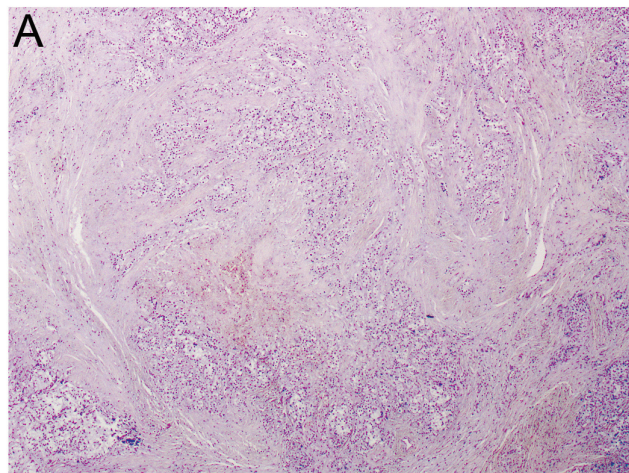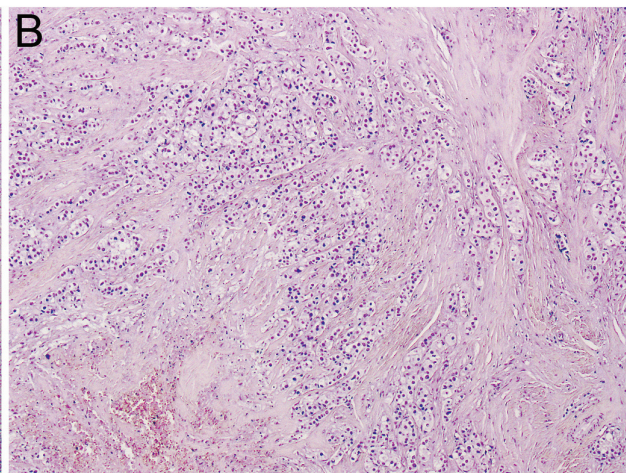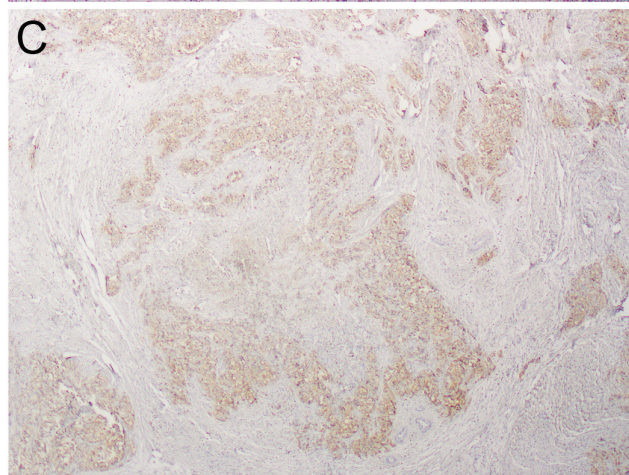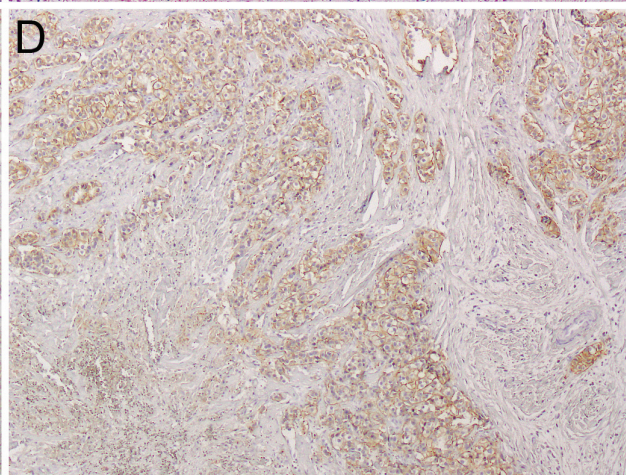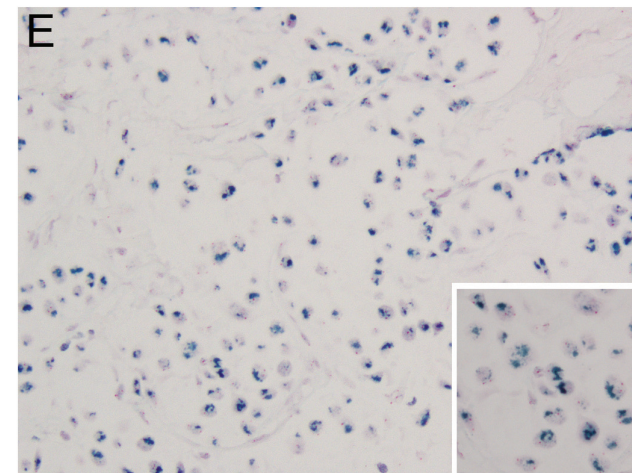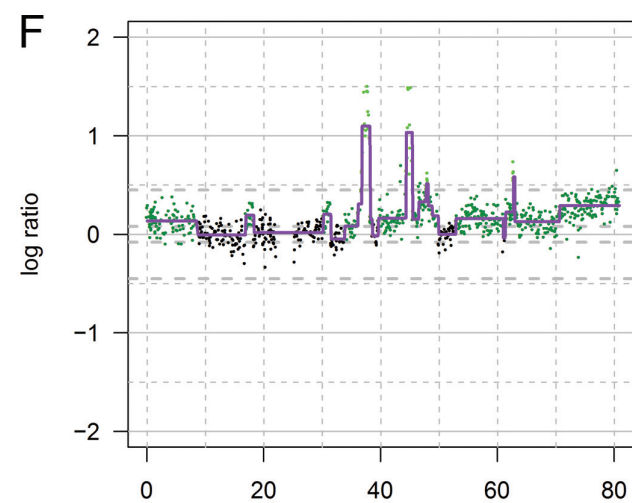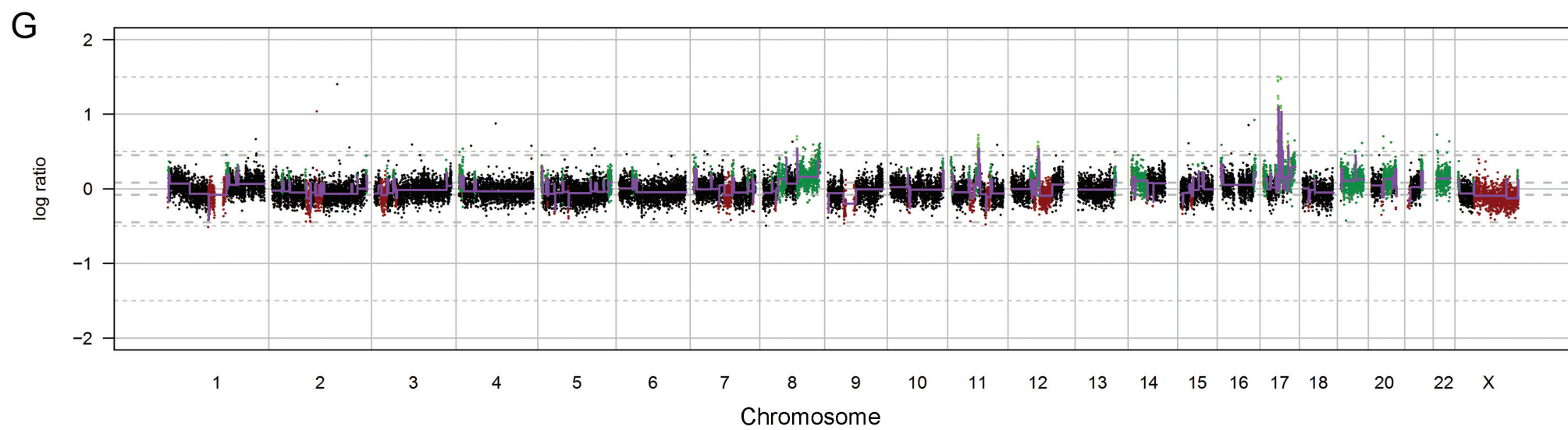

Supplement: Additional file 17: — Contralateral axillary relapse of case T4 after adjuvant trastuzumab and chemotherapy treatment. (A,B) Representative micrographs of the hematoxylin and eosin stained sections of the distant relapse of case T4. Note the areas of necrosis and hemorrhage in the bottom left corner of (B). (C,D) Representative micrographs of HER2 immunohistochemical assessment of the distant relapse of case T4 demonstrate complete, weak-to-moderate membranous staining in >10% of the cells. (E) Chromogenic in situ hybridization confirmed the presence of HER2 amplification (HER2 probe, green; chromosome 17 centromere probe, red). (F) Chromosome 17 plot demonstrating amplification of the HER2 locus. In the chromosome plot, the circular binary segmentation (cbs)-smoothed Log2 ratios for each bacterial artificial chromosome mapping to chromosome 17 are plotted on the y-axis and their genomic positions are plotted on the x-axis. Gains, amplifications and losses are highlighted in dark green, bright green and red, respectively. (G) Genome plot of the distant contra-lateral axillary relapse of case T4. In the genome plot, the cbs-smoothed Log2 ratios for each bacterial artificial chromosome are plotted on the y-axis and their genomic positions are plotted on the x-axis. Gains, amplifications and losses are highlighted in dark green, bright green and red, respectively. Note the similarities between the genome plot of the relapse and the HER2-positive component of the T4 primary tumor illustrated in Figure 3A. Original magnification: 40× (A,C); 100× (B,D); 200× (E); 400× (E, inset). [file 13059_2015_657_MOESM17_ESM.pdf]

Additional file 18

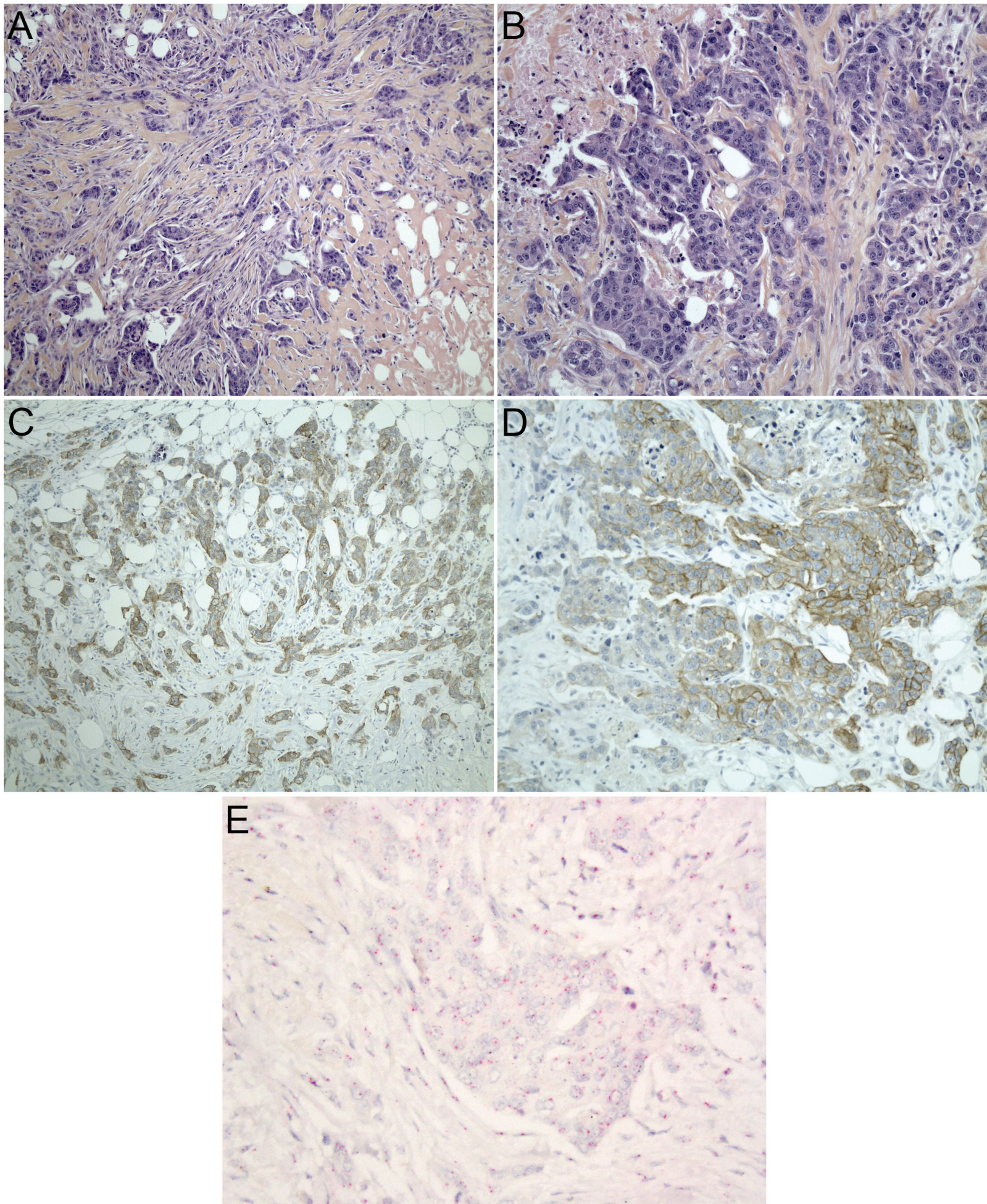

Supplement: Additional file 18: — Cutaneous chest wall distant relapse of case T8 after adjuvant trastuzumab and chemotherapy treatment. (A,B) Representative micrographs of the hematoxylin and eosin stained sections of the distant relapse of case T8. (C,D) Representative micrographs of HER2 immunohistochemical assessment of the distant relapse of case T8 demonstrate incomplete, weak-to-moderate membranous staining in >10% of the cells. (E) Chromogenic in situ hybridization confirmed the absence or presence of HER2 amplification (HER2 probe, black; chromosome 17 centromere probe, red). Original magnification: 40× (A,C); 100× (B,D); 200× (E). [file 13059_2015_657_MOESM18_ESM.pdf]
