# Supplementary material for: Intra-tumor genetic heterogeneity and alternative driver genetic alterations in breast cancers with heterogeneous HER2 gene amplification
Source: Genome Biol. 2015 May 22;16(1):107. doi: 10.1186/s13059-015-0657-6 (PMC4440518; doi:10.1186/s13059-015-0657-6)
Supplement: Additional file 5: — Amplified genes restricted to HER2-negative components of HER2 heterogeneous breast cancers, whose expression is copy number-regulated in luminal breast cancers of the TCGA dataset. [file 13059_2015_657_MOESM5_ESM.pdf]

Additional file 5: Amplified genes restricted to HER2-negative components of HER2 heterogeneous breast cancers, whose expression is copy number-regulated in luminal breast cancers of the TCGA dataset.

Tier	Gene	Case IDs (HER2-negative component)	T-test p-value between diploid and amplified	Adjusted p-value	Mean of amplified is at least mean+2SD of diploid	Mean of diploid is less than mean-2SD of amplified	Number of TCGA diploid cases with expression	Number of TCGA amplified cases with expression
1	BRF2	T2, T4	9.78E-21	7.32E-18	1	1	156	51
1	WARS2	T6	4.97E-20	2.48E-17	1	1	262	2
1	MRPL21	T6	2.01E-17	5.03E-15	1	1	220	38
1	MRPL13	T11	2.07E-13	2.22E-11	1	1	147	29
1	MTL5	T6	1.40E-12	9.51E-11	1	1	222	29
1	C11ORF24	T6	1.10E-09	4.12E-08	1	1	229	21
1	SUV420H1	T6	3.66E-09	1.28E-07	1	1	229	21
1	RAB3IP	T10	3.07E-08	8.37E-07	1	1	267	14
1	COG1	T5	3.93E-08	1.05E-06	1	1	209	16
1	YEATS4	T10	4.39E-08	1.13E-06	1	1	265	18
1	C17ORF80	T5	6.00E-08	1.50E-06	1	1	209	16
1	CCT2	T10	6.32E-08	1.54E-06	1	1	267	15
1	TMEM19	T10	6.39E-08	1.54E-06	1	1	270	13
1	FAM104A	T5	7.13E-08	1.64E-06	1	1	209	16
1	DUSP23	T12	4.41E-07	8.47E-06	1	1	112	10
1	RFWD2	T12	5.89E-07	1.10E-05	1	1	103	11
1	CNOT2	T10	1.58E-06	2.82E-05	1	1	266	14
1	LEMD3	T10	5.84E-06	9.12E-05	1	1	271	10
1	TBC1D15	T10	8.73E-06	0.00012947	1	1	270	12
1	C20ORF24	T12	1.02E-05	0.00014791	1	1	222	7
1	ANGEL2	T12	1.18E-05	0.00016711	1	1	101	11
1	AIM1	T13	1.28E-05	0.00017949	1	1	213	9
1	MRPS14	T12	1.80E-05	0.00023822	1	1	106	11
1	MRPL47	T13	1.89E-05	0.00024459	1	1	270	11
1	DHX35	T12	2.14E-05	0.00026884	1	1	220	6
1	ATG5	T13	2.42E-05	0.00029666	1	1	212	10
1	SCAND1	T12	3.10E-05	0.00035713	1	1	223	7
1	DSN1	T12	3.69E-05	0.00040381	1	1	222	6
1	CTNNBL1	T12	3.87E-05	0.0004205	1	1	217	7
1	ACTL6A	T13	4.00E-05	0.00042544	1	1	270	10
1	MFN1	T13	4.87E-05	0.00050304	1	1	269	10
1	MCM3	T12	4.95E-05	0.00050479	1	1	265	2
1	QRSL1	T13	5.35E-05	0.0005274	1	1	213	9
1	PDSS2	T13	5.75E-05	0.00056296	1	1	214	9
1	RTN4IP1	T13	7.55E-05	0.00071139	1	1	213	9
1	RBM39	T12	8.13E-05	0.00075187	1	1	222	8
1	PREP	T13	0.000109075	0.00099027	1	1	213	9
1	FAM83D	T12	0.000171451	0.00141117	1	1	219	6
1	ACTR5	T12	0.000276981	0.00203777	1	1	218	6
1	IRS2	T12	0.000296341	0.00216546	1	1	216	4
1	RPN2	T12	0.000317165	0.00230637	1	1	221	6
1	POGK	T12	0.000499793	0.00326939	1	1	109	7
1	TAX1BP1	T10	0.000708208	0.00440206	1	1	255	3
1	FAM221A	T10	0.000874557	0.00515782	1	1	260	5
1	RBL1	T12	0.00268525	0.01348902	1	1	221	5
1	SNX3	T13	0.0027986	0.01386775	1	1	220	5
1	EXT2	T9	0.003380655	0.01623148	1	1	265	4
1	SEPHS1	T5	0.00381579	0.01820399	1	1	274	4
1	CHORDC1	T6	0.003945072	0.01864264	1	1	187	4
1	TTC17	T9	0.004212032	0.01965615	1	1	264	2
1	CBX3	T10	0.004392522	0.02024615	1	1	258	4
1	PRPF18	T5	0.004476038	0.02050491	1	1	274	4
1	WDR3	T6	0.005655212	0.0249162	1	1	257	2
1	MCM10	T5	0.006140629	0.02658573	1	1	274	4
1	PHGDH	T6	0.006806959	0.02880459	1	1	259	3
1	CD164	T13	0.010481759	0.04121175	1	1	216	4
1	ICK	T12	0.010967817	0.04289762	1	1	265	3
1	PLEKHA8	T10	0.011820315	0.04599177	1	1	254	4
1	ZBTB43	T9	0.013071772	0.04982573	1	1	265	2
2	PROSC	T2, T4	2.98E-19	1.11E-16	1	0	156	51
2	LSM1	T2	1.43E-17	4.29E-15	1	0	152	46
2	RAB11FIP1	T2, T4	1.22E-16	2.61E-14	1	0	153	48
2	DDHD2	T2	2.21E-16	4.14E-14	1	0	151	45
2	TPCN2	T6	2.60E-16	4.32E-14	1	0	212	46
2	WHSC1L1	T2	3.92E-16	5.86E-14	1	0	156	46
2	ASH2L	T2	2.43E-15	3.31E-13	1	0	152	46
2	BAG4	T2	6.76E-15	8.44E-13	1	0	152	46
2	PPAPDC1B	T2	1.08E-14	1.24E-12	1	0	153	46
2	AZIN1	T3	6.27E-13	5.57E-11	1	0	152	31
2	KIAA0196	T11, T3	6.70E-13	5.57E-11	1	0	147	34
2	NDUFB9	T11, T3	5.96E-13	5.57E-11	1	0	147	33
2	YWHAZ	T3	6.47E-13	5.57E-11	1	0	151	33
2	IGHMBP2	T6	8.43E-13	6.01E-11	1	0	219	37
2	RRM2B	T3	8.04E-13	6.01E-11	1	0	153	32
2	SQLE	T11, T3	8.15E-13	6.01E-11	1	0	147	34
2	SLC25A32	T3	1.60E-12	1.04E-10	1	0	152	31
2	FAM84B	T11, T3	2.23E-12	1.39E-10	1	0	147	34
2	EIF4EBP1	T2	3.39E-12	2.03E-10	1	0	153	46
2	ATP6V1C1	T3	4.39E-12	2.53E-10	1	0	152	30
2	RAD21	T10, T11	1.31E-11	7.27E-10	1	0	147	33
2	ZNF706	T3	2.16E-11	1.16E-09	1	0	152	32
2	DERL1	T1, T3	2.25E-11	1.16E-09	1	0	147	30
2	TATDN1	T11, T3	4.98E-11	2.49E-09	1	0	147	33
2	FGFR1	T2	5.70E-11	2.75E-09	1	0	157	44
2	ERLIN2	T2, T4	7.70E-11	3.60E-09	1	0	156	51
2	BCAS3	T10	9.38E-11	4.26E-09	1	0	204	33
2	TRMT12	T11, T3	1.15E-10	5.08E-09	1	0	147	33
2	FAM91A1	T11, T3	1.36E-10	5.81E-09	1	0	147	31
2	C8ORF76	T1, T3	2.57E-10	1.07E-08	1	0	147	30
2	MED30	T11	4.36E-10	1.77E-08	1	0	148	33
2	SMIM19	T5	1.41E-09	5.16E-08	1	0	185	23
2	UBR5	T3	4.20E-09	1.43E-07	1	0	153	32
2	RNF139	T11, T3	4.84E-09	1.61E-07	1	0	147	33
2	MTBP	T11	5.63E-09	1.83E-07	1	0	147	29
2	ATAD2	T1, T3	8.26E-09	2.58E-07	1	0	147	31
2	POLB	T5	1.49E-08	4.48E-07	1	0	181	25
2	TAF2	T11	1.95E-08	5.74E-07	1	0	148	30
2	VDAC3	T5	2.10E-08	6.06E-07	1	0	182	25
2	SLC20A2	T5	2.80E-08	7.77E-07	1	0	183	24
2	TBC1D31	T1, T3	4.25E-08	1.12E-06	1	0	147	30
2	PHF20L1	T11	6.65E-08	1.58E-06	1	0	149	23
2	FAM49B	T11	6.85E-08	1.60E-06	1	0	150	24
2	HGSNAT	T5	7.26E-08	1.65E-06	1	0	187	21
2	IKBKB	T5	1.00E-07	2.24E-06	1	0	183	24
2	FNTA	T5	1.04E-07	2.29E-06	1	0	187	19
2	AP3M2	T5	1.39E-07	2.98E-06	1	0	182	21
2	ZBTB10	T6	1.53E-07	3.24E-06	1	0	160	23
2	MDM4	T1	3.46E-07	7.01E-06	1	0	93	20
2	CDC42EP4	T5	1.12E-06	2.02E-05	1	0	209	16
2	RNF170	T5	2.25E-06	3.87E-05	1	0	189	19
2	FAM83A	T1, T3	2.57E-06	4.33E-05	1	0	147	30
2	THAP1	T5	2.88E-06	4.79E-05	1	0	189	19
2	RAP1B	T10	3.14E-06	5.17E-05	1	0	268	15
2	CAND1	T10	6.09E-06	9.40E-05	1	0	268	13
2	RAB21	T10	8.03E-06	0.00012036	1	0	270	13
2	RBBP5	T1	9.40E-06	0.00013801	1	0	95	19
2	NIT1	T12	1.48E-05	0.00020156	1	0	109	14
2	RCOR3	T12	1.54E-05	0.0002075	1	0	103	12
2	DEDD	T12	1.84E-05	0.00024225	1	0	109	14
2	FRS2	T10	2.00E-05	0.00025548	1	0	266	16
2	RPS6KC1	T12	2.11E-05	0.00026828	1	0	101	11
2	MDM1	T10	2.81E-05	0.00033649	1	0	270	15
2	DYRK2	T10	3.31E-05	0.00036949	1	0	269	12
2	F11R	T12	3.30E-05	0.00036949	1	0	109	14
2	YTHDF1	T2	3.40E-05	0.00037722	1	0	206	12
2	CACYBP	T12	3.67E-05	0.00040381	1	0	106	11
2	B4GALT3	T12	4.00E-05	0.00042544	1	0	109	13
2	SLC2A4RG	T2	4.91E-05	0.0005038	1	0	205	11
2	AHCTF1	T3, T8	5.08E-05	0.00051092	1	0	109	11
2	PIGC	T12	5.06E-05	0.00051092	1	0	105	12
2	NDUFB5	T13	5.90E-05	0.00057013	1	0	270	11
2	PIK3CA	T13	6.61E-05	0.00062663	1	0	269	11
2	PIGM	T12	9.21E-05	0.00084149	1	0	111	10
2	ZNF639	T13	0.000110909	0.00100085	1	0	269	10
2	THAP2	T10	0.000121016	0.00106637	1	0	269	13
2	TOMM20	T12	0.000121907	0.00106793	1	0	108	11
2	ARFRP1	T2	0.00016959	0.00140357	1	0	204	11
2	PFDN2	T12	0.000178697	0.00144696	1	0	109	14

2	HOOK3	T5	0.00019534	0.00154825	1	0	189	19
2	DNAJC5	T2	0.000204457	0.00161198	1	0	206	10
2	SUCO	T12	0.00021486	0.00165344	1	0	105	12
2	PPOX	T12	0.000269721	0.00201016	1	0	109	13
2	EED	T6	0.000370203	0.00262827	1	0	193	8
2	SPAG4	T12	0.000378065	0.00267142	1	0	223	9
2	HEATR1	T12	0.000424096	0.00288771	1	0	107	11
2	SDHC	T12	0.000424069	0.00288771	1	0	108	13
2	UCKL1	T2	0.000430583	0.00290547	1	0	205	10
2	EPB41L1	T12	0.000444848	0.00295358	1	0	222	8
2	USP7	T12	0.000440491	0.00295358	1	0	161	10
2	NFS1	T12	0.000515041	0.00333996	1	0	222	9
2	C6ORF203	T13	0.000528953	0.00341068	1	0	212	11
2	ARFGAP1	T2	0.000686758	0.00430445	1	0	207	11
2	PCMTD2	T2	0.000733485	0.00448853	1	0	205	10
2	TPD52L2	T2	0.00095191	0.00558695	1	0	205	10
2	RTEL1	T2	0.000976271	0.00569048	1	0	204	12
2	TMBIM4	T10	0.001049111	0.00604449	1	0	271	9
2	ZGPAT	T2	0.001057451	0.0060692	1	0	205	11
2	HELB	T10	0.001081822	0.00616186	1	0	269	10
2	PRPF6	T2	0.00137929	0.00748614	1	0	206	10
2	PEX19	T12	0.001424626	0.00770429	1	0	111	10
2	UFC1	T12	0.001490388	0.00800216	1	0	109	13
2	C11ORF73	T6	0.001536744	0.0081634	1	0	193	7
2	TOMM40L	T12	0.001539285	0.0081634	1	0	108	13
2	CPNE1	T12	0.001599468	0.00840703	1	0	222	9
2	HACE1	T10, T13	0.002376461	0.01219157	1	0	213	7
2	PHF20	T12	0.00265065	0.01336927	1	0	222	8
2	ZNF512B	T2	0.002928927	0.01433834	1	0	206	10
2	NDRG3	T12	0.00322221	0.01562094	1	0	222	6
2	MANBAL	T12	0.004452767	0.02046087	1	0	221	6
2	TMEM135	T6	0.005874789	0.02558266	1	0	194	6
2	TRA2A	T10	0.007920815	0.0327773	1	0	261	5
2	MALSU1	T10	0.008902177	0.03621538	1	0	260	5
2	FOXO3	T13	0.009294762	0.03752979	1	0	220	5
2	EIF3M	T12	0.009756179	0.03897268	1	0	261	5
2	TOMM7	T10	0.010385928	0.04105045	1	0	259	5
2	KLHL7	T10	0.011889694	0.04614187	1	0	260	5
2	CYCS	T10	0.012240535	0.04701621	1	0	256	5
2	NUPL2	T10	0.012162973	0.04701621	1	0	260	5
2	SEC63	T13	0.012188172	0.04701621	1	0	215	5
2	LMX1B	T9	3.17E-76	4.75E-73	0	1	264	2
2	DST	T12	9.44E-10	3.63E-08	0	1	267	3
2	IFT140	T4	1.34E-07	2.92E-06	0	1	160	12
2	NENF	T12	2.07E-07	4.30E-06	0	1	102	11
2	TSHZ3	T3	3.00E-07	6.15E-06	0	1	283	3
2	STX6	T12	6.65E-07	1.23E-05	0	1	103	11
2	RPUSD1	T4	2.14E-06	3.73E-05	0	1	160	12
2	NARFL	T4	2.37E-06	4.03E-05	0	1	160	12
2	CEP350	T12	3.32E-06	5.40E-05	0	1	102	11
2	TXNDC11	T12	4.56E-06	7.19E-05	0	1	157	10
2	ZNF669	T8	4.55E-06	7.19E-05	0	1	109	11
2	HAGH	T4	6.42E-06	9.82E-05	0	1	160	12
2	FLVCR1	T12	6.82E-06	0.00010324	0	1	101	11
2	NCSTN	T12	4.00E-05	0.00042544	0	1	111	10
2	LPP	T3	0.000116678	0.00104661	0	1	276	6
2	RAB23	T12	0.000119119	0.00105796	0	1	267	2
2	FBXO30	T10	0.00016574	0.00137933	0	1	233	3
2	ALKBH3	T9	0.001133535	0.00643195	0	1	266	3
2	TPX2	T12, T2	0.004284498	0.01987052	0	1	233	2
2	LDLRAD3	T12	0.004721747	0.02149902	0	1	265	3
2	HNRNPA2B1	T10	0.00587039	0.02558266	0	1	258	4
2	PRR15	T10	0.007405236	0.03106621	0	1	256	4
2	TMEM138	T11	0.007424368	0.03106621	0	1	242	2
2	OPTN	T5	0.010264628	0.04067834	0	1	275	4
2	GSTA4	T12	0.010725968	0.04206152	0	1	265	3
2	NAT10	T12, T3	0.011446251	0.0446523	0	1	263	4
3	ZNF703	T2, T4	6.40E-10	2.52E-08	0	0	156	51
3	GRHL2	T3	1.48E-09	5.28E-08	0	0	153	33
3	EIF3H	T10, T11	7.51E-09	2.39E-07	0	0	147	33
3	MAL2	T11	8.75E-09	2.67E-07	0	0	148	34
3	TMEM65	T11, T3	2.37E-08	6.69E-07	0	0	147	34
3	ZNF572	T11, T3	5.38E-08	1.37E-06	0	0	147	34
3	MTSS1	T11, T3	3.58E-07	7.16E-06	0	0	147	34
3	ARL8A	T1	3.90E-07	7.59E-06	0	0	95	17
3	ZHX1	T1, T3	3.88E-07	7.59E-06	0	0	147	30
3	PPP1R15B	T1	4.68E-07	8.88E-06	0	0	94	19
3	ST3GAL1	T11	7.45E-07	1.36E-05	0	0	151	19
3	LETM2	T2	1.97E-06	3.47E-05	0	0	157	44
3	SAMD12	T11	3.98E-06	6.41E-05	0	0	149	34
3	FZD6	T3	1.08E-05	0.00015494	0	0	152	31
3	INTS7	T12	1.09E-05	0.00015494	0	0	103	12
3	SCYL3	T5	1.29E-05	0.00017959	0	0	105	12
3	CD46	T12	1.42E-05	0.0001956	0	0	95	16
3	NDUFB10	T4	1.73E-05	0.00023182	0	0	160	12
3	IVNS1ABP	T12	1.89E-05	0.00024459	0	0	102	12
3	TELO2	T4	2.33E-05	0.00029137	0	0	160	12
3	UNKL	T4	2.39E-05	0.00029535	0	0	160	12
3	ARID4B	T12	2.49E-05	0.00030358	0	0	108	11
3	C1ORF27	T12	2.52E-05	0.00030432	0	0	102	11
3	TOR1AIP1	T12	2.85E-05	0.00033829	0	0	102	11
3	NDRG1	T11	2.93E-05	0.00034556	0	0	150	20
3	CPT1A	T6	2.98E-05	0.00034897	0	0	221	32
3	IPO9	T1	3.07E-05	0.00035606	0	0	93	16
3	PIGQ	T4	3.28E-05	0.00036949	0	0	160	12
3	PPP2R5A	T12	3.24E-05	0.00036949	0	0	102	11
3	DTL	T12	4.27E-05	0.00044782	0	0	103	12
3	SOX13	T1	4.25E-05	0.00044782	0	0	94	19
3	UCHL5	T6	4.75E-05	0.0004942	0	0	103	12
3	SPSB3	T4	5.30E-05	0.00052535	0	0	160	12
3	ZBTB41	T6	5.29E-05	0.00052535	0	0	97	16
3	NEK2	T12	5.81E-05	0.00056491	0	0	103	12
3	NUAK2	T1	6.33E-05	0.00060787	0	0	96	19
3	ACBD6	T12	6.42E-05	0.0006126	0	0	102	11
3	LRP5	T6	7.82E-05	0.00073241	0	0	231	21
3	FAHD1	T4	7.93E-05	0.00073783	0	0	160	12
3	EXT1	T11	8.29E-05	0.0007615	0	0	150	34
3	RALGPS2	T12	0.000119356	0.00105796	0	0	102	10
3	NUBP1	T12	0.000127001	0.00110609	0	0	159	9
3	PVRL4	T12	0.000130253	0.00112786	0	0	109	14
3	GFER	T4	0.000135012	0.00116234	0	0	160	12
3	CLCN7	T4	0.000139537	0.00119443	0	0	160	12
3	ARPC5	T12, T1	0.000143911	0.00122488	0	0	102	12
3	EEF1A2	T2	0.000156879	0.00132025	0	0	205	12
3	TROVE2	T6	0.000156782	0.00132025	0	0	103	12
3	USP21	T12	0.000158384	0.00132547	0	0	109	13
3	LPGAT1	T12	0.000173019	0.0014163	0	0	103	12
3	KLF10	T3	0.000176829	0.00143962	0	0	152	32
3	ZNF598	T4	0.000183847	0.00148066	0	0	160	12
3	TMEM186	T12	0.000188993	0.00151397	0	0	161	9
3	MAPKAPK2	T12, T1	0.000191156	0.00152314	0	0	96	18
3	ABL2	T12	0.00020748	0.00162725	0	0	102	10
3	MRPS34	T4	0.000209886	0.00162906	0	0	160	12
3	RAB11FIP3	T4	0.00020983	0.00162906	0	0	160	12
3	KIFAP3	T5	0.000215234	0.00165344	0	0	106	12
3	UBE2I	T4	0.000232738	0.00177878	0	0	160	12
3	GLRX2	T6	0.000236178	0.00179591	0	0	103	12
3	ZHX2	T1, T3	0.000251055	0.00189939	0	0	148	30
3	TSC2	T4	0.00025672	0.00193249	0	0	160	12
3	MYC	T11, T3	0.000261802	0.0019609	0	0	142	35
3	WDR24	T4	0.000272149	0.00201821	0	0	160	12
3	TRIB1	T11, T3	0.000277506	0.00203777	0	0	148	34
3	PLEKHA6	T1	0.000335536	0.00242818	0	0	94	19
3	UCP3	T6	0.000345662	0.00248943	0	0	214	14
3	IKBKE	T12, T1	0.000352264	0.00252484	0	0	96	18
3	GGPS1	T12	0.000359901	0.0025673	0	0	108	11
3	TSR3	T4	0.000382638	0.00269104	0	0	160	12
3	RHOT2	T4	0.000402489	0.00281743	0	0	160	12
3	RASAL2	T12	0.000414304	0.00287659	0	0	103	10
3	TIPRL	T5	0.000414782	0.00287659	0	0	107	9
3	GMEB2	T2	0.000418185	0.00288683	0	0	205	12
3	IRF2BP2	T12	0.000423982	0.00288771	0	0	108	10

3	DARS2	T12	0.000427384	0.00289693	0	0	105	9
3	CENPL	T12	0.000443183	0.00295358	0	0	105	9
3	NUBP2	T4	0.0004456	0.00295358	0	0	160	12
3	MSRB1	T4	0.000480485	0.00317078	0	0	160	12
3	TOR1AIP2	T12	0.000491561	0.00322964	0	0	102	11
3	ELF3	T1	0.000512741	0.0033395	0	0	93	17
3	STAR	T2	0.000530499	0.00341068	0	0	153	46
3	SPIDR	T2	0.00055868	0.00357651	0	0	182	14
3	LITAF	T12	0.000583214	0.00371768	0	0	158	12
3	CNTN2	T1	0.000634184	0.00400847	0	0	95	19
3	MR1	T12	0.000633971	0.00400847	0	0	103	11
3	NTHL1	T4	0.00066217	0.00416778	0	0	160	12
3	RNPEP	T1	0.000699608	0.00436672	0	0	93	17
3	C16ORF72	T12	0.000714351	0.00442189	0	0	161	10
3	VAMP4	T12, T5	0.000724766	0.0044679	0	0	105	10
3	NUCKS1	T1	0.000734105	0.00448853	0	0	95	18
3	TIMM17A	T1	0.000738004	0.00449402	0	0	93	16
3	KCNMB3	T13	0.00075706	0.00457289	0	0	269	11
3	RAB40C	T4	0.000754838	0.00457289	0	0	160	12
3	IRF6	T12	0.000768488	0.00462327	0	0	100	14
3	ZNF124	T8	0.000785939	0.00470935	0	0	109	11
3	LIME1	T2	0.00080785	0.00481007	0	0	205	11
3	PRDX6	T12	0.000809171	0.00481007	0	0	105	9
3	TATDN3	T12	0.000835401	0.00494636	0	0	101	11
3	COA6	T12	0.000954779	0.00558695	0	0	108	9
3	ASPM	T6	0.000993044	0.00576582	0	0	97	16
3	ZNF695	T3, T8	0.001005065	0.00581308	0	0	109	11
3	SMG7	T12, T1	0.001071998	0.00612921	0	0	102	12
3	KCTD3	T6	0.001146658	0.00648186	0	0	101	10
3	CRAMP1L	T4	0.001169186	0.00658069	0	0	160	12
3	NDUFS2	T12	0.001172926	0.00658069	0	0	108	13
3	RBM34	T12	0.001208021	0.0067523	0	0	108	11
3	ELK4	T1	0.001235982	0.00685741	0	0	95	18
3	ZNF704	T6	0.001234754	0.00685741	0	0	161	23
3	B3GALNT2	T12	0.00124907	0.00687907	0	0	108	11
3	TPR	T12	0.001244506	0.00687907	0	0	102	11
3	EMP2	T12	0.001323375	0.0072616	0	0	159	9
3	LRP12	T10	0.001344759	0.007352	0	0	151	30
3	C1ORF53	T6	0.001356724	0.00739045	0	0	96	16
3	FBXL16	T4	0.001456714	0.00784949	0	0	160	12
3	C1ORF116	T12	0.001542218	0.0081634	0	0	96	17
3	KLHDC9	T12	0.001530349	0.0081634	0	0	109	14
3	HN1L	T4	0.001558589	0.00822101	0	0	160	12
3	TFB2M	T3, T8	0.001703747	0.00892382	0	0	109	11
3	TBCE	T12	0.001734643	0.00905399	0	0	108	12
3	KLHL20	T12	0.001834083	0.00953978	0	0	105	9
3	RNASEL	T12	0.001930854	0.01000837	0	0	103	11
3	IGSF8	T12	0.002138439	0.01104614	0	0	111	10
3	CCDC19	T12	0.002317464	0.01192976	0	0	112	10
3	ZBTB37	T12	0.002397966	0.01221821	0	0	105	9
3	ZNF496	T8	0.002396731	0.01221821	0	0	109	10
3	MPC2	T12	0.002490663	0.0126475	0	0	108	8
3	FAM20B	T12	0.002543034	0.01286982	0	0	102	10
3	DEXI	T12	0.002701405	0.01348902	0	0	159	9
3	IGFALS	T4	0.002697787	0.01348902	0	0	160	12
3	RNF122	T2	0.002718095	0.01352726	0	0	150	7
3	QSOX1	T12	0.002805025	0.01386775	0	0	102	11
3	HELZ2	T2	0.002846752	0.01402775	0	0	206	12
3	COPA	T12	0.002868567	0.01408889	0	0	111	10
3	SRMS	T2	0.003059098	0.0149268	0	0	205	12
3	CREG1	T12	0.003178666	0.01545987	0	0	109	7
3	NOXO1	T4	0.003288741	0.01589204	0	0	160	12
3	CHRNB3	T5	0.003306192	0.015925	0	0	188	20
3	BLCAP	T12	0.003578877	0.0171283	0	0	218	7
3	C20ORF195	T2	0.00390351	0.01856336	0	0	205	12
3	EDEM3	T12	0.0039185	0.01857567	0	0	103	11
3	CCDC81	T6	0.003974544	0.01872285	0	0	193	7
3	UNC5D	T2	0.004077024	0.0191454	0	0	162	21
3	RGS19	T2	0.004191355	0.01962078	0	0	206	10
3	CDC73	T6	0.004242889	0.01973866	0	0	102	13
3	YOD1	T12	0.004307827	0.01991705	0	0	96	17
3	SCCPDH	T3, T8	0.004536546	0.02071874	0	0	109	11
3	FAIM3	T12	0.004793545	0.02175979	0	0	96	18
3	C16ORF13	T4	0.004861215	0.0220003	0	0	160	12
3	GNPTG	T4	0.00506286	0.02284387	0	0	160	12
3	TBL3	T4	0.005079641	0.02285076	0	0	160	12
3	WDR90	T4	0.005142084	0.0230624	0	0	160	12
3	DLGAP4	T12	0.005252424	0.02341706	0	0	221	8
3	IGSF9	T12	0.00524229	0.02341706	0	0	111	10
3	GPATCH2	T6	0.005403263	0.02401807	0	0	103	10
3	TDRD5	T12	0.005568922	0.0246812	0	0	102	11
3	NOX4	T6	0.005612504	0.02480098	0	0	189	6
3	PPP1R12B	T1	0.005696162	0.02502302	0	0	94	19
3	CLEC16A	T12	0.005810216	0.02544942	0	0	159	9
3	PFKFB2	T12	0.005892231	0.02558424	0	0	96	17
3	LYPLAL1	T6	0.006189479	0.02664322	0	0	103	10
3	PLEKHA2	T2	0.006189359	0.02664322	0	0	166	36
3	ADAMTS4	T12	0.00624065	0.02678652	0	0	109	13
3	PTK6	T2	0.006349574	0.02717618	0	0	205	12
3	GNB4	T13	0.006402366	0.02726421	0	0	269	9
3	LIN28B	T13	0.006406543	0.02726421	0	0	214	7
3	CNBD2	T12	0.006434096	0.0273039	0	0	222	8
3	LAD1	T1	0.00705387	0.02976534	0	0	93	17
3	COL20A1	T2	0.00727323	0.03060477	0	0	207	11
3	DHX9	T12	0.00760224	0.03172188	0	0	102	11
3	HHAT	T12	0.007776711	0.03235976	0	0	103	12
3	MTR	T12, T6	0.007803696	0.0323821	0	0	107	11
3	LGR5	T10	0.008001889	0.03302157	0	0	269	14
3	TGIF2	T12	0.008125043	0.03343768	0	0	222	8
3	TRAF5	T12	0.008197695	0.03364424	0	0	103	13
3	CTSC	T6	0.008547594	0.03498442	0	0	190	10
3	MPZL1	T12	0.008727243	0.03562237	0	0	109	9
3	PKD1	T4	0.008920877	0.03621538	0	0	160	12
3	UBE2T	T1	0.009109625	0.03688167	0	0	95	17
3	GLUL	T12	0.00943066	0.03797615	0	0	103	11
3	TRIM11	T3	0.009545841	0.03833692	0	0	106	9
3	CPSF6	T10	0.009743783	0.03897268	0	0	265	18
3	TMEM81	T1	0.009944704	0.03962012	0	0	95	19
3	ABAT	T12	0.010218708	0.04060378	0	0	161	9
3	C1ORF74	T12	0.010442467	0.0411653	0	0	100	14
3	KISS1	T1	0.012234666	0.04701621	0	0	94	19
3	SRC	T12	0.012787207	0.04899037	0	0	219	6
3	RNF2	T12	0.012945353	0.04946974	0	0	102	12

Tier 1: copy number-regulated genes (significant adjusted p-value and difference between diploid and amplified cases is 2 standard deviations (SDs) apart in both directions).
Tier 2: copy number-regulated genes (significant adjusted p-value and difference between diploid and amplified cases is 2 SDs apart in one but not both directions).
Tier 3: copy number-regulated genes (significant adjusted p only).
