# Supplementary material for: Intra-tumor genetic heterogeneity and alternative driver genetic alterations in breast cancers with heterogeneous HER2 gene amplification
Source: Genome Biol. 2015 May 22;16(1):107. doi: 10.1186/s13059-015-0657-6 (PMC4440518; doi:10.1186/s13059-015-0657-6)
Supplement: Additional file 8: — Sequencing statistics of the HER2 heterogeneous breast cancer subjected to whole exome sequencing and to targeted capture massively parallel sequencing using a platform containing baits targeting all exons of 273 genes. [file 13059_2015_657_MOESM8_ESM.pdf]

**Additional file 8.** Sequencing statistics of the HER2 heterogeneous breast cancer subjected to whole exome sequencing and to targeted capture massively parallel sequencing using a platform containing baits targeting all exons of 273 genes.

| Case ID | Sample | Total Reads | Mean Coverage | PCT Target Bases 2X | PCT Target Bases 10X | PCT Target Bases 20X | PCT Target Bases 30X | PCT Target Bases 40X | PCT Target Bases 50X | PCT Target Bases 100X | Sequencing platform |
|---|---|---|---|---|---|---|---|---|---|---|---|
| T1 | HER2-positive | 95,809,294 | 540.39 | 99.20% | 98.96% | 98.83% | 98.71% | 98.44% | 97.76% | 86.72% | Targeted sequencing of 273 genes frequently mutated in breast cancer |
| T1 | HER2-negative | 108,792,619 | 776.81 | 99.18% | 98.94% | 98.78% | 98.62% | 98.19% | 97.34% | 89.39% | Targeted sequencing of 273 genes frequently mutated in breast cancer |
| T1 | Normal | 75,231,689 | 692.01 | 99.20% | 98.91% | 98.75% | 98.39% | 97.38% | 95.98% | 86.92% | Targeted sequencing of 273 genes frequently mutated in breast cancer |
| T3 | HER2-positive | 67,513,517 | 292.89 | 98.72% | 98.26% | 97.82% | 96.34% | 91.71% | 83.20% | 46.10% | Targeted sequencing of 273 genes frequently mutated in breast cancer |
| T3 | HER2-negative | 96,504,449 | 450.43 | 98.91% | 98.50% | 98.26% | 98.03% | 97.44% | 95.74% | 65.57% | Targeted sequencing of 273 genes frequently mutated in breast cancer |
| T3 | Normal | 56,107,533 | 305.53 | 98.85% | 98.45% | 98.18% | 97.80% | 96.42% | 92.51% | 52.42% | Targeted sequencing of 273 genes frequently mutated in breast cancer |
| T4 | HER2-positive | 50,241,782 | 165.42 | 98.63% | 98.22% | 97.95% | 97.70% | 97.47% | 97.23% | 82.24% | Targeted sequencing of 273 genes frequently mutated in breast cancer |
| T4 | HER2-negative | 47,040,127 | 120.99 | 98.59% | 98.05% | 97.64% | 97.19% | 95.85% | 92.31% | 43.49% | Targeted sequencing of 273 genes frequently mutated in breast cancer |
| T4 | Normal | 40,260,973 | 135.00 | 98.70% | 98.25% | 97.94% | 97.72% | 97.52% | 97.16% | 71.66% | Targeted sequencing of 273 genes frequently mutated in breast cancer |
| T8 | HER2-positive | 59,122,043 | 243.85 | 98.88% | 98.44% | 98.24% | 98.09% | 97.95% | 97.83% | 97.13% | Targeted sequencing of 273 genes frequently mutated in breast cancer |
| T8 | HER2-negative | 93,392,969 | 388.46 | 98.97% | 98.59% | 98.42% | 98.27% | 98.16% | 98.06% | 96.99% | Targeted sequencing of 273 genes frequently mutated in breast cancer |
| T8 | Normal | 48,827,302 | 247.47 | 98.90% | 98.47% | 98.25% | 98.09% | 97.96% | 97.84% | 95.79% | Targeted sequencing of 273 genes frequently mutated in breast cancer |
| T9 | HER2-positive | 72,021,110 | 298.05 | 98.85% | 98.54% | 98.32% | 98.17% | 97.99% | 97.51% | 81.03% | Targeted sequencing of 273 genes frequently mutated in breast cancer |
| T9 | HER2-negative | 61,174,704 | 306.75 | 98.87% | 98.54% | 98.38% | 98.26% | 98.15% | 98.06% | 97.46% | Targeted sequencing of 273 genes frequently mutated in breast cancer |
| T9 | Normal | 82,512,645 | 564.75 | 99.00% | 98.71% | 98.56% | 98.45% | 98.37% | 98.30% | 97.95% | Targeted sequencing of 273 genes frequently mutated in breast cancer |
| T12 | HER2-positive | 47,202,108 | 226.67 | 98.90% | 98.50% | 98.26% | 98.07% | 97.86% | 97.63% | 94.88% | Targeted sequencing of 273 genes frequently mutated in breast cancer |
| T12 | HER2-negative | 50,965,403 | 200.18 | 98.92% | 98.47% | 98.19% | 97.89% | 97.57% | 97.20% | 91.95% | Targeted sequencing of 273 genes frequently mutated in breast cancer |
| T12 | Normal | 64,245,194 | 317.73 | 98.94% | 98.58% | 98.37% | 98.18% | 97.95% | 97.78% | 96.26% | Targeted sequencing of 273 genes frequently mutated in breast cancer |
| T6 | HER2-positive | 71,246,443 | 56.78 | 95.54% | 88.67% | 79.27% | 67.04% | 54.23% | 42.82% | 13.03% | Whole exome sequencing |
| T6 | HER2-negative | 69,076,811 | 57.96 | 93.73% | 84.78% | 74.54% | 63.67% | 53.11% | 43.52% | 14.95% | Whole exome sequencing |
| T6 | Normal | 106,077,251 | 85.01 | 95.13% | 88.39% | 82.34% | 76.30% | 69.69% | 62.61% | 30.54% | Whole exome sequencing |
| T11 | HER2-positive | 99,608,421 | 80.70 | 94.88% | 87.97% | 81.70% | 75.21% | 68.04% | 60.29% | 27.63% | Whole exome sequencing |
| T11 | HER2-negative | 67,667,683 | 55.67 | 94.67% | 86.42% | 76.59% | 65.49% | 54.13% | 43.66% | 13.41% | Whole exome sequencing |
| T11 | Normal | 61,190,636 | 50.29 | 94.56% | 85.99% | 75.55% | 63.16% | 50.47% | 39.07% | 10.10% | Whole exome sequencing |
| T12 | HER2-positive | 323,195,245 | 254.25 | 93.71% | 91.29% | 89.20% | 87.06% | 84.92% | 82.80% | 72.21% | Whole exome sequencing |
| T12 | HER2-negative | 314,364,349 | 248.59 | 93.63% | 91.14% | 88.97% | 86.76% | 84.54% | 82.34% | 71.30% | Whole exome sequencing |
| T12 | Normal | 131,167,048 | 114.97 | 92.58% | 88.86% | 84.38% | 79.96% | 75.53% | 71.06% | 47.95% | Whole exome sequencing |

PCT, percentage
