# Supplementary material for: Intra-tumor genetic heterogeneity and alternative driver genetic alterations in breast cancers with heterogeneous HER2 gene amplification
Source: Genome Biol. 2015 May 22;16(1):107. doi: 10.1186/s13059-015-0657-6 (PMC4440518; doi:10.1186/s13059-015-0657-6)
Supplement: Additional file 9: — Mutations identified by whole exome sequencing analysis and validated by amplicon sequencing on an Ion Torrent Personal Genome Machine or by targeted capture massively parallel sequencing on an Illumina HiSeq2000. [file 13059_2015_657_MOESM9_ESM.pdf]

Additional file 9. Mutations identified by whole exome sequencing analysis and validated by amplicon sequencing on an Ion Torrent Personal Genome Machine or by targeted capture massively parallel sequencing on an Illumina HiSeq2000.

Case ID	Gene Symbol	Consequence	Amino acid change	Mutation Taster	CHASM (breast)	Mutant allele fraction (HER2-postive component)	Mutant allele fraction (HER2-negative component)	Read depth (HER2-positive component)	Read depth (HER2-negative component)	Cancer Cell Fraction (HER2-positive component)	Cancer Cell Fraction (HER2-negative component)	CHROM	POS	REF	ALT	Cancer Gene Census	127 genes Kandoth et al	Cancer 5000-S	Pathogenic by predictor	Pathogenic	Validation
T11	HCFC1	NON_SYNONYMOUS_CODING	V687L	disease_causing	Passenger	0.00%	10.66%	9677	7900	0.00	0.81	X	153223307	C	G				Pathogenic	Pathogenic	Ion Torrent
T11	NRP1	NON_SYNONYMOUS_CODING	R767H	disease_causing	Passenger	0.00%	7.75%	NA	555	0.00	0.59	10	33475179	C	T				Pathogenic	Pathogenic	Ion Torrent
T11	PTTG1IP	NON_SYNONYMOUS_CODING	R127W	polymorphism	Passenger	0.00%	21.70%	10123	34662	0.00	1.00	21	46276178	G	A				Non-Pathogenic	Non-Pathogenic	Ion Torrent
T11	AMMECR1	NON_SYNONYMOUS_CODING	L138R	disease_causing	Passenger	17.43%	22.14%	12986	9000	1.00	1.00	X	109560887	A	C				Pathogenic	Pathogenic	Ion Torrent
T11	C1orf9	NON_SYNONYMOUS_CODING	V1242M	disease_causing	Passenger	20.66%	36.34%	4419	5228	1.00	1.00	1	172579358	G	A				Pathogenic	Pathogenic	Ion Torrent
T11	COMP	NON_SYNONYMOUS_CODING	A419V	polymorphism	Passenger	21.17%	11.74%	10267	13326	1.00	0.90	19	18897100	G	A				Non-Pathogenic	Non-Pathogenic	Ion Torrent
T11	TP53	ESSENTIAL_SPLICE_SITE	.	.	.	30.51%	25.71%	27670	16753	1.00	1.00	17	7578275	GAGGAGGGGCCAGACCTAA	G	TRUE	TRUE	TRUE	Pathogenic	Pathogenic	Ion Torrent
T12	ADAM29	NON_SYNONYMOUS_CODING	A513T	disease_causing	Passenger	0.00%	17.80%	139	180	0.00	1.00	4	175898213	G	A				Pathogenic	Pathogenic	
T12	BPI	NON_SYNONYMOUS_CODING	S145L	polymorphism	Passenger	0.00%	8.20%	1021	742	0.00	1.00	20	36938940	C	T				Non-Pathogenic	Non-Pathogenic	
T12	FANCD2	NON_SYNONYMOUS_CODING	L1394F	disease_causing	Passenger	0.00%	13.90%	93	144	0.00	0.96	3	10138153	G	T			TRUE	Pathogenic	Pathogenic	Targeted sequencing (Illumina)
T12	KRTAP10-2	NON_SYNONYMOUS_CODING	C42S	disease_causing	Passenger	0.00%	8.60%	76	58	0.00	0.43	21	45971218	A	T				Pathogenic	Pathogenic	
T12	LINGO1	NON_SYNONYMOUS_CODING	A560V	polymorphism	Passenger	0.00%	25.70%	319	175	0.00	1.00	15	77906570	G	A				Non-Pathogenic	Non-Pathogenic	
T12	LRFN1	NON_SYNONYMOUS_CODING	R214H	disease_causing	Passenger	0.00%	4.20%	874	765	0.00	0.67	19	39805336	C	T				Pathogenic	Pathogenic	
T12	NASP	NON_SYNONYMOUS_CODING	P372T	polymorphism	Passenger	0.00%	17.90%	27	28	0.00	0.88	1	46073697	C	A				Non-Pathogenic	Non-Pathogenic	
T12	OR2T4	NON_SYNONYMOUS_CODING	H86Y	polymorphism	Passenger	0.00%	11.30%	70	71	0.00	0.78	1	248525138	C	T				Non-Pathogenic	Non-Pathogenic	
T12	OR2T4	NON_SYNONYMOUS_CODING	A85T	polymorphism	Passenger	0.00%	11.80%	69	68	0.00	0.82	1	248525135	G	A				Non-Pathogenic	Non-Pathogenic	
T12	PPIAL4G	NON_SYNONYMOUS_CODING	A128V	polymorphism	Passenger	0.00%	16.70%	28	36	0.00	1.00	1	143767466	G	A				Non-Pathogenic	Non-Pathogenic	
T12	PROS1	NON_SYNONYMOUS_CODING	R330Q	polymorphism	Passenger	0.00%	7.60%	138	157	0.00	0.53	3	93611943	C	T				Non-Pathogenic	Non-Pathogenic	
T12	RTN3	NON_SYNONYMOUS_CODING	P528L	disease_causing	Passenger	0.00%	24.40%	188	221	0.00	1.00	11	63487557	C	T				Pathogenic	Pathogenic	
T12	SEMA3E	NON_SYNONYMOUS_CODING	R337Q	polymorphism	Passenger	0.00%	20.10%	134	139	0.00	1.00	7	83032081	C	T				Non-Pathogenic	Non-Pathogenic	
T12	SGTB	NON_SYNONYMOUS_CODING	T209I	polymorphism	Passenger	0.00%	13.60%	203	242	0.00	0.67	5	64976376	G	A				Non-Pathogenic	Non-Pathogenic	
T12	SLC31A1	NON_SYNONYMOUS_CODING	D37N	polymorphism	Passenger	0.00%	20.20%	276	248	0.00	1.00	9	116018537	G	A				Non-Pathogenic	Non-Pathogenic	
T12	TTN	NON_SYNONYMOUS_CODING	T14842S	polymorphism	Passenger	0.00%	21.30%	278	254	0.00	1.00	2	179490023	G	C				Non-Pathogenic	Non-Pathogenic	
T12	ZBTB24	NON_SYNONYMOUS_CODING	H684Y	disease_causing	Passenger	0.00%	14.80%	333	331	0.00	1.00	6	109787098	G	A				Pathogenic	Pathogenic	
T12	ZNF492	NON_SYNONYMOUS_CODING	T409I	disease_causing	.	0.00%	14.30%	59	56	0.00	1.00	19	22847697	C	T				Pathogenic	Pathogenic	
T12	ZNF566	STOP_GAINED	R273*	disease_causing	Passenger	0.00%	14.90%	248	249	0.00	1.00	19	36940322	G	A				Pathogenic	Pathogenic	
T12	RERE	NON_SYNONYMOUS_CODING	E52A	disease_causing	Passenger	3.90%	0.00%	311	347	0.17	0.00	1	8716202	T	G				Pathogenic	Pathogenic	
T12	EMR2	START_GAINED	.	.	.	4.20%	0.00%	142	92	0.18	0.00	19	14887656	G	A					Non-Pathogenic	
T12	C5orf28	STOP_GAINED	R158*	disease_causing	.	4.40%	0.00%	298	371	0.23	0.00	5	43446500	G	A				Pathogenic	Pathogenic	
T12	MRGPRX3	NON_SYNONYMOUS_CODING	E11K	polymorphism	Passenger	2.70%	0.00%	708	1231	0.25	0.00	11	18158780	G	A				Non-Pathogenic	Non-Pathogenic	
T12	LHX1	NON_SYNONYMOUS_CODING	D13Y	disease_causing	Passenger	6.30%	0.00%	319	356	0.27	0.00	17	35295531	G	T				Pathogenic	Pathogenic	
T12	CSGALNACT	STOP_GAINED	R347*	disease_causing	.	5.40%	0.00%	93	105	0.29	0.00	10	43659372	C	T				Pathogenic	Pathogenic	
T12	MICALL1	NON_SYNONYMOUS_CODING	S644Y	disease_causing	Passenger	6.80%	0.00%	132	101	0.30	0.00	22	38327855	C	A				Pathogenic	Pathogenic	
T12	FBN2	NON_SYNONYMOUS_CODING	R245Q	disease_causing	Passenger	7.60%	0.00%	79	111	0.33	0.00	5	127800509	C	T				Pathogenic	Pathogenic	
T12	FAT3	NON_SYNONYMOUS_CODING	Q3214K	polymorphism	Passenger	6.60%	0.00%	488	530	0.35	0.00	11	92564946	C	A				Non-Pathogenic	Non-Pathogenic	Targeted sequencing (Illumina)
T12	LLGL2	NON_SYNONYMOUS_CODING	R855Q	polymorphism	Passenger	6.80%	0.00%	118	116	0.36	0.00	17	73569198	G	A				Non-Pathogenic	Non-Pathogenic	
T12	APOBR	NON_SYNONYMOUS_CODING	G364W	polymorphism	Passenger	7.00%	0.00%	71	55	0.38	0.00	16	28507452	G	T				Non-Pathogenic	Non-Pathogenic	
T12	AGGF1	NON_SYNONYMOUS_CODING	V202L	disease_causing	Passenger	9.10%	0.00%	88	86	0.40	0.00	5	76332468	G	T				Pathogenic	Pathogenic	
T12	CNTNAP5	NON_SYNONYMOUS_CODING	P954T	disease_causing	Passenger	7.60%	0.00%	329	355	0.41	0.00	2	125547589	C	A				Pathogenic	Pathogenic	
T12	GPNMB	NON_SYNONYMOUS_CODING	P130L	disease_causing	Passenger	9.60%	0.00%	146	199	0.42	0.00	7	23296532	C	T				Pathogenic	Pathogenic	
T12	GNAQ	NON_SYNONYMOUS_CODING	T96S	disease_causing	Passenger	8.80%	0.00%	113	182	0.47	0.00	9	80537112	T	A			TRUE	Pathogenic	Pathogenic	
T12	MUC4	NON_SYNONYMOUS_CODING	A3510T	polymorphism	Passenger	10.00%	0.00%	60	22	0.64	0.00	3	195507923	C	T				Non-Pathogenic	Non-Pathogenic	
T12	SIPA1L3	NON_SYNONYMOUS_CODING	L1671P	disease_causing	Passenger	7.00%	13.70%	761	766	0.65	1.00	19	38692529	T	C				Pathogenic	Pathogenic	
T12	SNAI2	NON_SYNONYMOUS_CODING	A173P	disease_causing	Passenger	9.10%	0.00%	231	221	0.67	0.00	8	49832563	C	G				Pathogenic	Pathogenic	
T12	MCL1	NON_SYNONYMOUS_CODING	T92N	polymorphism	Passenger	10.80%	19.20%	212	224	0.69	1.00	1	150551732	G	T				Non-Pathogenic	Non-Pathogenic	
T12	OBSCN	NON_SYNONYMOUS_CODING	R4892W	polymorphism	Passenger	10.40%	13.30%	367	249	0.76	1.00	1	228494216	C	T				Non-Pathogenic	Non-Pathogenic	Targeted sequencing (Illumina)
T12	IFNA7	NON_SYNONYMOUS_CODING	S31T	polymorphism	Passenger	17.90%	0.00%	67	63	0.78	0.00	9	21202073	C	G				Non-Pathogenic	Non-Pathogenic	
T12	MUC4	NON_SYNONYMOUS_CODING	G3509S	polymorphism	Passenger	12.50%	0.00%	56	20	0.79	0.00	3	195507926	C	T				Non-Pathogenic	Non-Pathogenic	
T12	MGAM	NON_SYNONYMOUS_CODING	R1121H	disease_causing	Passenger	11.00%	0.00%	834	888	0.81	0.00	7	141755405	G	A				Pathogenic	Pathogenic	Targeted sequencing (Illumina)
T12	PRB4	NON_SYNONYMOUS_CODING	P50T	polymorphism	Passenger	20.00%	13.70%	45	51	0.87	0.95	12	11461769	G	T				Non-Pathogenic	Non-Pathogenic	
T12	PRTFDC1	NON_SYNONYMOUS_CODING	D19H	disease_causing	Passenger	20.20%	20.90%	94	110	0.88	1.00	10	25231359	C	G				Pathogenic	Pathogenic	
T12	ARMC9	NON_SYNONYMOUS_CODING	K505R	disease_causing	Passenger	17.40%	22.60%	155	177	0.93	1.00	2	232143134	A	G				Pathogenic	Pathogenic	
T12	EML1	NON_SYNONYMOUS_CODING	V597I	disease_causing	Passenger	14.90%	18.60%	154	129	0.95	1.00	14	100381014	G	A				Pathogenic	Pathogenic	
T12	ABCD1	NON_SYNONYMOUS_CODING	R452Q	polymorphism	Passenger	37.40%	32.10%	131	112	1.00	1.00	X	153001929	G	A				Non-Pathogenic	Non-Pathogenic	
T12	ACAD9	NON_SYNONYMOUS_CODING	A390T	disease_causing	Passenger	23.20%	16.90%	181	166	1.00	1.00	3	128624982	G	A				Pathogenic	Pathogenic	
T12	ADAM9	NON_SYNONYMOUS_CODING	C575Y	disease_causing	Passenger	24.50%	21.80%	286	372	1.00	1.00	8	38934773	G	A				Pathogenic	Pathogenic	
T12	ADAMTS20	NON_SYNONYMOUS_CODING	N1750K	disease_causing	Passenger	43.50%	36.50%	177	203	1.00	1.00	12	43769922	G	C				Pathogenic	Pathogenic	
T12	APOBR	NON_SYNONYMOUS_CODING	E361D	polymorphism	Passenger	18.80%	12.20%	69	49	1.00	0.73	16	28507445	G	C				Non-Pathogenic	Non-Pathogenic	
T12	ASNS	NON_SYNONYMOUS_CODING	E91K	disease_causing	Passenger	20.00%	25.00%	60	64	1.00	1.00	7	97493787	C	T				Pathogenic	Pathogenic	
T12	CD163L1	NON_SYNONYMOUS_CODING	R488T	disease_causing	Passenger	24.10%	0.00%	344	630	1.00	0.00	12	7551126	C	G				Pathogenic	Pathogenic	
T12	CD163L1	NON_SYNONYMOUS_CODING	G498E	disease_causing	Passenger	27.20%	0.00%	481	841	1.00	0.00	12	7551096	C	T				Pathogenic	Pathogenic	
T12	CDX4	NON_SYNONYMOUS_CODING	M54K	polymorphism	Passenger	18.60%	0.00%	365	406	1.00	0.00	X	72667250	T	A				Non-Pathogenic	Non-Pathogenic	
T12	CROCC	NON_SYNONYMOUS_CODING	A439V	polymorphism	Passenger	32.50%	40.90%	40	22	1.00	1.00	1	17264920	C	T				Non-Pathogenic	Non-Pathogenic	
T12	EFEMP2	NON_SYNONYMOUS_CODING	E261D	disease_causing	Passenger	53.50%	44.20%	129	95	1.00	1.00	11	65636045	C	G				Pathogenic	Pathogenic	
T12	EHD4	NON_SYNONYMOUS_CODING	E13K	polymorphism	Passenger	35.60%	25.00%	135	104	1.00	1.00	15	42264656	C	T				Non-Pathogenic	Non-Pathogenic	
T12	EPHB1	NON_SYNONYMOUS_CODING	P294S	disease_causing	Passenger	26.00%	35.80%	196	201	1.00	1.00	3	134825364	C	T				Pathogenic	Pathogenic	
T12	FBLN5	NON_SYNONYMOUS_CODING	R71W	disease_causing	Passenger	17.50%	0.00%	154	181	1.00	0.00	14	92403459	G	A				Pathogenic	Pathogenic	
T12	GPRASP1	NON_SYNONYMOUS_CODING	A233V	polymorphism	Passenger	17.10%	17.20%	392	366	1.00	1.00	X	101909539	C	T				Non-Pathogenic	Non-Pathogenic	
T12	IGF2R	NON_SYNONYMOUS_CODING	R91H	polymorphism	Passenger	39.80%	45.90%	128	146	1.00	1.00	6	160412338	G	A				Non-Pathogenic	Non-Pathogenic	
T12	KIT	NON_SYNONYMOUS_CODING	A755T	disease_causing	Passenger	39.60%	45.30%	293	333	1.00	1.00	4	55598066	G	A	TRUE	TRUE	TRUE	Pathogenic	Pathogenic	Targeted sequencing (Illumina)
T12	LMTK3	NON_SYNONYMOUS_CODING	E359K	disease_causing	Passenger	39.40%	40.60%	650	478	1.00	1.00	19	49004553	C	T				Pathogenic	Pathogenic	
T12	MYH4	NON_SYNONYMOUS_CODING	T189M	disease_causing	Passenger	72.20%	65.90%	36	41	1.00	1.00	17	10367871	G	A				Pathogenic	Pathogenic	
T12	NGLY1	NON_SYNONYMOUS_CODING	R542Q	disease_causing	Passenger	46.70%	38.60%	90	166	1.00	1.00	3	25761669	C	T				Pathogenic	Pathogenic	
T12	OR4D5	NON_SYNONYMOUS_CODING	R122H	disease_causing	Passenger	24.30%	0.00%	608	746	1.00	0.00	11	123810688	G	A				Pathogenic	Pathogenic	
T12	OTUD6A	NON_SYNONYMOUS_CODING	G278S	polymorphism	Passenger	37.60%	38.90%	322	296	1.00	1.00	X	69283206	G	A				Non-Pathogenic	Non-Pathogenic	
T12	PRKAR1B	NON_SYNONYMOUS_CODING	V302M	disease_causing	Passenger	27.80%	0.00%	54	42	1.00	0.00	7	591095	C	T				Pathogenic	Pathogenic	
T12	SIX3	NON_SYNONYMOUS_CODING	A264V	disease_causing	Passenger	25.70%	27.20%	269	180	1.00	1.00	2	45170034	C	T				Pathogenic	Pathogenic	
T12	SPATA5	NON_SYNONYMOUS_CODING	M224L	polymorphism	Passenger	22.10%	18.40%	272	365	1.00	1.00	4	123855416	A	T				Non-Pathogenic	Non-Pathogenic	
T12	STAT1	NON_SYNONYMOUS_CODING	Q441H	disease_causing	Passenger	36.70%	46.70%	60	45	1.00	1.00	2	191849060	C	G				Pathogenic	Pathogenic	Targeted sequencing (Illumina)
T12	TMEM121	NON_SYNONYMOUS_CODING	E66K	disease_causing	Passenger	37.30%	41.60%	204	178	1.00	1.00	14	105995367	G	A				Pathogenic	Pathogenic	
T12	TNXB	NON_SYNONYMOUS_CODING	R1210H	polymorphism	Passenger	32.30%	0.00%	133	107	1.00	0.00	6	32049920	C	T				Non-Pathogenic	Non-Pathogenic	
T12	TP53	NON_SYNONYMOUS_CODING	I195N	disease_causing	Driver	76.80%	72.20%	254	216	1.00	1.00	17	7578265	A	T	TRUE	TRUE	TRUE	Pathogenic	Pathogenic	Targeted sequencing (Illumina)
T12	UNC79	NON_SYNONYMOUS_CODING	V272M	disease_causing	Passenger	18.50%	0.00%	81	95	1.00	0.00	14	94004557	G	A				Pathogenic	Pathogenic	
T12	VAV1	SPLICE_SITE	.	disease_causing	.	59.00%	61.70%	78	47	1.00	1.00	19	6828429	G	A				Pathogenic	Pathogenic	
T6	ACPL2	NON_SYNONYMOUS_CODING	R290T	disease_causing	Passenger	0.00%	20.86%	5459	6208	0.00	1.00	3	141011473	G	C				Pathogenic	Pathogenic	Ion Torrent
T6	ARHGEF11	NON_SYNONYMOUS_CODING	M79I	disease_causing	Passenger	0.00%	11.01%	404	972	0.00	0.77	1	156950265	C	T				Pathogenic	Pathogenic	Ion Torrent
T6	ATP8B1	NON_SYNONYMOUS_CODING	R768G	disease_causing	Passenger	0.00%	16.51%	4681	5747	0.00	1.00	18	55329831	T	C				Pathogenic	Pathogenic	Ion Torrent
T6	BRD4	NON_SYNONYMOUS_CODING	E4D	disease_causing	Passenger	0.00%	17.48%	2364	2798	0.00	1.00	19	15383899	C	G	TRUE			Pathogenic	Pathogenic	Ion Torrent
T6	CACNB2	NON_SYNONYMOUS_CODING	P221A	disease_causing	Passenger	0.00%	9.59%	2999	3409	0.00	0.67	10	18795467	C	G				Pathogenic	Pathogenic	Ion Torrent
T6	CBX5	NON_SYNONYMOUS_CODING	S14L	disease_causing	Passenger	0.00%	14.44%	6339	4654	0.00	0.86	12	54651394	G	A				Pathogenic	Pathogenic	Ion Torrent
T6	CHD9	NON_SYNONYMOUS_CODING	G403E	disease_causing	Passenger	0.00%	24.79%	3248	3896	0.00	1.00	16	53191209	G	A				Pathogenic	Pathogenic	Ion Torrent
T6	CYP2S1	NON_SYNONYMOUS_CODING	D260H	disease_causing	Passenger	0.00%	12.44%	4962	4075	0.00	0.74	19	41704737	G	C				Pathogenic	Pathogenic	Ion Torrent
T6	DPYSL5	NON_SYNONYMOUS_CODING	E448K	disease_causing	Passenger	0.00%	13.22%	7213	6704	0.00	0.92	2	27165520	G	A				Pathogenic	Pathogenic	Ion Torrent
T6	DYNC1H1	NON_SYNONYMOUS_CODING	L2315F	disease_causing	Passenger	0.00%	14.80%	5933	7007	0.00	1.00	14	102478738	G	C				Pathogenic	Pathogenic	Ion Torrent
T6	DYNC1H1	NON_SYNONYMOUS_CODING	E4148K	disease_causing	Passenger	0.00%	22.65%	5513	7156	0.00	1.00	14	102509014	G	A				Pathogenic	Pathogenic	Ion Torrent
T6	ERBB2	NON_SYNONYMOUS_CODING	I767M	disease_causing	Driver	0.00%	13.77%	6510	4802	0.00	0.82	17	37880257	C	G	TRUE		TRUE	Pathogenic	Pathogenic	Ion Torrent
T6	ETV5	NON_SYNONYMOUS_CODING	E60K	disease_causing	Passenger	0.00%	14.48%	2669	3011	0.00	1.00	3	185823241	C	T	TRUE			Pathogenic	Pathogenic	Ion Torrent
T6	EVA1C	NON_SYNONYMOUS_CODING	S87L	disease_causing	Passenger	0.00%	15.05%	1719	1196	0.00	1.00	21	33825719	C	T				Pathogenic	Pathogenic	Ion Torrent
T6	FAM185A	NON_SYNONYMOUS_CODING	S248L	disease_causing	Passenger	0.00%	20.53%	2010	2474	0.00	1.00	7	102401808	C	T				Pathogenic	Pathogenic	Ion Torrent
T6	FAM188B2	SPLICE_SITE	.	.	.	0.00%	20.26%	1009	1150	0.00	1.00	3	150600849	G	C					Non-Pathogenic	Ion Torrent
T6	FBXO6	ESSENTIAL_SPLICE_SITE	.	disease_causing	.	0.00%	4.79%	2029	3674	0.00	0.29	1	11731984	G	A				Pathogenic	Pathogenic	Ion Torrent
T6	GLRA4	NON_SYNONYMOUS_CODING	D59N	disease_causing	Passenger	0.00%	18.75%	540	864	0.00	1.00	X	102979853	C	T				Pathogenic	Pathogenic	Ion Torrent
T6	GPHN	NON_SYNONYMOUS_CODING	Q690H	disease_causing	Passenger	0.00%	16.95%	3785	3664	0.00	1.00	14	67646384	G	C	TRUE			Pathogenic	Pathogenic	Ion Torrent
T6	KIAA0913	NON_SYNONYMOUS_CODING	S704C	polymorphism	Passenger	0.00%	15.17%	2599	5545	0.00	1.00	10	75552408	C	G				Non-Pathogenic	Non-Pathogenic	Ion Torrent
T6	LRRC41	NON_SYNONYMOUS_CODING	S615C	polymorphism	Passenger	0.00%	19.46%	1109	2461	0.00	1.00	1	46746145	G	C				Non-Pathogenic	Non-Pathogenic	Ion Torrent
T6	MARCH8	NON_SYNONYMOUS_CODING	P254A	polymorphism	Passenger	0.00%	13.66%	2284	2416	0.00	0.95	10	45953803	G	C				Non-Pathogenic	Non-Pathogenic	Ion Torrent
T6	NBEA	NON_SYNONYMOUS_CODING	L1750V	disease_causing	Passenger	0.00%	26.02%	10108	9619	0.00	1.00	13	35770321	C	G				Pathogenic	Pathogenic	Ion Torrent
T6	NRG2	NON_SYNONYMOUS_CODING	R462W	disease_causing	Passenger	0.00%	14.57%	7173	6581	0.00	1.00	5	139232521	G	A				Pathogenic	Pathogenic	Ion Torrent
T6	PSG5	NON_SYNONYMOUS_CODING	N333S	polymorphism	Passenger	0.00%	16.08%	6428	6424	0.00	0.96	19	43674257	T	C				Non-Pathogenic	Non-Pathogenic	Ion Torrent
T6	RARG	NON_SYNONYMOUS_CODING	E40K	disease_causing	Passenger	0.00%	22.49%	1492	2321	0.00	1.00	12	53621212	C	T				Pathogenic	Pathogenic	Ion Torrent
T6	ROR1	NON_SYNONYMOUS_CODING	Q812H	disease_causing	Passenger	0.00%	23.43%	1938	3747	0.00	1.00	1	64644160	G	C				Pathogenic	Pathogenic	Ion Torrent
T6	RTTN	NON_SYNONYMOUS_CODING	S2060C	disease_causing	Passenger	0.00%	17.85%	3059	3356	0.00	1.00	18	67684885	G	C				Pathogenic	Pathogenic	Ion Torrent
T6	SEPT3	NON_SYNONYMOUS_CODING	T356I	disease_causing	Passenger	0.00%	9.96%	7853	7489	0.00	0.60	22	42392961	C	T				Pathogenic	Pathogenic	Ion Torrent
T6	SLC37A3	SPLICE_SITE	.	polymorphism	.	0.00%	21.76%	4768	5909	0.00	1.00	7	140043205	C	A				Non-Pathogenic	Non-Pathogenic	Ion Torrent
T6	SLC41A3	NON_SYNONYMOUS_CODING	K280N	polymorphism	Passenger	0.00%	16.74%	3376	4700	0.00	1.00	3	125735624	C	G				Non-Pathogenic	Non-Pathogenic	Ion Torrent
T6	TBC1D22A	NON_SYNONYMOUS_CODING	H451D	disease_causing	Passenger	0.00%	19.23%	3932	3849	0.00	1.00	22	47507425	C	G				Pathogenic	Pathogenic	Ion Torrent
T6	UNC13B	NON_SYNONYMOUS_CODING	E362K	disease_causing	Passenger	0.00%	19.07%	5986	6649	0.00	1.00	9	35313903	G	A				Pathogenic	Pathogenic	Ion Torrent
T6	ZNF202	NON_SYNONYMOUS_CODING	D285H	disease_causing	Passenger	0.00%	24.33%	678	1644	0.00	1.00	11	123598283	C	G				Pathogenic	Pathogenic	Ion Torrent
T6	ZNF609	NON_SYNONYMOUS_CODING	S849C	disease_causing	Passenger	0.00%	23.14%	5490	6520	0.00	1.00	15	64967599	C	G				Pathogenic	Pathogenic	Ion Torrent
T6	ZYG11A	NON_SYNONYMOUS_CODING	E32K	disease_causing	Passenger	0.00%	14.31%	5794	6451	0.00	0.86	1	53320140	G	A				Pathogenic	Pathogenic	Ion Torrent
T6	TEKT4	NON_SYNONYMOUS_CODING	H307Q	polymorphism	Passenger	2.51%	3.63%	439	689	0.18	0.25	2	95540728	T	G				Non-Pathogenic	Non-Pathogenic	Ion Torrent
T6	EPS8L2	NON_SYNONYMOUS_CODING	M707I	polymorphism	Passenger	8.25%	17.29%	3065	3921	0.59	1.00	11	726954	G	T				Non-Pathogenic	Non-Pathogenic	Ion Torrent
T6	ANKRD28	SPLICE_SITE	.	disease_causing	.	18.77%	19.57%	1790	2499	1.00	1.00	3	15766038	A	G				Pathogenic	Pathogenic	Ion Torrent
T6	CCDC70	NON_SYNONYMOUS_CODING	F72C	polymorphism	Passenger	23.40%	30.11%	141	279	1.00	1.00	13	52439729	T	G				Non-Pathogenic	Non-Pathogenic	Ion Torrent
T6	CFHR5	NON_SYNONYMOUS_CODING	E74Q	polymorphism	Passenger	15.62%	0.00%	397	2739	1.00	0.00	1	196952176	G	C				Non-Pathogenic	Non-Pathogenic	Ion Torrent
T6	CHMP4A	IN_FRAME_DEL	A62_Q64del	polymorphism	.	17.82%	22.22%	4933	5392	1.00	1.00	14	24680785	AGCCTGTAGG	A				Non-Pathogenic	Non-Pathogenic	Ion Torrent
T6	FNDC1	NON_SYNONYMOUS_CODING	T354R	disease_causing	Passenger	20.08%	25.62%	2903	3091	1.00	1.00	6	159646743	C	G				Pathogenic	Pathogenic	Ion Torrent
T6	MOXD1	NON_SYNONYMOUS_CODING	E557Q	disease_causing	Passenger	15.61%	0.00%	7547	2766	1.00	0.00	6	132618934	C	G				Pathogenic	Pathogenic	Ion Torrent
T6	NRXN3	NON_SYNONYMOUS_CODING	R222C	disease_causing	Passenger	25.09%	28.49%	4117	4959	1.00	1.00	14	80164035	C	T				Pathogenic	Pathogenic	Ion Torrent
T6	OSBPL6	NON_SYNONYMOUS_CODING	S9F	disease_causing	Passenger	20.24%	20.88%	4067	5777	1.00	1.00	2	179170937	C	T				Pathogenic	Pathogenic	Ion Torrent
T6	OTOF	NON_SYNONYMOUS_CODING	D122V	disease_causing	Passenger	20.43%	26.16%	6886	6924	1.00	1.00	2	26739430	T	A				Pathogenic	Pathogenic	Ion Torrent
T6	PIGQ	NON_SYNONYMOUS_CODING	T14A	polymorphism	Passenger	24.75%	33.34%	6627	7735	1.00	1.00	16	624114	A	G				Non-Pathogenic	Non-Pathogenic	Ion Torrent
T6	PIK3CA	NON_SYNONYMOUS_CODING	H1047R	disease_causing	Driver	22.13%	28.89%	3914	5840	1.00	1.00	3	178952085	A	G	TRUE	TRUE	TRUE	Pathogenic	Pathogenic	Ion Torrent
T6	PLCH1	NON_SYNONYMOUS_CODING	L891Q	disease_causing	Passenger	25.33%	22.32%	5803	6609	1.00	1.00	3	155203471	A	T				Pathogenic	Pathogenic	Ion Torrent
T6	SIPA1L2	NON_SYNONYMOUS_CODING	V573I	disease_causing	Passenger	15.36%	17.74%	3777	4155	1.00	1.00	1	232626709	C	T				Pathogenic	Pathogenic	Ion Torrent
T6	SLFN5	NON_SYNONYMOUS_CODING	D126N	polymorphism	Passenger	16.36%	23.52%	550	540	1.00	1.00	17	33586085	G	A				Non-Pathogenic	Non-Pathogenic	Ion Torrent
T6	TP53	NON_SYNONYMOUS_CODING	R273H	disease_causing_automatic	Driver	25.59%	33.09%	2435	3001	1.00	1.00	17	7577120	C	T	TRUE	TRUE	TRUE	Pathogenic	Pathogenic	Ion Torrent
T6	TTC21A	NON_SYNONYMOUS_CODING	G151V	disease_causing	Passenger	15.86%	19.21%	5806	7071	1.00	1.00	3	39153965	G	T				Pathogenic	Pathogenic	Ion Torrent
T6	TYMP	FRAMESHIFT_CODING	L158fs	disease_causing_automatic		21.84%	20.02%	4991	5888	1.00	1.00	22	50966983	C	CA				Pathogenic	Pathogenic	Ion Torrent
T6	WNK3	NON_SYNONYMOUS_CODING	T1043S	polymorphism	Passenger	19.05%	19.82%	4190	4586	1.00	1.00	X	54275654	T	A				Non-Pathogenic	Non-Pathogenic	Ion Torrent
T6	XYLB	NON_SYNONYMOUS_CODING	Q328E	polymorphism	Passenger	20.29%	23.94%	2444	3379	1.00	1.00	3	38416728	C	G				Non-Pathogenic	Non-Pathogenic	Ion Torrent
T6	ZNF185	NON_SYNONYMOUS_CODING	R596C	polymorphism	Passenger	21.59%	28.11%	2084	1594	1.00	1.00	X	152134061	C	T				Non-Pathogenic	Non-Pathogenic	Ion Torrent
T6	ZSWIM7	NON_SYNONYMOUS_CODING	H105Q	disease_causing	Passenger	29.85%	35.19%	4466	4706	1.00	1.00	17	15881469	A	T				Pathogenic	Pathogenic	Ion Torrent
T6	STIP1	NON_SYNONYMOUS_CODING	E350Q	disease_causing	Passenger	0.00%	18.69%	2260	2755	NA	NA	11	63967436	G	C				Pathogenic	Pathogenic	Ion Torrent
