# Supplementary material for: Intra-tumor genetic heterogeneity and alternative driver genetic alterations in breast cancers with heterogeneous HER2 gene amplification
Source: Genome Biol. 2015 May 22;16(1):107. doi: 10.1186/s13059-015-0657-6 (PMC4440518; doi:10.1186/s13059-015-0657-6)

Additional file 10

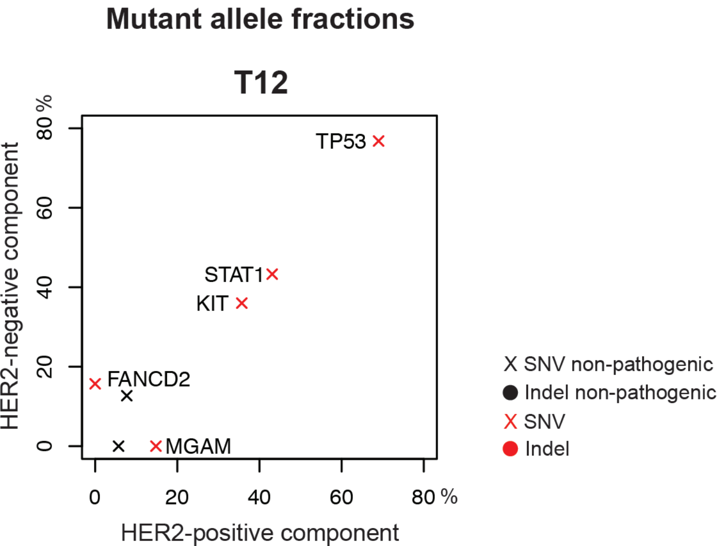

Supplement: Additional file 10: — Mutations validated in the HER2-positive and HER2-negative components of case T12 using targeted capture massively parallel sequencing. Allelic fractions of mutations identified in HER2-positive and HER2-negative components of the HER2 heterogeneous breast cancer T12 subjected to targeted capture massively parallel sequencing using a panel of 273 genes comprising genes frequently mutated in breast cancer and DNA repair-related genes. Indel, insertion and deletion; SNV, single nucleotide variant. [file 13059_2015_657_MOESM10_ESM.pdf]
