# Supplementary material for: Intra-tumor genetic heterogeneity and alternative driver genetic alterations in breast cancers with heterogeneous HER2 gene amplification
Source: Genome Biol. 2015 May 22;16(1):107. doi: 10.1186/s13059-015-0657-6 (PMC4440518; doi:10.1186/s13059-015-0657-6)
Supplement: Additional file 11: — Somatic mutations restricted to HER2-negative components of HER2 heterogeneous breast cancers identified by massively parallel sequencing. [file 13059_2015_657_MOESM11_ESM.pdf]

Additional file 11. Somatic mutations restricted to HER2-negative components of HER2 heterogeneous breast cancers identified by massively parallel sequencing.

Case	Gene Symbol	Consequence	Amino acid change	MutationTaster	CHASM (breast)	CHROM	POS	REF	ALT	LOH in HER2-negative component	Mutant allele fraction in HER2-negative component	Number of reference reads	Number of alternate reads	Pathogenic by predictor algorithms	Cancer Gene Census	127 genes Kandoth et al	Cancer5000S	Overall Pathogenic	Sequencing platform
T6	ETV5	NON_SYNONYMOUS_CODING	E60K	disease_causing	Passenger	3	185823241	C	T	Unknown	14.48%	5970	1037	Pathogenic	YES			Pathogenic	WES (Illumina) followed by amplicon sequencing (Ion Torrent)
T8	BRAF	NON_SYNONYMOUS_CODING	P403S	disease_causing	Passenger	7	140482928	G	A	No LOH	3.80%	175	7	Pathogenic	YES	YES	YES	Pathogenic	Targeted hybrid capture sequencing (Illumina)
T6	GPHN	NON_SYNONYMOUS_CODING	Q690H	disease_causing	Passenger	14	67646384	G	C	No LOH	16.95%	1016	180	Pathogenic	YES			Pathogenic	WES (Illumina) followed by amplicon sequencing (Ion Torrent)
T6	ERBB2	NON_SYNONYMOUS_CODING	I767M	disease_causing	Driver	17	37880257	C	G	No LOH	13.77%	5535	1621	Pathogenic	YES		YES	Pathogenic	WES (Illumina) followed by amplicon sequencing (Ion Torrent)
T6	BRD4	NON_SYNONYMOUS_CODING	E4D	disease_causing	Passenger	19	15383899	C	G	No LOH	17.48%	4798	949	Pathogenic	YES			Pathogenic	WES (Illumina) followed by amplicon sequencing (Ion Torrent)
T3	ATRX	SPLICE_SITE_ACCEPTOR		disease_causing		X	76949427	CT	AG	No LOH	13.20%	46	7	Pathogenic	YES	YES		Pathogenic	Targeted hybrid capture sequencing (Illumina)
T6	FBXO6	ESSENTIAL_SPLICE_SITE		disease_causing		1	11731984	G	A	No LOH	4.79%	7116	2503	Pathogenic				Pathogenic	WES (Illumina) followed by amplicon sequencing (Ion Torrent)
T6	LRRC41	NON_SYNONYMOUS_CODING	S615C	polymorphism	Passenger	1	46746145	G	C	No LOH	19.46%	5391	1033	Non-Pathogenic				Non-Pathogenic	WES (Illumina) followed by amplicon sequencing (Ion Torrent)
T6	ZYG11A	NON_SYNONYMOUS_CODING	E32K	disease_causing	Passenger	1	53320140	G	A	Unknown	14.31%	5528	923	Pathogenic				Pathogenic	WES (Illumina) followed by amplicon sequencing (Ion Torrent)
T6	ROR1	NON_SYNONYMOUS_CODING	Q812H	disease_causing	Passenger	1	64644160	G	C	No LOH	23.43%	917	233	Pathogenic				Pathogenic	WES (Illumina) followed by amplicon sequencing (Ion Torrent)
T6	ARHGEF11	NON_SYNONYMOUS_CODING	M79I	disease_causing	Passenger	1	156950265	C	T	Unknown	11.01%	3043	621	Pathogenic				Pathogenic	WES (Illumina) followed by amplicon sequencing (Ion Torrent)
T6	DPYSL5	NON_SYNONYMOUS_CODING	E448K	disease_causing	Passenger	2	27165520	G	A	No LOH	13.22%	2930	966	Pathogenic				Pathogenic	WES (Illumina) followed by amplicon sequencing (Ion Torrent)
T6	SLC41A3	NON_SYNONYMOUS_CODING	K280N	polymorphism	Passenger	3	125735624	C	G	Unknown	16.74%	3109	740	Non-Pathogenic				Non-Pathogenic	WES (Illumina) followed by amplicon sequencing (Ion Torrent)
T6	ACPL2	NON_SYNONYMOUS_CODING	R290T	disease_causing	Passenger	3	141011473	G	C	No LOH	20.86%	4913	1295	Pathogenic				Pathogenic	WES (Illumina) followed by amplicon sequencing (Ion Torrent)
T6	FAM188B2	SPLICE_SITE				3	150600849	G	C	No LOH	20.26%	2086	330						WES (Illumina) followed by amplicon sequencing (Ion Torrent)
T8	GPR98	NON_SYNONYMOUS_CODING	G1218W	disease_causing	Passenger	5	89949043	G	T	Unknown	7.00%	80	6	Pathogenic				Pathogenic	Targeted hybrid capture sequencing (Illumina)
T6	NRG2	NON_SYNONYMOUS_CODING	R462W	disease_causing	Passenger	5	139232521	G	A	No LOH	14.57%	2869	878	Pathogenic				Pathogenic	WES (Illumina) followed by amplicon sequencing (Ion Torrent)
T4	COL12A1	NON_SYNONYMOUS_CODING	R265H	disease_causing	Passenger	6	75898962	C	T	No LOH	11.40%	31	4	Pathogenic				Pathogenic	Targeted hybrid capture sequencing (Illumina)
T6	FAM185A	NON_SYNONYMOUS_CODING	S248L	disease_causing	Passenger	7	102401808	C	T	Unknown	20.53%	2575	436	Pathogenic				Pathogenic	WES (Illumina) followed by amplicon sequencing (Ion Torrent)
T6	SLC37A3	SPLICE_SITE		polymorphism		7	140043205	C	A	No LOH	21.76%	2240	515	Non-Pathogenic				Non-Pathogenic	WES (Illumina) followed by amplicon sequencing (Ion Torrent)
T1	PLEC	NON_SYNONYMOUS_CODING	E1057V	disease_causing	Passenger	8	145003978	T	A	No LOH	3.80%	877	35	Pathogenic				Pathogenic	Targeted hybrid capture sequencing (Illumina)
T6	UNC13B	NON_SYNONYMOUS_CODING	E362K	disease_causing	Passenger	9	35313903	G	A	No LOH	19.07%	1244	400	Pathogenic				Pathogenic	WES (Illumina) followed by amplicon sequencing (Ion Torrent)
T6	CACNB2	NON_SYNONYMOUS_CODING	P221A	disease_causing	Passenger	10	18795467	C	G	LOH	9.59%	2309	489	Pathogenic				Pathogenic	WES (Illumina) followed by amplicon sequencing (Ion Torrent)
T11	NRP1	NON_SYNONYMOUS_CODING	R767H	disease_causing	Passenger	10	33475179	C	T	No LOH	7.75%	7007	1993	Pathogenic				Pathogenic	WES (Illumina) followed by amplicon sequencing (Ion Torrent)
T6	MARCH8	NON_SYNONYMOUS_CODING	P254A	polymorphism	Passenger	10	45953803	G	C	Unknown	13.66%	1799	522	Non-Pathogenic				Non-Pathogenic	WES (Illumina) followed by amplicon sequencing (Ion Torrent)
T6	ZSWIM8	NON_SYNONYMOUS_CODING	S704C	polymorphism	Passenger	10	75552408	C	G	No LOH	15.17%	4704	841	Non-Pathogenic				Non-Pathogenic	WES (Illumina) followed by amplicon sequencing (Ion Torrent)
T6	STIP1	NON_SYNONYMOUS_CODING	E350Q	disease_causing	Passenger	11	63967436	G	C	No LOH	18.69%	3498	176	Pathogenic				Pathogenic	WES (Illumina) followed by amplicon sequencing (Ion Torrent)
T6	ZNF202	NON_SYNONYMOUS_CODING	D285H	disease_causing	Passenger	11	123598283	C	G	Unknown	24.33%	5011	1509	Pathogenic				Pathogenic	WES (Illumina) followed by amplicon sequencing (Ion Torrent)
T6	RARG	NON_SYNONYMOUS_CODING	E40K	disease_causing	Passenger	12	53621212	C	T	No LOH	22.49%	6743	746	Pathogenic				Pathogenic	WES (Illumina) followed by amplicon sequencing (Ion Torrent)
T6	CBX5	NON_SYNONYMOUS_CODING	S14L	disease_causing	Passenger	12	54651394	G	A	No LOH	14.44%	3082	327	Pathogenic				Pathogenic	WES (Illumina) followed by amplicon sequencing (Ion Torrent)
T6	NBEA	NON_SYNONYMOUS_CODING	L1750V	disease_causing	Passenger	13	35770321	C	G	LOH	26.02%	1966	508	Pathogenic				Pathogenic	WES (Illumina) followed by amplicon sequencing (Ion Torrent)
T6	DYNC1H1	NON_SYNONYMOUS_CODING	L2315F	disease_causing	Passenger	14	102478738	G	C	Unknown	14.80%	5818	886	Pathogenic				Pathogenic	WES (Illumina) followed by amplicon sequencing (Ion Torrent)
T6	DYNC1H1	NON_SYNONYMOUS_CODING	E4148K	disease_causing	Passenger	14	102509014	G	A	Unknown	22.65%	3568	507	Pathogenic				Pathogenic	WES (Illumina) followed by amplicon sequencing (Ion Torrent)
T6	ZNF609	NON_SYNONYMOUS_CODING	S849C	disease_causing	Passenger	15	64967599	C	G	No LOH	23.14%	702	162	Pathogenic				Pathogenic	WES (Illumina) followed by amplicon sequencing (Ion Torrent)
T6	CHD9	NON_SYNONYMOUS_CODING	G403E	disease_causing	Passenger	16	53191209	G	A	Unknown	24.79%	1982	479	Pathogenic				Pathogenic	WES (Illumina) followed by amplicon sequencing (Ion Torrent)
T6	ATP8B1	NON_SYNONYMOUS_CODING	R768G	disease_causing	Passenger	18	55329831	T	C	Unknown	16.51%	865	107	Pathogenic				Pathogenic	WES (Illumina) followed by amplicon sequencing (Ion Torrent)
T6	RTTN	NON_SYNONYMOUS_CODING	S2060C	disease_causing	Passenger	18	67684885	G	C	No LOH	17.85%	4623	1286	Pathogenic				Pathogenic	WES (Illumina) followed by amplicon sequencing (Ion Torrent)
T6	CYP2S1	NON_SYNONYMOUS_CODING	D260H	polymorphism	Passenger	19	41704737	G	C	Unknown	12.44%	3982	672	Non-Pathogenic				Non-Pathogenic	WES (Illumina) followed by amplicon sequencing (Ion Torrent)
T6	PSG5	NON_SYNONYMOUS_CODING	N333S	polymorphism	Passenger	19	43674257	T	C	Unknown	16.08%	2757	599	Non-Pathogenic				Non-Pathogenic	WES (Illumina) followed by amplicon sequencing (Ion Torrent)
T8	XRCC1	NON_SYNONYMOUS_CODING	S236F	disease_causing	Passenger	19	44057138	G	A	Unknown	14.60%	386	66	Pathogenic				Pathogenic	Targeted hybrid capture sequencing (Illumina)
T6	EVA1C	NON_SYNONYMOUS_CODING	S87L	disease_causing	Passenger	21	33825719	C	T	Unknown	15.05%	4141	661	Pathogenic				Pathogenic	WES (Illumina) followed by amplicon sequencing (Ion Torrent)
T11	PTTG1IP	NON_SYNONYMOUS_CODING	R127W	polymorphism	Passenger	21	46276178	G	A	No LOH	21.70%	27140	7522	Non-Pathogenic				Non-Pathogenic	WES (Illumina) followed by amplicon sequencing (Ion Torrent)
T6	SEPT3	NON_SYNONYMOUS_CODING	T356I	polymorphism	Passenger	22	42392961	C	T	Unknown	9.96%	3913	787	Non-Pathogenic				Non-Pathogenic	WES (Illumina) followed by amplicon sequencing (Ion Torrent)
T6	TBC1D22A	NON_SYNONYMOUS_CODING	H451D	disease_causing	Passenger	22	47507425	C	G	No LOH	19.23%	5381	1268	Pathogenic				Pathogenic	WES (Illumina) followed by amplicon sequencing (Ion Torrent)
T6	GLRA4	NON_SYNONYMOUS_CODING	D59N	disease_causing	Passenger	X	102979853	C	T	LOH	18.75%	5622	959	Pathogenic				Pathogenic	WES (Illumina) followed by amplicon sequencing (Ion Torrent)
T8	DOCK11	NON_SYNONYMOUS_CODING	C1702Y	disease_causing	Passenger	X	117805014	G	A	No LOH	14.60%	181	31	Pathogenic				Pathogenic	Targeted hybrid capture sequencing (Illumina)
T11	HCFC1	NON_SYNONYMOUS_CODING	V687L	disease_causing	Passenger	X	153223307	C	G	No LOH	10.66%	7058	842	Pathogenic				Pathogenic	WES (Illumina) followed by amplicon sequencing (Ion Torrent)
T12	ADAM29	NON_SYNONYMOUS_CODING	A513T	disease_causing	Passenger	4	175898213	G	A	No LOH	17.80%	148	32	180				Pathogenic	WES (Illumina)
T12	BPI	NON_SYNONYMOUS_CODING	S145L	polymorphism	Passenger	20	36938940	C	T	No LOH	8.20%	681	61	742				Non-Pathogenic	WES (Illumina)
T12	FANCD2	NON_SYNONYMOUS_CODING	L1394F	disease_causing	Passenger	3	10138153	G	T	No LOH	13.90%	124	20	144			YES	Pathogenic	WES (Illumina) followed by targeted sequencing (Illumina)
T12	KRTAP10-2	NON_SYNONYMOUS_CODING	C42S	disease_causing	Passenger	21	45971218	A	T	LOH	8.60%	53	5	58				Pathogenic	WES (Illumina)
T12	LINGO1	NON_SYNONYMOUS_CODING	A560V	polymorphism	Passenger	15	77906570	G	A	No LOH	25.70%	130	45	175				Non-Pathogenic	WES (Illumina)
T12	LRFN1	NON_SYNONYMOUS_CODING	R214H	disease_causing	Passenger	19	39805336	C	T	No LOH	4.20%	733	32	765				Pathogenic	WES (Illumina)
T12	NASP	NON_SYNONYMOUS_CODING	P372T	polymorphism	Passenger	1	46073697	C	A	LOH	17.90%	23	5	28				Non-Pathogenic	WES (Illumina)
T12	OR2T4	NON_SYNONYMOUS_CODING	H86Y	polymorphism	Passenger	1	248525138	C	T	No LOH	11.30%	63	8	71				Non-Pathogenic	WES (Illumina)
T12	OR2T4	NON_SYNONYMOUS_CODING	A85T	polymorphism	Passenger	1	248525135	G	A	No LOH	11.80%	60	8	68				Non-Pathogenic	WES (Illumina)
T12	PPIAL4G	NON_SYNONYMOUS_CODING	A128V	polymorphism	Passenger	1	143767466	G	A	No LOH	16.70%	30	6	36				Non-Pathogenic	WES (Illumina)
T12	PROS1	NON_SYNONYMOUS_CODING	R330Q	polymorphism	Passenger	3	93611943	C	T	No LOH	7.60%	145	12	157				Non-Pathogenic	WES (Illumina)
T12	RTN3	NON_SYNONYMOUS_CODING	P528L	disease_causing	Passenger	11	63487557	C	T	No LOH	24.40%	167	54	221				Pathogenic	WES (Illumina)
T12	SEMA3E	NON_SYNONYMOUS_CODING	R337Q	polymorphism	Passenger	7	83032081	C	T	No LOH	20.10%	111	28	139				Non-Pathogenic	WES (Illumina)
T12	SGTB	NON_SYNONYMOUS_CODING	T209I	polymorphism	Passenger	5	64976376	G	A	LOH	13.60%	209	33	242				Non-Pathogenic	WES (Illumina)
T12	SLC31A1	NON_SYNONYMOUS_CODING	D37N	polymorphism	Passenger	9	116018537	G	A	No LOH	20.20%	198	50	248				Non-Pathogenic	WES (Illumina)
T12	TTN	NON_SYNONYMOUS_CODING	T14842S	polymorphism	Passenger	2	179490023	G	C	No LOH	21.30%	200	54	254				Non-Pathogenic	WES (Illumina)
T12	ZBTB24	NON_SYNONYMOUS_CODING	H684Y	disease_causing	Passenger	6	109787098	G	A	No LOH	14.80%	282	49	331				Pathogenic	WES (Illumina)
T12	ZNF492	NON_SYNONYMOUS_CODING	T409I	disease_causing	.	19	22847697	C	T	LOH	14.30%	48	8	56				Pathogenic	WES (Illumina)
T12	ZNF566	STOP_GAINED	R273*	disease_causing	Passenger	19	36940322	G	A	No LOH	14.90%	212	37	249				Pathogenic	WES (Illumina)
