# Supplementary material for: Intra-tumor genetic heterogeneity and alternative driver genetic alterations in breast cancers with heterogeneous HER2 gene amplification
Source: Genome Biol. 2015 May 22;16(1):107. doi: 10.1186/s13059-015-0657-6 (PMC4440518; doi:10.1186/s13059-015-0657-6)
Supplement: Additional file 19: — Supplementary methods. [file 13059_2015_657_MOESM19_ESM.pdf]

ADDITIONAL FILE 19 - SUPPLEMENTARY METHODS

Microarray-based comparative genomic hybridization analysis

Two-dimensional Loess regression was used to normalize the data for spatial and intensity-dependent biases. Log_2 ratios were then smoothed using circular binary segmentation (cbs) and arrays were median centered [1]. Segments were classified as amplification (>0.4), gain (>0.08 and ≤0.4) and loss (<-0.08) or no change.

To allow a direct comparison between paired arrays of HER2-positive and HER2-negative components, the aCGH signals were scaled using the formula $s=log2(R/c - (1-c)/c)$, where R is the observed log ratio and c is the proportion of tumor cells [2]. The parameter c was estimated using the ratio of the median of the cbs-smoothed segments where the absolute value of the log_2 ratio for the segments were >0.1 between the *HER2*-amplified component and its matched *HER2*-non-amplified component. After scaling, signal of each non-amplified component was subtracted from its matched amplified component and median centered. Log_2 ratios were smoothed using cbs and gains (>0.08) and losses (<-0.08) were defined.

Statistical analysis

Categorical data obtained from the analysis of the HER2-positive and the HER2-negative components of each case were subjected to a multi-Fisher's exact test with adjustment for multiple testing, using the step-down permutation procedure maxT, which provides strong control of the family-wise type I error rate (FWER), as previously described [1, 3]. Unsupervised cluster analysis of the aCGH states (i.e. gains, losses, amplifications) was performed using Ward's clustering algorithm with Euclidean distance metric.

Identification of copy-number regulated genes in HER2-negative components

To identify possible driver events in the HER2-negative components of HER2 heterogeneous breast cancers, regions of high confidence amplification identified to be restricted to the HER2-negative components were assessed in the luminal breast cancers of the TCGA dataset [4] using cBioPortal (www.cBioPortal.org) [5]. Copy number regulation was assessed using t-tests comparing the mRNA expression z-scores between neutral/ no copy number change and high-level amplification, adjusted for multiple testing using the Benjamini-Hochberg method in the TCGA luminal breast cancer cohort [4]. In addition to statistical difference, candidate genes were further narrowed down by requiring the mean expression of the neutral/no copy number change group to be smaller than the mean expression of the amplified group minus 2 s.d.s, and the mean expression of the amplified group to be greater than the mean expression of the neutral/no copy number change group plus 2 s.d.s.

Whole exome sequencing

Sequence alignment was performed using the Burrow-Wheeler Alignment tool (BWA) [6] against the human reference genome GRCh37. Local-realignment and duplicate removal were performed using the picard (http://picard.sourceforge.net./). Variants were called using GATK Unified Genotyper [7]. Low-quality variants and common SNPs (>5% of minor allele frequency in at least one population according to dbSNP v132) were removed. Germline variants were subsequently removed, where a minimum sequencing depth of 10 and 5, for normal and tumour respectively, were required for variants to be accepted. Furthermore, a variant was only considered somatic if the variant was not observed in the matched normal.

Amplicon sequencing

PCR primers for the somatic variants found by exome sequencing to be present at an allelic fraction of at least 15% were designed using Primer 3 (http://biotools.umassmed.edu/bioapps/primer3_www.cgi). Sequencing template was prepared using the OneTouch emulsion PCR followed by OneTouch enrichment system, and run on a 316 100Mb chip according to manufacturer's instructions (Ion Torrent, Life Technologies). Raw sequences were trimmed at the threshold of q>17 to a minimum sequence length of 50bp. Trimmed sequences were aligned to the human reference genome GRCh37 using Bowtie 2 [8] and local realignment was performed using GATK [7]. Somatic variants were called using Varscan 2 [9].

Targeted massively parallel sequencing

Custom oligonucleotides (NimblegenSeqCap) were designed for hybridization capture of all protein-coding exons of 273 genes either recurrently mutated in breast cancer or DNA repair-related genes as previously described [10]. Sequence alignment was performed using BWA [6] against the human reference genome GRCh37. Somatic single nucleotide variants were identified using MuTect [11]. Somatic insertions and deletions (indels) were identified using GATK Haplotype Caller [7] and manually reviewed using the Integrative Genome Viewer [12] as previously described [13].

Classification of mutations

A single nucleotide variant (SNV) was classified as non-pathogenic if it was called non-pathogenic by both computational mutation function prediction algorithms MutationTaster [14] and CHASM (breast) v1.0.7 [15] as described [16]. In addition, we investigated if the SNVs identified targeted one of the 127 cancer genes described by Kandoth et al. [17], the 513 genes included in the Sanger Cancer Gene Census (http://cancer.sanger.ac.uk/cancergenome/projects/census/) or the Cancer5000-S genes [18].

Cell culture conditions

Cell lines were maintained in a 5% CO_2-humidified atmosphere at 37°C in the following culture media: MCF10A and MCF12A cells in Dulbecco's modified Eagle's medium (DMEM)/F12 supplemented with 5% horse serum, 20 ng/ml EGF, 10 μg/ml insulin, 0.5 μg/ml hydrocortisone, and 1% penicillin/streptomycin (P/S) [13, 19]; HEK293T and NIH3T3 cells in DMEM supplemented with 10% FBS and 1% P/S; MCF7 cell in DMEM, BT474 and T47D cells in RMPI-1640, all supplemented with 10% FBS and 1% P/S. Conditioned media from MCF7 and T47D cells (stably expressing HER2 wild-type, HER2 I767M or empty vector) was prepared by serum-starving the cells overnight. Starvation medium was removed, and fresh complete medium was added to the cells (1 ml of medium was used per 10 cm^2 well). After 18 h, medium was passed through a 2-μm filter and proteins were precipitated by mixing the lysates with 4 volumes of cold acetone, incubating at -20°C for 60 min and centrifuging for 10 min at 15,000 x g.

Vector construction, mutagenesis, transformation and plasmid preparation

The human ERBB2 (NM_004448) cDNA ORF clone pCMV6-ERBB2::Myc-DDK was purchased from Origene (RC212583), and the I767M mutation was introduced using the GeneArt Site Directed Mutagenesis Kit (Life Technologies) following manufacturer's recommendations. The C-terminal Myc-DKK tag of pCMV6-Myc-DDK vector was replaced with TagRFP (Evrogen) and ZsGreen (Clontech) genes to create the pCMV6-TagRFP and pCMV6-ZsGreen fluorescent expression vectors, respectively. *ERBB2 (HER2)* wild-type and mutant (I767M) open reading frames were cloned into the pCMV6-TagRFP vector to generate pCMV6-ERBB2::TagRFP and pCMV6-ERBB2(I767M)::TagRFP plasmids, respectively, and into the pLenti-EF1a-GFP-2A-Puro vector (LV067, ABM) to generate the pLenti-ERBB2 and pLenti-ERBB2(I767M) lentiviral plasmids. The latter plasmids co-express both the target gene and the bicistron GFP-2A-Puro from separate promoters; GFP (green fluorescent protein) expression was used to track resistant cells. The *ERBB2* Y835F and V777L mutations were introduced in the pCMV6-ERBB2::TagRFP using the Q5 Site-Directed Mutagenesis Kit (New England Biolabs) following manufacturer's recommendations. *BRF2* and *DSN1* open reading frames were amplified from total RNA derived from a healthy donor using SuperScript III First Strand Synthesis System and Platinum Taq polymerase High Fidelity (Life Technologies), and cloned into the pCMV6-ZsGreen vector to generate pCMV6-BRF2::ZsGreen and pCMV6-DSN1::ZsGreen plasmids, respectively, and into the pLenti-EF1a-GFP-2A-Puro vector (LV067, ABM), generating the pLenti-BRF2 and pLenti-DSN1 lentiviral plasmids, respectively. Sanger sequencing was used to confirm the reading frames of the wild-type *ERBB2*, the *ERBB2* mutants, and wild-type *BRF2* and *DSN1* (for primers see Additional file 21). pCMV6-TagRFP/ZsGreen-derived and pLenti-EF1a-GFP-2A-Puro-derived plasmids were transformed into OneShot Top10 and Stabl3 chemically competent *E. coli* cells

(Life Technologies), respectively. Highly pure plasmids were prepared using the Qiagen Plasmid Midi kit (Qiagen) and DNA Clean & Concentrator (Zymo Research).

Transfections of mammalian cells and analysis of transgene expression

Transfections of empty vector, wild-type *BRF2, DSN1* and *ERBB2,* and I767M mutant *ERBB2* were performed essentially as previously described [13]. One day before transfection, cells were plated in 500 µl of growth media without antibiotics for cells to be ~80% confluent at the time of transfection. 750 ng of highly purified plasmids were diluted in 100 µl Opti-MEM I Reduced Serum Medium (Gibco, Life Technologies) without serum, and 0.75 µl of PLUS reagent (Life Technologies) was added to the diluted plasmid and incubated for 5 min at room temperature. Then, 1.75 µl Lipofectamine LTX (Life Technologies) was added directly to the diluted plasmid/PLUS and incubated for 30 min at room temperature, and 100 µl of the plasmid-Lipofectamine LTX complexes was added to the cells, mixed by gently rocking and incubated at 37 °C, 5% CO_2. The media were changed after 4 h. For transient experiments (immunofluorescence analysis and immunoprecipitation, see below), analysis was performed 48 h post-transfection. For stable selection using the pLenti-EF1a-GFP-2A-Puro-derived plasmids, cells were harvested 60 h after transfection, diluted 1/10 and plated on 100 mm dishes with fresh media containing puromycin (Life Technologies) at the corresponding killing dose for each cell line (0.5 µg/ml MCF7, 1 µg/ml T47D/MCF10A/MCF12A and 1.25 µg/ml NIH3T3). The media containing puromycin was changed every other day. Resistant colonies formed at 15-20 days of selection. The expression of transgenes in stable clones for DSN1 and BRF2 was evaluated at the mRNA level by qualitative and quantitative RT-PCR, given that antibodies producing satisfactory western blot results could not be obtained. The expression of wild-type and I767M mutant HER2 proteins was confirmed by western blotting. The expression of transgenes from pCMV-ZsGreen/TagRFP-derived plasmids was visually evaluated 48 h after transfection using a Nikon Eclipse Ti fluorescence microscope.

Growth curves

T47D and MCF7 cells stably expressing HER2, HER2(I767M) and vector control cells (T47D, 1000 cells/well and MCF7, 500 cells/well) were seeded in the corresponding normal growth medium in 96-well plates in triplicate as previously described [13]. After 16 h, media were replaced either with the normal growth media supplemented with vehicle (PBS) or human neuregulin-1 (hNRG-1, 10 ng/ml; Cell Signaling Technologies). Cell growth was monitored over a course of six days, assessing cell viability every 24 h by incubation with CellTiter-Blue (Promega) for 2 h. Fluorescence readings were performed using the Victor X4 Multimode Plate Reader (PerkinElmer). Growth curves were plotted and analyzed (multiple t-tests, corrected for multiple comparisons using the Holm-Šídák method, alpha: 0.05) using GraphPad Prism v_6.0c.

Qualitative and quantitative RT-PCR

Given the lack of robust antibodies against BRF2 and DSN1 for western blotting, the expression levels of these genes were assessed at the mRNA level by qualitative and quantitative reverse transcription (RT)-PCR. Total RNA was extracted from stably transfected MCF10A and NIH3T3 cell lines using the RNeasy Mini Kit (Qiagen). RNA quantification was performed using the Qubit Fluorometer (Life Technologies), and 200 ng of DNAse-treated, total RNA was reverse transcribed using the SuperScript VILO cDNA Synthesis Kit (Invitrogen), with triplicate reactions performed for each sample. For qualitative analysis of gene expression, 22-cycle PCR reactions were performed on cDNA derived from the stably transfected cell lines and loaded on a 1.5 % agarose gel. GAPDH was used as loading control.

Quantitative RT-PCR was performed using TaqMan Assay-on-Demand (IDs: Hs00217757_m and Hs01566636_m1 for BRF2 and DSN1, respectively, Applied Biosystems) on the StepOnePlus Real-Time PCR System (Applied Biosystems). Since the efficiencies of amplification of these commercial probes are close to 100% (Life Technologies), the calculations were performed using the comparative ΔΔCt method for quantitation, relative to cells stably expressing the empty vector (calibrator samples). Two reference genes, TFRC (Hs00174609_m1-TFRC) and MRPL19 (Hs00608522_g1-MRPL19), were employed for assay normalization as previously described [20]. Target gene expression levels were normalized to the geometric mean of the two reference genes. For MCF7 and T47D cells, quantitative RT-PCR was performed for NRG1 (Assay on Demand IDs: Hs00247620_m1; Life Technologies), using GAPDH (Assay on Demand IDs: Hs99999905_m1) for assay normalization, as described above. All reactions were performed in triplicate.

Western blotting

Protein lysates were prepared using the M-PER Mammalian Protein Extraction Reagent supplemented with Halt Protease and Phosphatase inhibitors cocktail (Thermo Scientific) and cleared by centrifugation at 14,000 x g for 15 min at 4 °C. Standard western blotting was conducted as previously described [21], using antibodies against HER2 (MS-730, Thermo Scientific; 06-562, Millipore), phospho-HER2 (Tyr1248; Millipore), AKT (Cell Signaling Technology), phospho-AKT (Ser473; Cell Signaling Technology), p44/42 MAPK (ERK1/2; Cell Signaling Technology), phospho-p44/42 MAPK (ERK1/2; Thr202/Tyr204; Cell Signaling Technology), S6 Ribosomal Protein (Cell Signaling Technology), phospho-S6 Ribosomal Protein (Ser235/236; Cell Signaling Technology), PRAS40 (Cell Signaling Technology), Neuregulin-1 (Abcam), α-tubulin (Cell Signaling Technology) and TagRFP (AB234, Evrogen). Conjugated IRDye680RD/800CW secondary antibodies (LI-COR

Biosciences) were detected using the Odyssey Infrared Imaging System (LI-COR Biosciences) [21]. Quantification of conjugated secondary antibodies and analysis were performed using the Image Studio Software from LI-COR (LI-COR Biosciences). For detection of protein post-immunoprecipitation assays TrueBlot IgG IRDye800 secondary antibodies were used (Rockland). When required, stripping of the membranes was done using the Restore PLUS Western Blot Stripping Buffer (Thermo Scientific). For neuregulin-1 experiments, 48 h post-transfection MCF7 and T47D cells expressing HER2 wild-type, HER2(I767M) and vector control were treated with human hNRG-1 (10 ng/ml) (or vehicle PBS) for 20 min, then washed 5 times with PBS to remove hNRG-1 (withdrawal) and placed back into the incubator (see below). Protein lysates were prepared prior to hNRG-1 treatment (i.e. baseline), after 20 min of hNRG1 treatment, and 30 min, 3 h, 5 h and 24 h post hNRG-1 withdrawal. Forty micrograms of total protein lysates were loaded for this analysis. For quantification, normalized phospho/total signals were used to determine the phospho-protein changes post hNGR-1 withdrawal.

Tyrosine kinase assay

Tyrosine kinase activity was evaluated using the ADP Hunter HS Assay (DiscoveRx) and the Tyrosine Kinase Assay Kit (colorimetric detection, Millipore) essentially as previously described [13]. The ADP Hunter HS Assay employs a non-radioactive method to detect the amount of ADP produced as a result of enzyme activity, which is directly proportional to enzyme phosphotransferase activity, and was performed according to the manufacturer's guidelines. In brief, the immunocomplex-magnetic bead pellets were resuspended in 40 µl ADP Assay Buffer supplemented with 50 µM ATP and 400 ng/ml Poly(Glu4-Tyr) biotin conjugate [GG(EEEEY)10EE)] (Millipore); autophosphorylation was evaluated by omitting Poly(Glu4-Tyr) in the reaction. The reactions proceeded for 30 min at 30°C with vigorous agitation to maintain the beads in suspension, and were terminated by placing the tubes in a magnetic stand and removing the supernatant for the ADP detection step. Fluorescence readings (Excitation/emission: 530/590 nm) were taken after a 20 min incubation using the Victor X4 Multimode Plate Reader. All reactions were run in triplicates. In addition, an ELISA-based colorimetric Tyrosine Kinase Assay Kit was employed to detect the protein tyrosine phosphotransferase activity. The protocol for assaying immobilized enzymes was used as recommended by the manufacturers. In brief, the immunocomplexes-magnetic bead pellets were resuspended in 50 µl 1x Tyrosine Kinase reaction Buffer, 1 mM Sodium Orthovanadate and 400 ng/ml Poly(Glu4-Tyr)-biotin (Millipore). The reactions proceeded for 30 min at 30 °C with vigorous agitation to maintain the beads in suspension, and were terminated by reducing the pH to 2 with the addition of 2 µl of 5 M hydrochloric acid and placing the tubes in a magnetic stand to remove the supernatant. The supernatants were then transferred to individual wells of streptavidin coated strip plates and incubated for 1 h at 37 °C. Phosphotyrosine detection was performed by reading the absorbance at 450 nm using the Victor X4 Multimode Plate

Reader. Analysis was carried out using GraphPad Prism® v_6.0c (multiple t-tests, corrected for multiple comparisons using the Holm-Šídák method, alpha: 0.05). Following the kinase assay, the immunocomplexes-magnetic beads were then resuspended in 30 µl 2x NuPAGE LDS Sample Buffer (Life Technologies), heated for 5 min at 95 °C and placed in a magnetic rack. The supernatant (IP/eluted protein post-kinase assay) was resolved on 4–12% gradient NuPAGE Novex Bis-Tris gels (LifeTechnologies) by electrophoresis, transferred onto nitrocellulose membranes and analyzed by western blotting using antibodies against HER2 and tagRFP, as described above.

Three-dimensional (3D) matrigel cultures

MCF10A and MCF12A cells transiently expressing HER2 wild-type, HER2(I767M), BRF2 and DSN1 proteins, as well as vector control cells, were seeded on top of growth factor-reduced reconstituted basement membrane (Matrigel, BD Biosciences) in 100 µl medium supplemented with 2% Matrigel in 8-well chamber slides, and grown in a 5% CO2-humidified atmosphere at 37 °C as previously described [13, 19]. At day 14, phase contrast images of acinar structures were acquired (EVOS XL Imaging System, Life Technologies). Cells were fixed using methanol:acetone (1:1) for 12 min at -20 °C and stained with 300 nM DAPI solution. Extraction of acinar MCF10 and MCF12A structures from 3D cultures was performed using ice-cold PBS-EDTA as described previously [22]. The dissolved Matrigel/3D cell structure solution was then transferred onto a glass slide, mounted for confocal microscopy [22], and confocal images of the midsection of acinar-like spheroids obtained using a Leica TCS SP5-II Upright microscope (Leica). For quantification, acinar structures of ≥200µm and ≥250µm diameter for MCF12A and MCF10A cells, respectively, were counted in Image J. All experiments were performed in triplicate. In addition, MCF10A stably expressing HER2 wild-type, HER2(I767M) and empty vector were grown on top of growth factor-reduced reconstituted basement membrane as described above [19]. Acinar structures were fixed at day 20 with 4% formalin and stained with DAPI solution (ProLong antifade; Life Technologies). Acinar size measurements were determined from phase contrast images using the MetaMorph Image Analysis software (Molecular Devices) and expressed in mm^3 following the equation $V = (Dxd^2)/2$. Confocal images of MCF10A cells stably expressing empty vector, wild-type HER2 and HER2 I767M were assessed for lumen filling: cleared lumen (<3 intraluminal cells); filled lumen (>6 intraluminal cells) and intermediate (3-5 intraluminal cells). The number of cleared, intermediate and filled acini was normalized to the total number of acinar structures counted. Phase contrast and fluorescence imaging was performed using Axio2 wide-field (Zeiss) and TCS SP5-II Upright microscopes (Leica).

SUPPLEMENTARY REFERENCES

1. Duprez R, Wilkerson PM, Lacroix-Triki M, Lambros MB, Mackay A, Hern RA, Gauthier A, Pawar V, Colombo PE, Daley F, et al: **Immunophenotypic and genomic characterization of papillary carcinomas of the breast.** *J Pathol* 2012, **226:**427-441.
2. van de Wiel MA, Kim KI, Vosse SJ, van Wieringen WN, Wilting SM, Ylstra B: **CGHcall: calling aberrations for array CGH tumor profiles.** *Bioinformatics* 2007, **23:**892-894.
3. Marchio C, Natrajan R, Shiu KK, Lambros MB, Rodriguez-Pinilla SM, Tan DS, Lord CJ, Hungermann D, Fenwick K, Tamber N, et al: **The genomic profile of HER2-amplified breast cancers: the influence of ER status.** *J Pathol* 2008, **216:**399-407.
4. The Cancer Genome Atlas Network: **Comprehensive molecular portraits of human breast tumours.** *Nature* 2012, **490:**61-70.
5. Gao J, Aksoy BA, Dogrusoz U, Dresdner G, Gross B, Sumer SO, Sun Y, Jacobsen A, Sinha R, Larsson E, et al: **Integrative analysis of complex cancer genomics and clinical profiles using the cBioPortal.** *Sci Signal* 2013, **6:**pl1.
6. Li H, Durbin R: **Fast and accurate long-read alignment with Burrows-Wheeler transform.** *Bioinformatics* 2010, **26:**589-595.
7. McKenna A, Hanna M, Banks E, Sivachenko A, Cibulskis K, Kernytsky A, Garimella K, Altshuler D, Gabriel S, Daly M, DePristo MA: **The Genome Analysis Toolkit: a MapReduce framework for analyzing next-generation DNA sequencing data.** *Genome Res* 2010, **20:**1297-1303.
8. Langmead B, Salzberg SL: **Fast gapped-read alignment with Bowtie 2.** *Nat Methods* 2012, **9:**357-359.
9. Koboldt DC, Zhang Q, Larson DE, Shen D, McLellan MD, Lin L, Miller CA, Mardis ER, Ding L, Wilson RK: **VarScan 2: somatic mutation and copy number alteration discovery in cancer by exome sequencing.** *Genome Res* 2012, **22:**568-576.
10. Natrajan R, Wilkerson PM, Marchio C, Piscuoglio S, Ng CK, Wai P, Lambros MB, Samartzis EP, Dedes KJ, Frankum J, et al: **Characterization of the genomic features and expressed fusion genes in micropapillary carcinomas of the breast.** *J Pathol* 2014, **232:**553-565.
11. Cibulskis K, Lawrence MS, Carter SL, Sivachenko A, Jaffe D, Sougnez C, Gabriel S, Meyerson M, Lander ES, Getz G: **Sensitive detection of somatic point mutations in impure and heterogeneous cancer samples.** *Nat Biotechnol* 2013, **31:**213-219.
12. Robinson JT, Thorvaldsdottir H, Winckler W, Guttman M, Lander ES, Getz G, Mesirov JP: **Integrative genomics viewer.** *Nat Biotechnol* 2011, **29:**24-26.
13. Weinreb I, Piscuoglio S, Martelotto LG, Waggott D, Ng CK, Perez-Ordonez B, Harding NJ, Alfaro J, Chu KC, Viale A, et al: **Hotspot activating PRKD1 somatic mutations in**

polymorphous low-grade adenocarcinomas of the salivary glands. *Nat Genet* 2014, **46:**1166-1169.

14. Schwarz JM, Cooper DN, Schuelke M, Seelow D: **MutationTaster2: mutation prediction for the deep-sequencing age.** *Nat Methods* 2014, **11:**361-362.
15. Carter H, Chen S, Isik L, Tyekucheva S, Velculescu VE, Kinzler KW, Vogelstein B, Karchin R: **Cancer-specific high-throughput annotation of somatic mutations: computational prediction of driver missense mutations.** *Cancer Res* 2009, **69:**6660-6667.
16. Martelotto LG, Ng C, De Filippo MR, Zhang Y, Piscuoglio S, Lim R, Shen R, Norton L, Reis-Filho JS, Weigelt B: **Benchmarking mutation effect prediction algorithms using functionally validated cancer-related missense mutations.** *Genome Biol* 2014, **15:**484.
17. Kandoth C, McLellan MD, Vandin F, Ye K, Niu B, Lu C, Xie M, Zhang Q, McMichael JF, Wyczalkowski MA, et al: **Mutational landscape and significance across 12 major cancer types.** *Nature* 2013, **502:**333-339.
18. Lawrence MS, Stojanov P, Mermel CH, Robinson JT, Garraway LA, Golub TR, Meyerson M, Gabriel SB, Lander ES, Getz G: **Discovery and saturation analysis of cancer genes across 21 tumour types.** *Nature* 2014, **505:**495-501.
19. Debnath J, Muthuswamy SK, Brugge JS: **Morphogenesis and oncogenesis of MCF-10A mammary epithelial acini grown in three-dimensional basement membrane cultures.** *Methods* 2003, **30:**256-268.
20. Piscuoglio S, Ng CK, Martelotto LG, Eberle CA, Cowell CF, Natrajan R, Bidard FC, De Mattos-Arruda L, Wilkerson PM, Mariani O, et al: **Integrative genomic and transcriptomic characterization of papillary carcinomas of the breast.** *Mol Oncol* 2014, **Epub ahead of print**.
21. Weigelt B, Warne PH, Downward J: **PIK3CA mutation, but not PTEN loss of function, determines the sensitivity of breast cancer cells to mTOR inhibitory drugs.** *Oncogene* 2011, **30:**3222-3233.
22. Lee GY, Kenny PA, Lee EH, Bissell MJ: **Three-dimensional culture models of normal and malignant breast epithelial cells.** *Nat Methods* 2007, **4:**359-365.
