# Supplementary material for: Intra-tumor genetic heterogeneity and alternative driver genetic alterations in breast cancers with heterogeneous HER2 gene amplification
Source: Genome Biol. 2015 May 22;16(1):107. doi: 10.1186/s13059-015-0657-6 (PMC4440518; doi:10.1186/s13059-015-0657-6)
Supplement: Additional file 20: — R script and code. [file 13059_2015_657_MOESM20_ESM.pdf]

```
############################################################################
# This script accompanies Ng et al, Intra-tumor genetic heterogeneity and alternative driver
# genetic alterations in breast cancers with HER2 heterogeneous gene amplification
# Last updated: 22nd October 2014
# Author: Charlotte Ng
############################################################################

# The complete version of scripts, annotation files, raw data files
# and sample sheets can be found:
# https://dl.dropboxusercontent.com/u/15115364/Ng_et_al_HER2_heterogeneity.zip

####### REQUIRED FILES ######
# BACE.eset.97_CN.R
# HER2.heterogeneity.design.txt
# HER2.heterogeneity.grade.pheno.txt
# annotation files
# GPR files
##############################

source("BACE.eset.97_CN.R")
library(gplots)


################## additional functions ################################

doAllGenomePlots <- function(cgh, outdir){
	if (!file.exists(outdir)) { dir.create(outdir)}

	### single genome plots
	for (i in sampleNames(cgh)) {

		png(file=paste(outdir, "/",i,".genomes.png",sep=""),width=1200, height=400)
		genomePlot(cgh, case=i, thresh=c(0.08,0.4), yAxis=c(-2,2), colourGL=T, main=i)
		dev.off()
	}

	#### single chromosome 17 plots
	for (i in sampleNames(cgh)) {
		c =17

		png(file=paste(outdir, "/",i,".chr",c,".png",sep=""),width=300, height=300)
		genomePlot(cgh, case=i, chroms=c, thresh=c(0.08,0.4), yAxis=c(-2,2), colourGL=T, main=i)
		dev.off()
	}
}

doAllGenomePlotsPdf <- function(cgh, outdir){
	if (!file.exists(outdir)) { dir.create(outdir)}

	### single genome plots
	for (i in sampleNames(cgh)) {

		pdf(file=paste(outdir, "/",i,".genomes.pdf",sep=""),width=12, height=4)
		genomePlot(cgh, case=i, thresh=c(0.08,0.4), yAxis=c(-2,2), colourGL=T, main="")
		dev.off()
	}

	#### single chromosome 17 plots
	for (i in sampleNames(cgh)) {
		c =17

		pdf(file=paste(outdir, "/",i,".chr",c,".pdf",sep=""),width=4, height=4)
		genomePlot(cgh, case=i, chroms=c, thresh=c(0.08,0.4), yAxis=c(-2,2), colourGL=T, main="")
		dev.off()
	}
}

doAllSubtractedGenomePlots <- function(cgh, outdir){
	if (!file.exists(outdir)) { dir.create(outdir)}

	### single genome plots
	for (i in sampleNames(cgh)) {

		png(file=paste(outdir, "/",i,".genomes.png",sep=""),width=1200, height=300)
		genomePlot(cgh, case=i, thresh=c(0.08,0.4), yAxis=c(-2,2), colourGL=T, main=i)
		dev.off()
	}

	#### single chromosome 17 plots
	for (i in sampleNames(cgh)) {
		c =17

		png(file=paste(outdir, "/",i,".chr",c,".png",sep=""),width=600, height=300)
		genomePlot(cgh, case=i, chroms=c, thresh=c(0.08,0.4), yAxis=c(-2,2), colourGL=T, main=i)
		dev.off()
	}
}

estimateCorrectionFactor <- function (cgh, threshold=0.1) {
	meds <- numeric()
	for (i in unique(pData(cgh)$pairs)) {
		t<- assayData(cgh)$smo[,which(pData(cgh)$pairs==i)]
		if (ncol(t)==2) {
			meds <- c(meds,(median(apply(t[which((t[,1]>threshold &
			t[,2]>threshold)|(t[,1]< (-1*threshold) & t[,2]< (-1*threshold))),],1,function(x){x[2]/x[1]}))))
		} else {
			meds <- c(meds,(median(apply(t[which((t[,1]> threshold & t[,2]> threshold)|
			(t[,1]< (-1*threshold) & t[,2]< (-1*threshold))),],1,function(x){x[2]/x[1]}))),
			(median(apply(t[which((t[,1]> threshold & t[,3]> threshold)|(t[,1]< (-1*threshold) &
```

```
                        t[,3]< (-1*threshold))),],1,function(x){x[3]/x[1]}))))
                }
        }

        correction = rep(1,length(pData(cgh)$pairs))
        correction[which(duplicated(pData(HER2.heterogeneity.cbs.rescaled)$pairs))] <- meds
        correction=unlist(tapply(correction,as.factor(pData(HER2.heterogeneity.cbs.rescaled)$pairs),function(x){x/max(x)}))
        correction
}

adjustForCellularity <- function(matrix, cellularity) {
        cat("Adjusting for cellularity ... \n")
        result <- c()
        adjustCellularity <- function(value, cellularity) {
                corrected <- (2^value/cellularity - (1 - cellularity)/cellularity)
                if (!is.na(corrected) & corrected < 2^(-5)) {
                        corrected <- 2^value
                }
                new.value <- log2(corrected)
                return(new.value)
        }
        for (i in 1:ncol(matrix)) {
                cat("Cellularity sample", i, ": ", cellularity[i],
                        "\n")
                if (cellularity[i] < 1) {
                        new.column <- sapply(matrix[, i], adjustCellularity, cellularity[i])
                        result <- cbind(result, new.column)
                }
                else {
                        result <- cbind(result, matrix[, i])
                }
        }
        return(result)
}

################## END additional functions #################################


########## START of analysis - reading in data, segmentation, cellularity correction ############

# read in the CGH files listed in the sample sheet
samplesheet = "HER2.heterogeneity.design.txt"
phenofile = "HER2.heterogeneity.grade.pheno.txt"
HER2.heterogeneity.raw <- dietCGH(samplesheet, subtractBG=F, MAD=2, rhm=T, fdata.file="Ann32K.assembly55.filt.Eset.txt")
HER2.heterogeneity.raw <- readPheno(HER2.heterogeneity.raw, phenofile)

# remove chromosome Y
HER2.heterogeneity.raw <- HER2.heterogeneity.raw[-which(fData(HER2.heterogeneity.raw)$chrom == 24),]
HER2.heterogeneity.raw <- HER2.heterogeneity.raw[,order(HER2.heterogeneity.raw$pairs,HER2.heterogeneity.raw$HER2)]

# segment copy number using CBS, call CGH states
HER2.heterogeneity.cbs.raw <- cbsCGH(HER2.heterogeneity.raw, undo.splits="sdundo", undo.SD=1.5, min.width=3)
HER2.heterogeneity.cbs.raw <- callCGHStatesThreshold(HER2.heterogeneity.cbs.raw, gainthresh=0.08, ampthresh=0.4,
delthresh=-100, contig=3)

# rescale by MAD
HER2.heterogeneity.cbs.raw <- calculateMAD(HER2.heterogeneity.cbs.raw)
HER2.heterogeneity.cbs.rescaled <- rescaleCGHtoMAD(HER2.heterogeneity.cbs.raw, rescale=0.1)
HER2.heterogeneity.cbs.rescaled <- callCGHStatesThreshold(HER2.heterogeneity.cbs.rescaled, gainthresh=0.08, ampthresh=0.4,
delthresh=-100, contig=3)
HER2.heterogeneity.cbs.rescaled <- calculateMAD(HER2.heterogeneity.cbs.rescaled)

# correct for cellularity between matched samples by scaling up the sample with narrower dynamic range
correction <- estimateCorrectionFactor(HER2.heterogeneity.cbs.rescaled, 0.1)
HER2.heterogeneity.cbs.rescaled.corrected <- HER2.heterogeneity.cbs.rescaled
exprs(HER2.heterogeneity.cbs.rescaled.corrected) <- adjustForCellularity(exprs(HER2.heterogeneity.cbs.rescaled),correction)
HER2.heterogeneity.cbs.rescaled.corrected <- calculateMAD(HER2.heterogeneity.cbs.rescaled.corrected)

HER2.heterogeneity.cbs.rescaled.corrected <- cbsCGH(HER2.heterogeneity.cbs.rescaled.corrected, undo.splits="sdundo",
undo.SD=1.5, min.width=3)
HER2.heterogeneity.cbs.rescaled.corrected <- callCGHStatesThreshold(HER2.heterogeneity.cbs.rescaled.corrected,
gainthresh=0.08, ampthresh=0.4, delthresh=-100, contig=3)

# correct for cellularity for T2
correction <- rep(1, ncol(HER2.heterogeneity.cbs.rescaled.corrected))
correction[which(pData(HER2.heterogeneity.cbs.rescaled.corrected)$pair==2)] <- 0.6
HER2.heterogeneity.cbs.rescaled.corrected.T2corrected <- HER2.heterogeneity.cbs.rescaled.corrected
exprs(HER2.heterogeneity.cbs.rescaled.corrected.T2corrected) <-
adjustForCellularity(exprs(HER2.heterogeneity.cbs.rescaled.corrected.T2corrected),correction)
HER2.heterogeneity.cbs.rescaled.corrected.T2corrected <- calculateMAD(HER2.heterogeneity.cbs.rescaled.corrected.T2corrected)

HER2.heterogeneity.cbs.rescaled.corrected.T2corrected <- cbsCGH(HER2.heterogeneity.cbs.rescaled.corrected.T2corrected,
undo.splits="sdundo", undo.SD=1.5, min.width=3)
HER2.heterogeneity.cbs.rescaled.corrected.T2corrected <-
callCGHStatesThreshold(HER2.heterogeneity.cbs.rescaled.corrected.T2corrected, gainthresh=0.08, ampthresh=0.4,
delthresh=-100, contig=3)

# make genome-wide copy number plots
doAllGenomePlots(HER2.heterogeneity.cbs.rescaled.corrected.T2corrected, "genome.plots.rescaled.corrected.T2corrected")
doAllGenomePlotsPdf(HER2.heterogeneity.cbs.rescaled.corrected.T2corrected, "genome.plots.rescaled.corrected.T2corrected")

# write raw data to file
spitTables(HER2.heterogeneity.cbs.rescaled.corrected.T2corrected, "HER2.rescaled.corrected.T2corrected",
output.directory="tables.HER2.rescaled.corrected.T2corrected")

# make additional copy number figures for specific chromosomes and samples
pdf("HER2.T2.Amp.chr8.pdf", height=5, width=8)
genomePlot(HER2.heterogeneity.cbs.rescaled.corrected.T2corrected, main="", chroms=8, case="HER2.T2.Amp", thresh=c(0.08,0.4),
yAxis=c(-2,3), colourGL=T)
```

```r
dev.off()
pdf("HER2.T2.Not-Amp.chr8.pdf", height=5, width=8)
genomePlot(HER2.heterogeneity.cbs.rescaled.corrected.T2corrected, main="", chroms=8, case="HER2.T2.Not-Amp",
thresh=c(0.08,0.4), yAxis=c(-2,3), colourGL=T)
dev.off()
pdf("HER2.T4.Amp.chr8.pdf", height=5, width=8)
genomePlot(HER2.heterogeneity.cbs.rescaled.corrected.T2corrected, main="", chroms=8, case="HER2.T4.Amp", thresh=c(0.08,0.4),
yAxis=c(-1.5,1.5), colourGL=T)
dev.off()
pdf("HER2.T4.Not-Amp.chr8.pdf", height=5, width=8)
genomePlot(HER2.heterogeneity.cbs.rescaled.corrected.T2corrected, main="", chroms=8, case="HER2.T4.Not-Amp",
thresh=c(0.08,0.4), yAxis=c(-1.5,1.5), colourGL=T)
dev.off()
pdf("HER2.T12.Amp.chr20.pdf", height=5, width=8)
genomePlot(HER2.heterogeneity.cbs.rescaled.corrected.T2corrected, main="", chroms=20, case="HER2.T12.Amp",
thresh=c(0.08,0.4), yAxis=c(-2,2), colourGL=T)
dev.off()
pdf("HER2.T12.Not-Amp.chr20.pdf", height=5, width=8)
genomePlot(HER2.heterogeneity.cbs.rescaled.corrected.T2corrected, main="", chroms=20, case="HER2.T12.Not-Amp",
thresh=c(0.08,0.4), yAxis=c(-2,2), colourGL=T)
dev.off()

## spits out the copy number alterations to files in sample.GALS directory
listBreaksGL(HER2.heterogeneity.cbs.rescaled.corrected.T2corrected, contig=3, project="HER2.rescaled.corrected.T2corrected")

############ grouped analysis - Fisher's test for amp vs non-amp ##############

HER2.heterogeneity.grouped=HER2.heterogeneity.cbs.rescaled.corrected.T2corrected[,which(HER2.heterogeneity.cbs.rescaled.corr
ected.T2corrected$HER2 %in% c("Amp","Not-Amp"))]
HER2.heterogeneity.grouped.FE <- fisherTestCGH(HER2.heterogeneity.grouped, pheno=HER2.heterogeneity.grouped$HER2,
project="HER2.heterogeneity")
listBreaksFisher(HER2.heterogeneity.grouped.FE, project="HER2.grouped")
latticePlotFishers(HER2.heterogeneity.grouped.FE, project="HER2.grouped")

############################## Make heatmaps ###############################

HER2.cols <- c("black", "white", brewer.pal(12, "Paired")[1:2], brewer.pal(12, "Paired")[5:6], brewer.pal(3, "Greens"),
brewer.pal(6, "Dark2"), brewer.pal(6, "Set3"))

plotPhenoBar(HER2.heterogeneity.grouped, assayData(HER2.heterogeneity.grouped)$GL, dist.method="euclidean",
phenotypes=pData(HER2.heterogeneity.grouped)[,c(2,5,6,4,3)], project="HER2.euclidean.ward", device="PDF",
pheno.colours=HER2.cols)

# make clustering heatmap using Euclidean distance as the distance metric and ward clustering algorithm
pdf("HER2.aCGH.euclidean.ward.pdf", width=6, height=9)
cghHeatmap(HER2.heterogeneity.grouped, project="HER2.euclidean.ward", main="HER2.aCGH", dist.method="euclidean",
phenotypes=pData(HER2.heterogeneity.grouped)[,c(3,6,7,5,4)], pheno.colours=HER2.cols, plot.sample.names=F)
dev.off()

png("HER2.aCGH.euclidean.ward.png", width=400, height=600)
par(cex.lab=2)
cghHeatmap(HER2.heterogeneity.grouped, project="HER2.euclidean.ward", main="HER2.aCGH", dist.method="euclidean",
phenotypes=pData(HER2.heterogeneity.grouped)[,c(2,5,6,4,3)], pheno.colours=HER2.cols, plot.sample.names=F)
dev.off()

############ Paired comparisons - differential copy number alterations ##############

HER2.heterogeneity.subtracted <- HER2.heterogeneity.cbs.rescaled.corrected.T2corrected[,
        which(pData(HER2.heterogeneity.cbs.rescaled.corrected.T2corrected)$HER2=="Amp")]
t1 <- HER2.heterogeneity.cbs.rescaled.corrected.T2corrected[,
        which(pData(HER2.heterogeneity.cbs.rescaled.corrected.T2corrected)$HER2=="Not-Amp")]

# subtract the signal from the non-amplified components from the amplified components
assayDataElement(HER2.heterogeneity.subtracted, "exprs") <- exprs(HER2.heterogeneity.subtracted)-exprs(t1)

sampleNames(HER2.heterogeneity.subtracted) <- paste(sampleNames(HER2.heterogeneity.subtracted),sampleNames(t1),sep="-")
rm(t1)

HER2.heterogeneity.subtracted <- cbsCGH(HER2.heterogeneity.subtracted, undo.splits="sdundo", undo.SD=1.5, min.width=3)
HER2.heterogeneity.subtracted <- calculateMAD(HER2.heterogeneity.subtracted)
HER2.heterogeneity.subtracted <- rescaleCGHtoMAD(HER2.heterogeneity.subtracted, rescale=0.1)
HER2.heterogeneity.subtracted <- callCGHStatesThreshold(HER2.heterogeneity.subtracted, gainthresh=0.08, ampthresh=0.4,
contig=3)

# generate the genome-wide copy number figures of the differential copy number alterations
for (i in sampleNames(HER2.heterogeneity.subtracted)) {

        outdir="genome.plots.subtracted"
        if (!file.exists(outdir)) { dir.create(outdir)}
        png(file=paste(outdir, "/",i,".subtracted",".png",sep=""),width=1200, height=300)
        genomePlot(HER2.heterogeneity.subtracted, case=i, thresh=c(0.08,0.45), yAxis=c(-2,2), colourGL=T, main=i)
        dev.off()
}

# write out the raw data and the results to tables
spitTables(HER2.heterogeneity.subtracted, "HER2.corrected.subtracted", output.directory="HER2.corrected.subtracted")
listBreaksGL(HER2.heterogeneity.subtracted, contig=3, project="HER2.corrected.subtracted")
```
