# Supplementary material for: Intra-tumor genetic heterogeneity and alternative driver genetic alterations in breast cancers with heterogeneous HER2 gene amplification
Source: Genome Biol. 2015 May 22;16(1):107. doi: 10.1186/s13059-015-0657-6 (PMC4440518; doi:10.1186/s13059-015-0657-6)
Supplement: Additional file 21: — Sanger sequencing primers used in this study. [file 13059_2015_657_MOESM21_ESM.pdf]

**Additional file 21**. Sanger sequencing primers used in this study.

| Primers | Sequence | Strand |
|---|---|---|
| TP53 exon 1 | GAAAATACACGGAGCCGAGAG | Forward |
| | GTGCCAGGAGCCTCGCAG | Reverse |
| TP53 exon 2 | ACAGGTCTCTGCTAGGGGG | Forward |
| | CTTCTCTGCAGGCCCAGG | Reverse |
| TP53 exon 3 | GTGAAAAGAGCAGTCAGAGGA | Forward |
| | CAGCCCCCTAGCAGAGACC | Reverse |
| TP53 exon 4(1) | ACGGCCAGGCATTGAAGT | Forward |
| | GTCCAGATGAAGCTCCCAGA | Reverse |
| TP53 exon 4(2) | ATGACAGGGGCCAGGAGG | Forward |
| | CTGAGGACCTGGTCCTCTGA | Reverse |
| TP53 exon 5 | CTGGGCAACCAGCCCTGT | Forward |
| | TGTTCACTTGTGCCCTGACTT | Reverse |
| TP53 exon 6 | GACAACCACCCTTAACCCCTC | Forward |
| | CTGGTTGCCCAGGGTCCC | Reverse |
| TP53 exon 7 | GAGGCTGGGGCACAGCAG | Forward |
| | GGTCTCCCCAAGGCGCACT | Reverse |
| TP53 exon 8 | CATAACTGCACCCTTGGTCTC | Forward |
| | GGACCTGATTTCCTTACTGCC | Reverse |
| TP53 exon 9 | GTGTTAGACTGGAAACTTTCCACTT | Forward |
| | CAGTTATGCCTCAGATTCACTTTT | Reverse |
| TP53 exon 10 | GGGGAGTAGGGCCAGGAA | Forward |
| | ACTGTGTATATACTTACTTCTCCCCC | Reverse |
| TP53 exon 11 | GGAGAGATGGGGGTGGGA | Forward |
| | CCTCTCACTCATGTGATGTCATC | Reverse |
| HER2-Wt/I767M | TGAGGAAGGTGAAGGTGCTT | Forward |
| | TCCCGGACATGGTCTAAGAG | Reverse |
| HER2-V777L | GGATCTGGCGCTTTTGGC | Forward |
| | ACACCAGTTCAGCAGGTCCTG | Reverse |
| HER2-Y835 | TCTGGGCATCTGCCTGAC | Forward |
| | CCCATCTGCATGGTACTCTGT | Reverse |
| BRF2_Fs1 | TGGTGTGCTCCGACTGCGGCT | Forward |
| BRF2_Fs2 | GCTGCGTCTTAATCACCTGCC | Forward |
| BRF2_Fs3 | TGTGCTTGGCAGAACTGGTGA | Forward |
| BRF2_Fs4 | CGTGGCTGGTGACCGGGAGGC | Forward |
| BRF2_Fs5 | CCGCTCTGCCTTTCGGGATGG | Forward |
| BRF2_Fs6 | AGAGAGCCCAGGCTGCTAGAC | Forward |
| BRF2_Rs1 | AGCCGCAGTCGGAGCACACCA | Reverse |
| BRF2_Rs2 | GGCAGGTGATTAAGACGCAGC | Reverse |
| BRF2_Rs3 | TCACCAGTTCTGCCAAGCACA | Reverse |
| BRF2_Rs4 | GCCTCCCGGTCACCAGCCACG | Reverse |
| BRF2_Rs5 | CCATCCCGAAAGGCAGAGCGG | Reverse |
| BRF2_Rs6 | GTCTAGCAGCCTGGGCTCTCT | Reverse |
| DSN1_Fs1 | AGTCTCAGTCCTGTGGAAGTG | Forward |
| DSN1_Fs2 | CCAGTTATCAAGACAGGAGGC | Forward |
| DSN1_Fs3 | TCAGCCGGTCTATCAGTGTCG | Forward |
| DSN1_Fs4 | GATTCAAATGGAAAAGCATCAG | Forward |
| DSN1_Fs5 | AAATTACTGAGGTCAAAGTGGA | Forward |
| DSN1_Fs6 | TGGTGATGGATGAACTGCAAGG | Forward |
| DSN1_Rs1 | CACTTCCACAGGACTGAGACT | Reverse |
| DSN1_Rs2 | GCCTCCTGTCTTGATAACTGG | Reverse |
| DSN1_Rs3 | CGACACTGATAGACCGGCTGA | Reverse |
| DSN1_Rs4 | CTGATGCTTTTCCATTTGAATC | Reverse |
| DSN1_Rs5 | TCCACTTTGACCTCAGTAATTT | Reverse |
| DSN1_Rs6 | CCTTGCAGTTCATCCATCACCA | Reverse |
